# Improving Energy Access, Climate and Socio‐Economic Outcomes Through Off‐Grid Electrification Technologies: A Systematic Review

**DOI:** 10.1002/cl2.70060

**Published:** 2025-08-15

**Authors:** Cem Yavuz, Zafeer Ravat, María Daniela Anda León, Sanghwa Lee, Paulo Fernandes, Quinn Reifmesser, Frederick Elliott Gaved, Samantha Pilato, Constanza Gonzalez Parrao, Birte Snilstveit

**Affiliations:** ^1^ International Initiative for Impact Evaluation (3ie) London UK; ^2^ University of East Anglia (UEA) Norwich UK; ^3^ Sustainable Energy for All (SEforALL) Vienna Austria

## Abstract

Halfway through the final decade of actions towards the 2030 Sustainable Development Goals (SDGs), progress toward SDG7 is off track. It is estimated that by 2030, 660 million people, mainly rural populations within Sub‐Saharan Africa, will be without electricity. One promising avenue to counteract this trend is the provision of decentralised, or off‐grid, renewable energy. Our systematic review synthesised the rigorous evidence evaluating off‐grid electrification interventions and provides policymakers, practitioners and researchers across the sustainable energy field with an updated and comprehensive analysis of the impact of off‐grid electrification interventions. Our systematic review synthesised the available rigorous evidence on the effects of off‐grid technologies in low‐ and middle‐income countries. We assessed which off‐grid interventions are effective at supporting access, climate and socio‐economic development outcomes, how these effects vary by region, population and other intervention characteristics and the main challenges and facilitators for interventions to benefit participants. We conducted a systematic search in 18 academic databases and 29 grey literature sources. We supplemented our searches by conducting backward and forward citation tracking, publishing a call for additional studies and contacting subject experts. To identify additional qualitative studies, we performed additional searches for studies related to interventions from our included impact evaluations. We included experimental and quasi‐experimental impact evaluations of interventions promoting the use and uptake of off‐grid technologies in low‐ and middle‐income countries. Interventions fell into one of four categories: the *direct provision* of technologies, the *opportunity to purchase/market expansion* of technologies, *subsidies and credit* to purchase technologies, *information provision* promoting the use of technologies. Studies in any language were included, though they must have been published since 2000. We also included qualitative studies to understand the main challenges and facilitators of intervention effectiveness. Quantitative data was extracted for all estimates deemed relevant and the risk of bias for each of these estimates was assessed independently by two reviewers. When data allowed us to do so, we calculated standardised mean differences for results from each study and used random effects meta‐analysis to synthesise effectiveness findings for comparable outcomes. We provided forest plots and measures of heterogeneity for all outcomes and tested for publication bias in outcomes with more than 10 effect sizes. When feasible, we conducted moderator analysis to understand how effects varied by intervention characteristics and checked whether results were sensitive to the risk of bias score of estimates. For qualitative studies, we extracted and analysed data based on a previously developed framework for challenges and facilitators of sustainable energy interventions. Our review includes 47 impact evaluations, with the majority of studies conducted in Sub‐Saharan Africa. Most studies evaluated an intervention either directly providing technology or providing participants financial support to purchase the technology. Solar home systems, solar lamps/lanterns and solar mini‐grids were the most commonly implemented technologies. Our risk of bias assessment found that the majority of the evidence base, for both experimental and quasi‐experimental studies, was of a high risk of bias. Spill‐overs, cross‐overs and contamination was the main bias dimension found in experimental studies, while confounding bias was most prevalent in quasi‐experimental studies. For energy access outcomes, we found that interventions significantly reduced kerosene consumption and increased the use and uptake of off‐grid technologies while also leading to a small increase in energy access. However, we found no statistically significant effect on other energy security measures, such as reliability and affordability, as well as lighting use and energy expenditure. We identified very few studies evaluating a climate‐related outcome and found no significant effect on air pollution. For socio‐economic outcomes, we found a small positive effect on income, time spent studying and women's empowerment. However, evidence of publication bias in evaluations of time spent studying suggests that this result should be interpreted with caution, as it may overestimate the true effect. We found no statistically significant effects on other measures of time allocation, school attendance and respiratory illnesses. Our qualitative results were derived from 19 qualitative and mixed‐methods studies related to interventions evaluated across our included impact evaluations. We found that financial support is an important mechanism by which interventions may aim to increase the uptake and use of off‐grid technologies. Local involvement in interventions was found to be a success factor, while information and marketing strategies were highlighted in multiple qualitative studies as a key factor in increasing participant engagement. Our systematic review synthesised the rigorous evidence on the effects of off‐grid electrification interventions and can be used by policymakers, practitioners and researchers to inform decision‐making on sustainable energy. Future research in this area should focus on filling gaps in the evidence base. Further evidence is needed in contexts outside of Sub‐Saharan Africa, as well as on the impact of information provision. Our analysis, limited in many instances due to the small number and quality of studies, found positive effects across different energy access and socio‐economic outcomes. Qualitative analysis highlighted the importance of ensuring that interventions are appropriate for local contexts and that local views and voices are built into intervention design.

**Abbreviations**
CAPS: Cooking and Pneumonia Study
CH_4_
: methane
CO_2_
: carbon dioxide
DEP: Development Evidence Portal
EGM: evidence gap map
ERSEN: Rural Electrification Senegal
GHG: greenhouse gas
GRADE: Grading of Recommendations, Assessment, Development, and Evaluation
ICS: improved cookstoves
IE: impact evaluation
LAC: Latin American and the Caribbean
LMIC: low‐ and middle‐income country
N_2_O: nitrous oxide
NGO: non‐governmental organisation
NOX: nitrogen oxides
PM2.5: particulate matter
PRISMA: Preferred Reporting Items for Systematic reviews and Meta‐Analyses
PV: photovoltaic
PVPA: Photovoltaic Poverty Alleviation
QED: quasi‐experimental design
RCT: randomised controlled trial
SDG: Sustainable Development Goal
SEforALL: Sustainable Energy for All
SHS: solar‐home systems
SMD: standardised mean differences
SMG: solar market garden
SOX: sulphur oxides
SSA: Sub‐Saharan Africa
USD: United States dollar

## 1 Plain Language Summary

Off‐grid technologies increase income, time spent studying, and reduce kerosene consumption across low‐ and middle‐income countries (LMICs), though more research is needed around climate‐related outcomes (e.g., air quality).

### 1.1 The Review in Brief

Our review included 47 rigorous impact evaluations of off‐grid technology interventions in LMICs. The evidence was limited for many outcomes and was assessed as having a high risk of bias. Based on this evidence, we found that off‐grid technology interventions reduce kerosene consumption and have a small increase in income and women's empowerment. The interventions may also increase time spent studying; however, this result should be interpreted with caution, as there was evidence of publication bias, suggesting a potential overestimation of the true effect. We did not identify significant effects on other outcomes. Based on qualitative evidence, the financial model, local involvement and information and technical knowledge were identified as key factors that can contribute to programme effectiveness. The review findings constitute a global call for investment in rigorous evaluations in the sustainable energy sector.

### 1.2 What Is the Review About?

Halfway through the final decade of action towards the 2030 Sustainable Development Goals (SDGs), progress towards achieving universal energy for all (SDG7) is faltering, with many rural areas continuing to lack access to electricity. With limited financing options, it is vital that available resources are put towards interventions that work.

We defined off‐grid technologies as those that provide electricity to households and communities without connection to a centralised grid. We included any intervention aimed at increasing the uptake and use of said technologies.

### 1.3 What Is the Aim of This Review?

Our review assessed the effects of off‐grid technologies on outcomes related to energy access, climate and socio‐economic indicators. We also aimed to identify barriers and facilitators that could affect the success of off‐grid interventions.

### 1.4 What Are the Main Findings of This Review?

#### 1.4.1 Included Studies

We included 47 rigorous impact evaluations, primarily focusing on Sub‐Saharan Africa (SSA). We included experimental and quasi‐experimental designs (QEDs), though the majority were assessed as having a high risk of bias. The extent of evidence available across outcomes varied widely, hence for some of them, we were unable to draw conclusive findings.

#### 1.4.2 Effects of Off‐Grid Technologies on Energy Access Outcomes

We found that off‐grid technology interventions had a significant effect on reducing kerosene consumption (the only traditional fuel for which there was comparable data available) as well as on increasing the use of off‐grid technologies, though these findings are based on six and five studies, respectively. We found no statistically significant effect on other energy access outcomes.

#### 1.4.3 Effects of Off‐Grid Technologies on Climate Outcomes

We identified five studies, predominantly with high risk of bias, reporting the effect of off‐grid technologies on air quality up to 24 months post‐intervention, but did not find a statistically significant effect. Only one study analysed the effect of off‐grid technologies on CO_2_ emissions, so no conclusions could be drawn.

#### 1.4.4 Effects of Off‐Grid Technologies on Socio‐Economic Outcomes

Off‐grid technologies had a small positive effect on income, a finding derived from 13 studies across nine countries. We also found an increase in time spent studying, based on 13 studies, though this result may be overstated due to a risk of publication bias. There was no significant effect on school attendance or test scores. Based on three studies, we found a significant increase in women's decision‐making. No significant effects were identified for other socio‐economic outcomes.

#### 1.4.5 Main Barriers and Facilitators to Off‐Grid Technology Effectiveness

Drawing from qualitative evidence, we identified three key aspects to increasing engagement and effectiveness of programmes: information on how and why technologies should be used, allowing avenues for local involvement in the implementation and design of programmes, and setting appropriate financial models.

### 1.5 What Do the Findings of This Review Mean?

The results of this review suggest that information is a key mechanism to ensuring engagement and sustainability of interventions and should be considered a valuable component when designing programmes.

The state of the evidence on this topic, which did not allow us to explore the effects of off‐grid interventions extensively, highlights that more and better research is needed. Future research could build up the evidence in contexts outside of SSA. Along with embedding high‐quality evaluations within programme design and budgets, research should also aim to use standardised outcome measures to ensure that findings across contexts can be compared.

### 1.6 How Up‐to‐Date Is This Review?

The review searched for relevant studies up to September 2023.

## 2 Background

### 2.1 The Problem, Condition or Issue

Halfway through the final decade of actions towards the 2030 SDGs, progress towards ensuring universal access to affordable, reliable and modern energy services is off‐track (SEforALL 2024). While a further 48 countries achieved universal electricity access between 2010 and 2020, population growth and other factors such as COVID‐19 have led to faltering progress, indicating a global regression in progress towards universal electrification (IEA, IRENA, UNSD, World Bank, and WHO 2024; SEforALL 2024). It is estimated that by 2030, 660 million people, mainly within SSA, will remain without electricity access if current trends continue (IEA, IRENA, UNSD, World Bank, and WHO 2024).

One promising avenue to increase electricity access is decentralised, or off‐grid, renewable energy. Off‐grid systems offer quicker access to clean and sustainable energy, especially for those living in remote areas where grid extension is not viable (Mandelli et al. 2016). Recent global discussions have underscored the role of off‐grid connections in achieving universal access to affordable and clean energy by 2030 (IEA 2023, 2024), while also avoiding the expansion of coal‐produced energy. Off‐grid solar home systems (SHS) could provide reliable electricity in rural SSA, and according to the International Energy Agency (IEA), nearly two‐thirds of new connections in Africa are expected from stand‐alone and mini‐grid systems, aiming for universal access by 2030 (IEA 2023, 2024).

The rapid provision of clean and renewable energy through off‐grid systems can have a productive use, leading to new and improved income‐generating opportunities in LMICs, accelerating economic development. Refrigerators, water pumps and other appliances can be used by households for entrepreneurial activities (IEA 2024; IEA, IRENA, UNSD, World Bank, and WHO 2024). Shifting from manual labour to machine‐assisted processing can have benefits in terms of productivity and reducing weekly drudgery (ESMAP 2022). Clean and sustainable energy access, particularly through off‐grid systems, can also support a variety of SDGs beyond economic development, such as nutrition, education, health and women's empowerment. Improving access to clean energy can empower communities by decreasing their dependence on traditional fuels, reducing time spent on fuel collection, particularly for women, and providing better education and healthcare opportunities (IEA 2024; IEA, IRENA, UNSD, World Bank, and WHO 2024; IEA 2023).

Over the past decade, financing options for off‐grid sustainable energy have grown, yet it remains insufficient for achieving SDG7 by 2030. IRENA and CPI (2023) suggest that although over USD 3 billion of investments was secured in the off‐grid sector between 2010 and 2021, in 2021, there was still a shortfall of USD 1.8 billion needed to meet energy goals. Between 2010 and 2021, solar photovoltaic (PV) products dominated off‐grid renewable energy investments, accounting for 92% of total investments, with 78% of financial flows coming from high‐income countries to LMICs. However, there were significant regional disparities. SSA received 71% of investments during this period, while South East Asia experienced a 98% decline in investments during the COVID‐19 pandemic years. Furthermore, very few investments have been directed towards small island developing states in the Pacific (IRENA and CPI 2023). Insufficient financing also means that the achievement of SDG7 indicator a.1, which seeks to ensure international financial flows to LMICs, is off‐track and likely to be missed (SEforALL 2024). To meet SDG7, investments not only need to increase but should also be informed by a better understanding of the benefits attributed to off‐grid systems. Without a clear understanding of whether off‐grid technologies can bring about positive outcomes for individuals and communities and with limited funding, knowing what works can help steer funding towards impactful programmes.

### 2.2 The Intervention

Our review explores the evidence on programmatic interventions aimed to increase uptake and use of off‐grid technologies in LMICs. Derived from our previously completed evidence gap map (EGM; Gonzalez Parrao et al. 2024), which summarised the evidence base of rigorous impact evaluations of interventions across SDG7, this review focuses on the promotion of off‐grid technologies and how they affect energy access, climate and socio‐economic outcomes. Considering the scope of such strategies, we adopted a broad and encompassing approach that allowed us to include a variety of technologies spanning tiers 1–5 of the energy technology framework (Bhatia and Angelou 2015).

We define ‘off‐grid interventions’ as any programmes or policies aimed at providing access to new and/or improved off‐grid technologies. These technologies are not connected to the centralised grid and include both stand‐alone systems and mini‐grids (IRENA 2018). These systems often require fewer system‐wide resources than grid expansion and allow for easier access to electricity services in remote areas.

Our review included any intervention that aimed to promote the use and uptake of off‐grid technologies. Based on the description of the interventions included in the review, some of which are provided as examples below, we identified four mechanisms: direct provision, opportunity to access/market expansion, credit and subsidies, and information.

#### 2.2.1 Direct Provision

Direct provision encompasses interventions where a technology is provided to an individual, household or community free of charge. There is no limitation on the scale of technologies that may be provided. For instance, this mechanism may be utilised as part of smaller‐scale programmes where individuals or households receive individual technologies, such as solar lamps/lanterns (e.g., Kelly 2018; Nyakato et al. 2018) or may be used as part of regional and national programmes where technologies are provided to serve a community as a whole, such as solar mini‐grids (e.g., Bensch et al. 2012; Koima 2024). This category covers the free provision of the technology, but not necessarily the financial provision for running said technology.

#### 2.2.2 Opportunity to Access/Market Expansion

As a variation of directly providing technologies to populations, this mechanism encompasses interventions where technologies are brought to new areas/markets. These programmes offer the chance to access new technologies which would not be available in the absence of the intervention. This includes offering populations the opportunity to access new technologies conditional on payment (e.g., Aklin et al. 2017), as well as programmes which introduce technologies to new markets with no further uptake mechanisms (e.g., IDInsight 2018).

#### 2.2.3 Credit and Subsidies

This mechanism encompasses interventions which aim to promote the use and uptake of off‐grid technologies by reducing the financial hurdle populations may face in accessing said technologies. Credit includes interventions where participants are provided with a loan or other repayable amount to access a technology (e.g., Wagner et al. 2021), or when participants rent a technology (e.g., Bensch et al. 2015). Subsidies cover interventions where participants are offered the opportunity to purchase technologies at prices lower than market value (e.g., Aevarsdottir et al. 2017).

#### 2.2.4 Information

The last mechanism involves promoting the use and uptake of off‐grid technologies by providing information to either off‐grid technology suppliers or consumers. Suppliers may be provided with information on how to market products and increase business sales, often through training programmes (e.g., Bensch et al. 2021). On the consumer side, marketing programmes and information awareness campaigns can highlight the benefits of off‐grid technologies and encourage their uptake (e.g., Urpelainen and Yoon 2017).

### 2.3 How the Intervention Might Work

**Figure 1:**
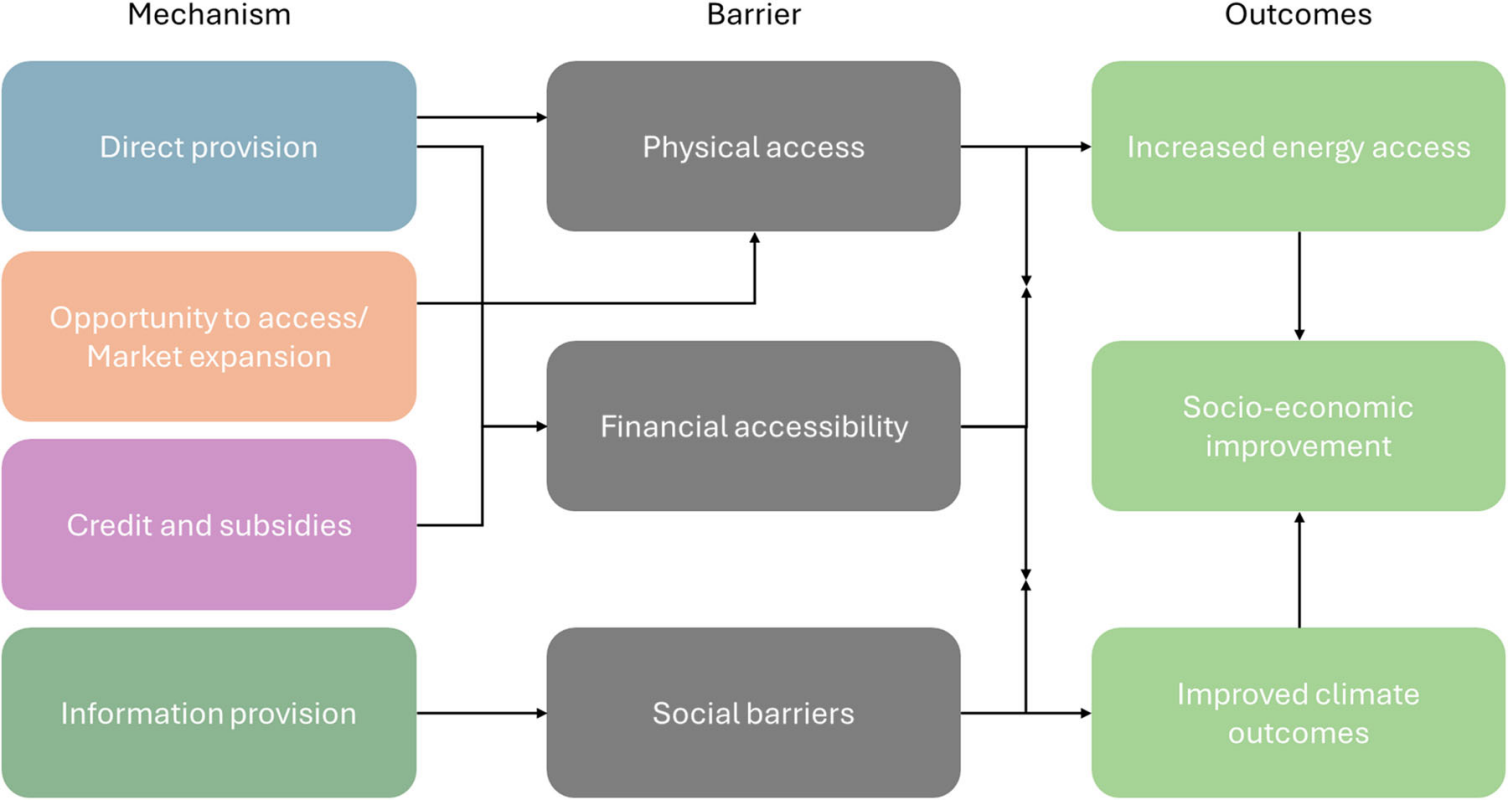
Conceptual framework of interventions aimed to enhance off‐grid electrification rates.

The conceptual framework for this review is based on the framework outlined within our EGM for SDG7 as a whole. Our review covers a subset of interventions included within our EGM. Here, we focus specifically on how the mechanisms included in our review can overcome barriers to the adoption and uptake of off‐grid technologies, and if the adoption of these technologies can lead to a range of outcomes of interest (Figure 1). These barriers and mechanisms were derived from the studies included in this review and, hence, do not necessarily represent the entire universe of potential barriers to accessing and using off‐grid energy. There are also other ways in which these barriers may be overcome, but for this review, we focus on how the four mechanisms described in the previous section aim to overcome each barrier. We note that these pathways rest on a number of assumptions, implying that interventions are implemented and act as intended. For instance, the provision of an off‐grid technology leads to perfect uptake and use.

Off‐grid technologies are implemented as solutions to help achieve rural electrification, a challenge for many reasons. One main reason is accessibility, both in terms of physical and financial access, and social barriers. Physical access and the high costs of connecting rural areas to centralised grid systems are the main reasons cited for the need to move towards off‐grid technologies (Omole et al. 2024; Ortega‐Arriaga et al. 2021; Zomers 2014). When off‐grid technologies are available to households, high‐upfront costs act as a further barrier to their use and uptake (Agoundedemba et al. 2023; Gribkova and Milshina 2022; Singh and Ru 2022; Liao et al. 2021) while social barriers may lead to a lack of acceptance and a lack of awareness of new technologies (Come Zebra et al. 2021). Given these challenges, we outline below how our included interventions seek to overcome each barrier.

#### 2.3.1 Overcoming Barriers

The *direct provision* of technologies aims to overcome two of our identified barriers, *physical access* and *financial accessibility*. Given the high costs of expanding centralised grid networks to rural areas, off‐grid technologies have the benefit of being standalone systems that can produce electricity. Specifically, the establishment of mini‐grids can help serve communities, including households and businesses (Kishore et al. 2012). Alternatively, other technologies, such as SHS, are available in multiple sizes, all of which aim to serve a household's energy needs (Bond et al. 2012). Given that these technologies are directly provided to households through this mechanism, they completely bypass the financial barriers to ownership. In the same way that the direct provision of technologies overcomes physical access issues, *opportunity to access/market expansion* interventions also overcome *physical access* issues by setting up off‐grid technologies in new markets.

*Credit and subsidies* aim to overcome *financial hurdles* that may prevent either households or communities from investing in off‐grid technologies, or businesses from expanding their off‐grid capacity. Subsidies are a common mechanism to help promote off‐grid technology ownership at the household level (Kizilcec and Parikh 2020), while community technologies, such as mini‐grids, face high startup costs, and so financial support can be required to install these technologies (Shahzad et al. 2023; Peters et al. 2019).

Once off‐grid technologies are available and financially accessible to communities, there may still be social barriers that prevent households from accessing said technologies. Social acceptance of new technologies may be limited when there is no clear communication and information on the benefits as well as the limitations of new technologies. If communities gain a negative perception or false expectation of new technologies, they may be less willing to engage with programmes (Feron 2016; Urmee and Md 2016; Ahlborg and Hammar 2014). *Information provision* seeks to overcome this barrier by ensuring that the benefits from off‐grid technologies are made available to intervention communities to encourage households to move away from traditional energy sources.

#### 2.3.2 Pathways to Final Outcomes

Once barriers to the uptake and use of off‐grid technologies are overcome, we defined two pathways to improving socio‐economic outcomes. First, with an increase in ownership of off‐grid technologies comes an increase in energy access. There are different ways in which an increase in energy access can lead to improved socio‐economic outcomes. For instance, greater access to light means that students can study for longer, which can lead to improved education outcomes (Banerjee et al. 2021).

The second pathway involves improved climate outcomes through the reduction of traditional energy sources. Once households utilise off‐grid technologies, they may move away from traditional energy sources. Traditional energy sources such as biomass and kerosene can increase the level of household pollutants, including greenhouse gases (GHGs; Lam et al. 2012), leading to negative health outcomes for households (Epstein et al. 2013; Kim et al. 2011).

### 2.4 Why It Is Important to Do This Review

Evidence on the effects of interventions aimed at promoting off‐grid technologies has grown in the past 4 years. Within our EGM, 40% of studies from the *off‐grid systems* intervention category were published since 2020 (20 studies).

Our EGM also identified four systematic reviews published since 2020 within the *off‐grid systems* category. Huang et al. (2022) solely focused on the impact pathways of the Photovoltaic Poverty Alleviation (PVPA) programme in China. They found the programme had the potential to alleviate poverty through income distribution but mentioned the need for further evaluation of its sustainability and initial implementation issues. Cissé (2022) sought to understand whether experimental or quasi‐experimental evaluation methods were more likely to produce positive outcomes, and used the valuation of solar nano projects in developing countries as their focus. The author found that both methods are broadly equal in success, though quasi‐experimental methods may be less likely to identify negative outcomes. Khogali et al. (2022) looked at the impact of the electrification of health systems in LMICs. They found that in the absence of interventions, unavailable and unreliable electricity is a bottleneck to health care provision, while the electrification of health services helped overcome this barrier to provide a reliable service. Finally, Moore et al. (2020) conducted a review of the impact of access to electricity interventions in LMICs utilising a broad scope, which included both on‐grid and off‐grid interventions. The authors found modest positive effects on education, socioeconomic welfare and health outcomes.

The EGM also showed that these four reviews were assessed as low confidence, meaning they did not meet the minimum standards associated with conducting and reporting systematic reviews. While the findings and insights from the abovementioned reviews are valuable, given the growth of the literature in the past 4 years and the narrow scope of interventions within these reviews, there is a need to conduct a broader, up‐to‐date and high confidence review.

We envisage at least three opportunities to use this review. First, it can be used by policymakers to inform the improved design of off‐grid energy interventions, informed by a rigorous synthesis of the evidence base of the impacts of off‐grid technology interventions on energy access, climate and socio‐economic outcomes. Second, it can inform investment decisions to help ensure that funding is directed towards interventions that have proven effects. Third, this review can identify gaps in the research on outcomes of high interest, for which there is currently not enough available evidence to draw conclusions about the impact effects of off‐grid technology interventions.

## 3 Objectives

Drawing from the EGM, we conducted an iterative process to identify priority areas that could be addressed with the available evidence and ultimately selected the research questions for the review. More details of this process can be found in Appendix B. The purpose of this systematic review is to identify, assess and synthesise evidence of the effects of interventions promoting the uptake and use of off‐grid technologies in LMICs. To achieve this objective, we answer the following research questions:

1. Which off‐grid electrification interventions are effective at supporting energy access, climate and socio‐economic development outcomes?
2. How do these effects vary by region, population and other intervention characteristics?
3. What are the major challenges and facilitators to achieving effects in off‐grid electricity access interventions?

Ultimately, we hope that the recommendations put forward in our review can be used to influence the design and implementation of off‐grid programming, leading to greater energy access, climate and socio‐economic outcomes.

## 4 Methods

### 4.1 Criteria for Considering Studies for This Review

Given that our review is derived from our EGM, the majority of our search and screening occurred at the EGM stage. The section below outlines how we utilised the EGM for selecting studies for this review. A full explanation of the methods used in this review is available in our protocol (Gonzalez Parrao et al. 2023).

#### 4.1.1 Types of Studies

To address research questions 1 and 2, we included impact evaluations that use an experimental or QED to robustly measure a change in outcomes that is attributed to an intervention as compared to an appropriate counterfactual. We included the following study designs, which are widely used to evaluate intervention effectiveness (Aloe et al. 2017; Reeves et al. 2017; further details of the included study designs are available in Appendix A.1):

1. Randomised controlled trials (RCTs)
2. Natural experiments with clearly defined intervention and comparison groups
3. Regression discontinuity designs
4. Instrumental variables
5. Endogenous treatment‐effects models, endogenous switching regression and other methods synonymous with the Heckman two‐step model
6. Difference‐in‐differences, two‐way fixed‐effects and two‐way Mundlak regressions
7. Interrupted time series models
8. Weighting and matching approaches which control for observable confounding
9. Synthetic control methods

To address research question 3, we searched for additional documentation on the programmes identified for RQs 1–2. We included a range of evidence, such as primary qualitative studies, mixed‐methods studies and process evaluations.

#### 4.1.2 Types of Participants

We included studies on the effects of interventions on participants residing in LMICs as defined by the World Bank's country and lending groups (a full breakdown of country classifications is available in Appendix A.2). Studies were classified according to their income status during the first year that an intervention began. Studies that included evidence from multiple countries were included so long as results were provided for at least one LMIC.

Aside from exclusion based on the income status of a country, no further restrictions were applied based on the type of participants.

#### 4.1.3 Types of Interventions

We included interventions that aimed to promote the uptake and use of off‐grid technologies. Appendix B provides details on how these interventions were selected from our previous EGM. In brief, after consultations with the review co‐funder, Sustainable Energy for All (SEforALL), we agreed to focus on off‐grid technologies. As our EGM included interventions on off‐grid technologies across different intervention categories, so long as the intervention aimed to increase the uptake and use of one of our included off‐grid technologies, it was eligible for inclusion within our review (Table 1).

**Table 1:** Included off‐grid technologies.

Technology	Definition
Engine	An engine which can be used for any function, so long as it is powered through an off‐grid energy source. This could for instance be solar, or any other sustainable source.
General solar products	When papers are not explicit on the solar technologies provided but it is clear that they encompass at least one of the technologies within this table, this code was used.
Micro‐hydro	Any technology related to the production of energy through hydro systems, including micro‐hydro plants which can produce energy for communities.
Solar home system (SHS)	A household system that consists of solar panels connected to a range of lights as well as charging points.
Solar improved cookstoves (ICS)	Improved cookstoves which are safer and more energy efficient than traditional systems. Only cookstoves with an added solar feature, such as a solar‐assisted fan, are included here.
Solar irrigation	Irrigation systems which run on solar energy. These can range from household to larger agricultural applications.
Solar lamp/lantern	A light ran on solar energy through the use of a use a solar panel. This category captures a single light which is not connected to any broader system.
Solar lamp/lantern with mobile phone charger	The same as the above category with the addition of a charger which can be used to charge a mobile phone.
Solar market garden (SMG)	Solar market gardens combine solar‐powered pumps with drip irrigation systems. The pump is what differentiates this technology from the irrigation category.
Solar mini‐grid or microgrid	Microgrid systems which generate energy through the use of solar. Systems serve communities and generate power for multiple households and businesses.
Solar oven	A cooking pot which utilises solar energy to perform the cooking function. This is coded separately from solar improved cookstoves as there is no emphasis on the energy efficiency or safety of these technologies.
Solar panels (not part of a wider system)	Solar panels without connection and provision of additional technologies such as lamps and charging stations.

These technologies, which vary in their use, may be provided as part of programmes for differing purposes. For example, a solar oven may aim to increase health outcomes through a reduction in the use of traditional cookstoves, while solar lighting might hope to increase educational outcomes through an increase in time studying. Despite these varying aims, so long as the technology provided constituted an off‐grid technology and the study evaluated one of our eligible outcomes (alongside our other eligibility criteria), it was included in the review.

As the focus of the review is on the promotion of uptake and use of off‐grid technologies, some studies from our EGM that focused on renewable energy were not eligible. For instance, we did not include studies that aimed to increase the performance and efficiency of the renewable energy sector regarding the generation of energy (including solar) and the creation of renewable energy technologies (Wu et al. 2023; Luan and Lin 2022). While the development of renewable energy technologies is a prerequisite for the promotion of said technologies, development subsidies are not focused on increasing the use of technologies.

For the purpose of our review, an ‘intervention’ is defined as a decision or set of activities deliberately undertaken by a specific entity—such as a government body, NGO, private firm or consortium—intended to influence events or outcomes, which another such entity could emulate. Studies where the independent variable is not an intervention but a state of affairs were excluded. For instance, a household's access to off‐grid electricity may be influenced by an intervention, but when the variation in the independent variable has arisen as a result of uncoordinated activity (i.e., some people purchased off‐grid technologies and some people did not), we did not classify this as the result of an implementable intervention. This is true even when a counterfactual study design is used to identify the causal impact of the independent variable, rather than mere associations.

#### 4.1.4 Types of Outcome Measures

We included studies that evaluated energy access, climate and socio‐economic development outcomes. The exact outcomes for our review were decided upon after the agreement that our review would focus on off‐grid technologies. A full breakdown of this process is outlined in Appendix B. After identifying eligible papers from our EGM based on their intervention, we then mapped the outcomes reported in these studies. From here, and in consultation with SEforALL, we agreed to prioritise 18 includable outcomes in our review (Table 2, see Appendix B for further details on this process).

**Table 2:** Outcomes included in our review.

Group	Outcome	Definition
Energy Access	Energy security	Measures of energy security as the uninterrupted availability of energy sources at an affordable price. This includes measures of energy poverty and individual indicators of energy access, quality and reliability.
	Lighting hours	The number of hours per day a household or individual uses lighting.
	Kerosene use	Measures of the use of kerosene, either through recall of kerosene consumption or kerosene expenditure.
	Energy expenditure	The amount households or individuals spend on energy (excluding kerosene). This can either be aggregated or disaggregated based on energy source.
	Technology usage	Measures of the usage of an off‐grid technology after it has been received or bought. This includes usage measures of alternative technologies, for example, those that the intervention aimed to replace.
	Technology uptake or upgrade	Measures of the uptake of an off‐grid technology, including the uptake of a new technology or the replacement/upgrade of an old technology. This excludes measures related to how the technology is used.
Climate	Air quality	Measures of air pollution that would have been emitted if more energy had been consumed in the absence of the intervention. These emissions, usually of SOX, NOX, black carbon or particulate matter, can be from the combustion of heating fuels, such as natural gas and fuel oil.
	GHG emissions	Measures of carbon related emissions (CO_2_) and non‐carbon related emissions, for example, methane (CH_4_), nitrous oxide (N_2_O) and fluorinated gases.
Socio‐economic	Income	Measures of a household or individual's income, either overall or from disaggregated sources.
	Household/domestic activities	Measures of hours spent on household/domestic activities as an overall measure or disaggregated by task. Examples of household/domestic activities include cooking or collecting fuel.
	Leisure activities	Measures of hours spent on leisure activities as an overall measure or disaggregated by task. Examples of leisure activities include listening to the radio or watching television.
	Resting hours	The number of hours per day an individual rests, that is, not active (awake). Measures of active hours are also captured under this outcome.
	Employment	Measures of employment, including, for example, hours worked or new jobs created.
	Study hours	The number of hours a student spends studying. This can be captured in school or at home.
	School attendance	The number of days a student spends attending school. Measures of absenteeism are also eligible under this outcome.
	Test scores	Measures of test scores, including overall scores or subject disaggregated scores.
	Respiratory illness	Measures of respiratory illness, including indices and individual indicators.
	Women's empowerment	Measures of women's empowerment, including indices and individual indicators of, for example, decision‐making.

All included outcomes were deemed as primary outcomes. Energy access outcomes cover measures of a household's access and utilisation of energy. This includes whether there is a secure energy service, how much energy is used and measures of whether there is access to energy through the use and uptake of off‐grid technologies.

From our EGM, we knew that few studies focusing on off‐grid technologies evaluated climate outcomes. Only air quality and GHG emissions were included under this category.

The EGM covered a broad range of socio‐economic development outcomes. For this review, we focused on 10 outcomes. We included one economic measure, income; five measures of time allocation: household/domestic activities, leisure activities, active hours, employment and study hours; two education measures: school attendance and test scores; and one measure of health and women's empowerment.

#### 4.1.5 Duration of Follow‐Up

We placed no restrictions on the duration of follow‐up within a study.

#### 4.1.6 Types of Settings

Aside from the restrictions on intervention participants having to reside in LMICs, no other eligibility requirement was placed on settings.

#### 4.1.7 Additional Criteria

We included studies in any language, although search terms were developed in English. Both academic and grey literature studies were included. Only studies published since 2000 were included in our review. From 3ie's experience developing other EGMs, less than one per cent of impact evaluations implemented in LMICs predate the Year 2000, hence the likelihood of missing eligible studies is small.

### 4.2 Search Methods for Identification of Studies

In the next sections, we outline the search process used to identify literature for our original EGM. We conducted a comprehensive search for relevant studies in both academic and grey literature sources.

#### 4.2.1 Electronic Searches

We adopted a systematic search strategy following guidelines for systematic literature searching (Kugley et al. 2017). The search strategy was developed in collaboration with the information specialist Alison Bethel. The search was designed to address potential publication bias issues by systematically searching academic bibliographic databases and implementing additional searches for grey literature in specialist organisational websites, websites of bilateral and multilateral agencies and repositories of research in international development. The full list of literature sources searched is available in Table 3, and an example of the search strings used for one database is presented in Appendix C. The precise strings and logic (e.g., index terms and truncation operators) were adapted for each database and platform.

**Table 3:** Academic and grey literature sources.

Academic databases	
Applied Social Sciences Index and Abstracts (ASSIA)	
CAB Abstracts	
EBSCO Discovery (RePEc, World Bank eLibrary, Oxfam Policy and Practice)	
Econlit (via EBSCOhost)	
EBSCO: Gender Studies, GreenFILE, Africa‐wide	
Environment Complete	
Epistemonikos	
Global Health	
IBSS	
Medline	
Latin American and Caribbean Health Science (LILACS)	
ProQuest Dissertations and Theses Global	
SciELO (Scientific Electronic Library Online)	
Scopus	
Web of Science: Science Citation Index (1990)	
Web of Science: Social Science Citation Index (1990)	
Web of Science: Arts and Humanities Citation Index (1975–)	
Web of Science: Emerging Sources Citation Index (2015–)	

Grey Literature	
Source	URL
Abdul Latif Jameel Poverty Action Lab (J‐PAL)	https://www.povertyactionlab.org/
African Development Bank (ADB)	https://www.afdb.org/en
British Library for Development Studies	https://bldslegacycollection.uk/
Campbell Collaboration Evidence Portal	https://www.campbellcollaboration.org/
Collaboration for Environmental Evidence Database of Evidence Reviews (CEEDER)	https://environmentalevidence.org/ceeder/
Centre for Global Development	https://www.cgdev.org/
Centre for Effective Global Action (CEGA)	https://cega.berkeley.edu/
Cochrane Database of Systematic Reviews	https://www.cochranelibrary.com/
Deutsche Gesellschaft für Internationale Zusammenarbeit	https://www.giz.de/en/html/index.html
Energy Policy Institute at the University of Chicago (EPIC)	https://epic.uchicago.edu/
EU Publications	https://op.europa.eu/en/web/general-publications
European Commission	https://commission.europa.eu/index_en
Foreign, Commonwealth and Development Office (FCDO)	https://www.gov.uk/government/organisations/foreign-commonwealth-development-office
German Institute for Development Evaluation (DEval)	https://www.deval.org/en/
DEval Rigorous Impact Evaluation	https://rie.deval.org/
Innovations for Poverty Action (IPA)	https://poverty-action.org/
Inter‐American Development Bank (IDB)	https://www.iadb.org/en
International Initiative for Impact Evaluation (3ie) Development Evidence Portal	https://developmentevidence.3ieimpact.org/
International Monetary Fund	https://www.imf.org/en/Home
National Bureau of Economic Research (NBER) – Working Papers	https://www.nber.org/
Overseas Development Institute	https://odi.org/en/
OECD iLibrary	https://www.oecd-ilibrary.org/
Oxfam	https://www.oxfam.org/en
Registry for International Development Impact Evaluations	https://ridie.3ieimpact.org/
Social Science Research Network	https://www.ssrn.com/index.cfm/en/
Swedish International Development Cooperation Agency (SIDA)	https://www.sida.se/en
UN DAC Resource Centre	https://undac.unocha.org/about/
United Nations Evaluation Group	https://www.uneval.org/
USAID Development Experience Clearinghouse (DEC)	https://dec.usaid.gov/dec/home/Default.aspx

In creating our search terms, we utilised pearl‐harvesting techniques as suggested by Sandieson (2006). This technique involved identifying a small sample of known studies relevant to the review. These studies were identified from previous 3ie work in the energy sector (Berretta et al. 2021; Moore et al. 2020) and from 3ie's Development Evidence Portal (DEP). Once these initial studies were identified, as search terms were developed and tested, we ensured that our sample of studies was captured in relevant databases.

#### 4.2.2 Searching Other Resources

To minimise the likelihood of missing studies, we conducted forward and backward citation tracking of studies included in the academic and grey literature search. Backwards citation tracking included checking the reference lists of all included studies for other potentially relevant papers. Forwards citation tracking, conducted through Google Scholar, included identifying studies which had cited our included papers and checking these for relevance. We also published a blog^1^ and contacted key researchers and organisations for additional studies.

To address research question 3, we conducted an additional search for supporting documentation on the programmes identified for research questions 1 and 2. This search was conducted in two steps. Firstly, in the same manner as outlined above, we conducted both backward and forward citation tracking of our included studies. We supplemented this by searching Google using relevant terms related to our included programmes, such as the programme name or specific technologies that may have been deployed.

### 4.3 Data Collection and Analysis

#### 4.3.1 Description of Methods Used in Primary Research

Based on the eligibility requirements set out above, we anticipated including studies with an appropriate counterfactual, based on an experimental or QED.

#### 4.3.2 Selection of Studies

Our EGM report (Gonzalez Parrao et al. 2024) outlines the full methods used to screen studies. In brief, we began by importing the search results into EPPI‐Reviewer (Thomas et al. 2022), the software we used to manage our records. We then removed duplicate records. Once we had completed backwards and forward citation tracking, these records were also imported and de‐duplicated.

After de‐duplication, we screened records based on their title and abstract. We trained a set of reviewers on batches of records until we reached an 80% inter‐coder reliability rating. Once we had reached this mark, reviewers independently screened records based on their title and abstract. At all times, reviewers were able to select an ‘unsure’ code, whereby a member of the core research team would then make an eligibility decision. At this stage, we also utilised EPPI's classifier function, which estimates the probability that a given record will be of relevance based on its title and abstract. We utilised 3ie's DEP classifier model, which has been trained with screening decisions on over 100,000 records. We ultimately excluded any record that scored less than 15% likelihood of inclusion. All other records were screened.

After this stage, we attempted to retrieve the full text of all included records to begin screening based on their full text. Any record for which we could not retrieve the full text was excluded. Two reviewers then independently screened each full text based on our eligibility requirements. In case of disagreements, a third core research team member reviewed the full text and resolved the disagreement.

The above steps outline the process used to select studies for our EGM. For this review, we took further steps to ensure the relevance of the studies. After discussions with SEforALL to define the scope of this review, one core team member listed the relevant studies from the EGM. All studies that did not focus on an off‐grid technology and report an outcome of interest were excluded. This stage required no further screening and utilised the data that had been extracted during the EGM.

#### 4.3.3 Data Extraction and Management

The majority of the study characteristics data had already been extracted as part of our EGM. For the data extracted as part of the EGM, trained reviewers extracted data with a second core research team member checking a random subset of this data. Appendix D provides the full data extraction forms. For the extraction of quantitative data to calculate effect sizes, two reviewers extracted all data, and any disagreements were reconciled with a core research team member.

Once all effect sizes were calculated and converted to a standardised mean difference (SMD) (as described in detail below), we examined the data for outliers. We defined outliers as any effect sizes ± 3.29 standard deviations from the mean, following the guidance of Tabachnick and Fidell (2001). Sensitivity to outliers was examined as discussed in the sensitivity analysis section below.

#### 4.3.4 Assessment of Risk of Bias in Included Studies

We assessed the risk of bias of included studies using 3ie's risk of bias tool (see Appendix E). This tool covers both internal validity and statistical conclusion validity of experimental and quasi‐experimental impact evaluations (Hombrados and Waddington 2012). Two reviewers undertook the risk of bias assessment, and any disagreements were reconciled through discussion with a core research team member. We conducted the risk of bias assessment for each estimate we extracted, reflecting that estimates of different outcomes within the same study may score differently in the assessment. After assessing each bias dimension, a study was coded as ‘low risk of bias’ if all dimensions were assessed as low; as ‘high risk of bias’ if *any* of the dimensions were scored as high; and all other estimates were categorised as ‘some concerns’.

#### 4.3.5 Measures of Treatment Effect

An effect size expresses the magnitude (or strength) and direction of the relationship of interest (Valentine et al. 2015; Borenstein et al. 2009). We extracted data from each study to calculate standardised effect sizes for cross‐study comparison wherever possible. For continuous outcomes comparing group means in a treatment and control group, we calculated the SMDs, or Cohen's *d*, its variance and standard error using formulae provided in Borenstein et al. (2009). An SMD is a difference in means between the treatment and control groups divided by the pooled standard deviation of the outcome measure. Cohen's *d* can be biased in cases where sample sizes are small. Therefore, in all cases, we simply adjusted Cohen's *d* using Hedges' method.

As primary studies have become increasingly complex, it has become commonplace for authors to extract partial effect sizes (e.g., a regression coefficient adjusted for covariates) in the context of meta‐analysis. For studies reporting regression results, we followed the approach suggested by Keef and Roberts (2004) using the regression coefficient and the pooled standard deviation of the outcome. When the pooled standard deviation of the outcome was unavailable, we used regression coefficients and standard errors or *t*‐statistics.

Members of the core research team reviewed all estimates and sample sizes extracted to ensure that the correct formulae were employed in effect size calculations. After synthesis, we converted pooled effect sizes to commonly used metrics such as percentage changes and mean differences in outcome metrics typically used (e.g., weight in kg), whenever feasible.

To our knowledge, effect benchmarks have not yet been developed for off‐grid energy programmes. Following the Grading of Recommendations Assessment, Development, and Evaluation guidelines (GRADE; Guyatt et al. 2008), we interpreted the magnitude of effect sizes against the benchmarks listed in Table 4.

**Table 4:** Standardised mean difference benchmarks from GRADE.

Magnitude of the effect, in standard deviations (SD)	Interpretation
Effect size < 0.10	Very small effect
Effect size ≥ 0.10 and < 0.20	Small effect
Effect size ≥ 0.20 and < 0.37	Moderate effect
Effect size ≥ 0.37	Large effect

#### 4.3.6 Unit of Analysis Issue

Unit of analysis errors can arise when the unit of allocation of treatment is different to the unit of analysis of the effect size estimate, and this is not accounted for in the analysis (e.g., by clustering standard errors at the level of allocation). We assessed studies for unit of analysis errors (The Campbell Collaboration 2019), and when they existed, we corrected them by adjusting the standard errors according to the following formula (Higgins et al. 2020; Waddington et al. 2012; Hedges 2009):

$$\mathrm{SE}(d)\prime =\mathrm{SE}(d)\times \sqrt{1+(m–1)\;c},$$

where *m* is the average number of observations per cluster and *c* is the intra‐cluster correlation coefficient. When included studies used robust Huber‐White standard errors to correct for clustering, we calculated the standard error of *d* by dividing *d* by the *t*‐statistic on the coefficient of interest.

#### 4.3.7 Criteria for Determination of Independent Findings

Complex data structures are a common occurrence in meta‐analyses of impact evaluations. There are several scenarios through which these complex structures with dependent effect sizes might occur. For instance, there could be several publications that stem from one study, or several studies based on the same data set. Some studies might have multiple treatment arms that are all compared to a single control group. Other studies may report outcome measurements from several time points or use multiple outcome measures to assess related outcome constructs. All such cases yield a set of statistically dependent effect size estimates (Borenstein et al. 2009).

The research team assessed the extent to which relationships exist across the studies included in the review. When we had several publications reporting on the exact same effect, we used effect sizes from the most recent publication. We utilised information provided in studies to support these assessments, such as sample sizes, programme characteristics and key implementing and/or funding partners.

We extracted effects reported across different outcomes or subgroups within a study, and where information is collected on the same programmes for different outcomes at the same or different periods of time, we extracted information on the full range of outcomes over time. Where studies reported effects from multiple model specifications, we used the author's preferred model specification. If this was not stated or was unclear, we used the effect with the greatest number of controls. Where studies reported multiple outcome sub‐groups for the same outcome construct, we calculated a ‘synthetic effect size’ (Borenstein et al. 2009). Where studies reported multiple outcomes or evidence according to sub‐groups of participants, we recorded and reported data on relevant sub‐groups separately.

#### 4.3.8 Dealing With Missing Data

In cases of relevant missing or incomplete data in studies identified for inclusion, we made every effort to contact study authors to obtain the required information. When we were unable to obtain the necessary data, we reported the characteristics of the study stating that it could not be included in the meta‐analysis or reporting of effect sizes due to missing data.

#### 4.3.9 Assessment of Heterogeneity

We assessed heterogeneity by calculating the *Q*‐statistic, *I* ^2^ and Tau^2^ to provide an estimate of the amount of variability in the distribution of the true effect sizes (Borenstein et al. 2009). We complement this with an assessment of the heterogeneity of effect sizes graphically using forest plots.

#### 4.3.10 Assessment of Reporting Biases

To reduce the possibility of publication bias, we searched for and included unpublished studies in the review. We also tested for the presence of publication bias through the use of contour‐enhanced funnel graphs (Peters et al. 2008) and statistical tests (Egger et al. 1997) for outcomes for which we identified at least 10 studies.

#### 4.3.11 Data Synthesis

We conducted meta‐analyses of studies that we assessed to be sufficiently similar. We combined studies using meta‐analysis when we identified two or more effect sizes using a similar outcome construct and where the comparison group state was judged to be similar across the two, in line with the approach taken by Wilson et al. (2011). We combined studies in the same analysis when they evaluated the same outcome type. Moderator analyses took into account multiple interventions as moderator variables, allowing us to also examine the impact of different intervention types by outcome. Where there were too few studies, or included studies were considered too heterogeneous in terms of interventions or outcomes, we present a discussion of individual effect sizes along the causal chain. As heterogeneity exists in theory due to the variety of interventions and contexts included, we used inverse‐variance weighted, random effects meta‐analytic models (Higgins et al. 2020). We used the metafor package (Viechtbauer 2010) in R software to conduct the meta‐analyses (R Core Team 2020). To facilitate the interpretation of effect sizes in the discussion section, we converted statistically significant findings from the meta‐analysis (Cohen's *d*) into probabilities of superiority. This indicates the probability that a participant picked at random from the treatment group will score higher in the outcome measure compared to a random participant from the control group (Magnusson 2023).

To enrich our findings and to be able to draw out policy recommendations, we integrated our quantitative and qualitative findings. This process involved identifying where our findings from each synthesis overlapped and where they contrasted. This was completed in a qualitative manner with no additional quantitative analysis being conducted based on these overlaps.

#### 4.3.12 Subgroup Analysis and Investigation of Heterogeneity

When feasible, we conducted moderator analyses to investigate sources of heterogeneity. Following the PROGRESS‐PLUS approach (Oliver et al. 2017), we assessed moderators falling into three broad categories of extrinsic, methodological and substantive characteristics to address inequity aspects within the off‐grid technology context. The final list of moderators was decided in consultation with SEforALL and includes:

- Extrinsic characteristics: publication date.
- Methodological characteristics: risk of bias, study design.
- Substantive characteristics: participant characteristics (gender, age, socio‐economic status, education, land ownership), context (region, country), intervention type (technology, mechanism), intervention features (target actor, implementation scale, supply/demand focus, implementation agency).

We used random effects meta‐regression to investigate the association between moderator variables and heterogeneity of treatment effects (Borenstein et al. 2009) and sub‐group analyses to investigate heterogeneity by treatment sub‐groups (e.g., men and women, poor and non‐poor, and so on). If the latter strategies were not possible (i.e., if we did not have a sufficient number of studies or data), we discussed and explored the factors which may be driving heterogeneity of results narratively by conducting cross‐case comparisons (Miles and Huberman 1994).

#### 4.3.13 Sensitivity Analysis

We conducted a sensitivity analysis to assess whether the results of the meta‐analysis are sensitive to the removal of any single study. We did this by removing studies from the meta‐analysis one by one and assessing changes in results. We also assessed the sensitivity of results to the inclusion of high‐risk of bias studies by removing these studies from the meta‐analysis and comparing results to the main meta‐analysis results. Finally, we assessed sensitivity to outliers by comparing results with and without outliers included.

#### 4.3.14 Treatment of Qualitative Research

We used qualitative research to supplement the findings of the interventions covered by the included studies. We did not seek out all qualitative studies relating to off‐grid technologies in LMICs, but under the ‘effectiveness +’ framework (Snilstveit 2012), we searched for qualitative studies to provide additional information about the context and implementation of relevant interventions. This included primary qualitative studies, mixed‐methods studies and process evaluations.

One coder independently appraised these studies and documents based on an adapted version of the Critical Appraisal Skills Programmes checklist utilised in previous 3ie work (Snilstveit 2012). We assessed the quality of qualitative and descriptive quantitative studies by appraising the adequacy of reporting, data collection, presentation, analysis and conclusions drawn. Finally, project documents provide information about the design or resources available for a project. As these documents present factual information about interventions, we did not formally appraise the quality of such documents but instead assessed the relevance of the documents against the interventions included in the review.

#### 4.3.15 Summary of Findings and Assessment of the Certainty of the Evidence

We did not plan to include a summary of findings and an assessment of the certainty of the evidence.

## 5 Results

### 5.1 Description of Studies

#### 5.1.1 Results of the Search

**Figure 2:**
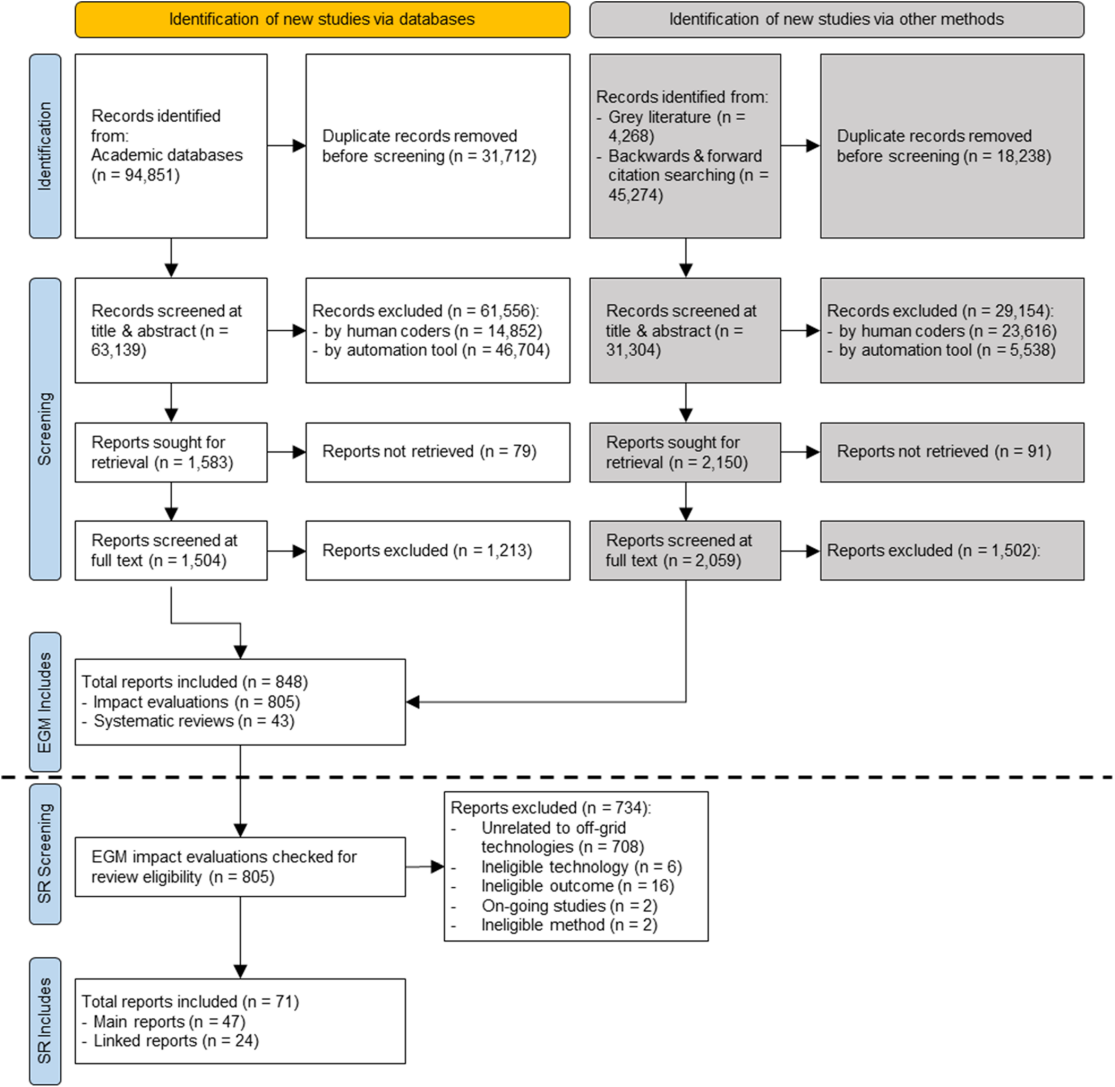
PRISMA flowchart.

The PRISMA diagram (Figure 2) shows the results of our search to identify studies for research questions 1 and 2. From our EGM's initial search ran between June and September 2023, we identified 144,393 records. After removing duplicates, we screened 94,443 records based on their title and abstract. Ultimately, we included 848 records in our EGM (our EGM report provides greater details about why studies were excluded).

From our EGM, we identified 97 records related to the promotion and uptake of off‐grid technologies. From these, six records were excluded as they evaluated a technology outside the scope of our review (our excluded studies section below provides examples of these studies). A further 16 records were excluded as they evaluated an outcome outside of our framework, while 2 records were excluded as they were ongoing evaluations. In both cases, we sought to identify a completed evaluation of these studies, but this was not available in either case. Finally, due to misalignment between our EGM and review, we excluded a further two studies as they did not utilise a method from our inclusion criteria. A systematic review allows for a more thorough exploration of studies, and hence, we identified the methodological issues with these studies at this stage.

Ultimately, we included 71 records. Of these, 47 records were categorised as the ‘main’ study, while 24 were deemed ‘linked’ studies.^2^ From our 47 included records, two programmes were evaluated in multiple papers, this is the PVPA programme in China (Li et al. 2023; Xiao et al. 2023) and the Cooking and Pneumonia Study (CAPS) in Malawi (Nightingale et al. 2019; Kelly 2018). Throughout our review, we present results at the study level and refer to the 47 included studies throughout.

#### 5.1.2 Included Studies

##### 5.1.2.1 Year of Publication

Our 47 included studies were published between 2011 and 2023. Publication trends here support the finding of our EGM that there is a recent expansion in the number of sustainable energy related evaluations, with 77% of our studies being published since 2017.

##### 5.1.2.2 Geography

**Figure 3:**
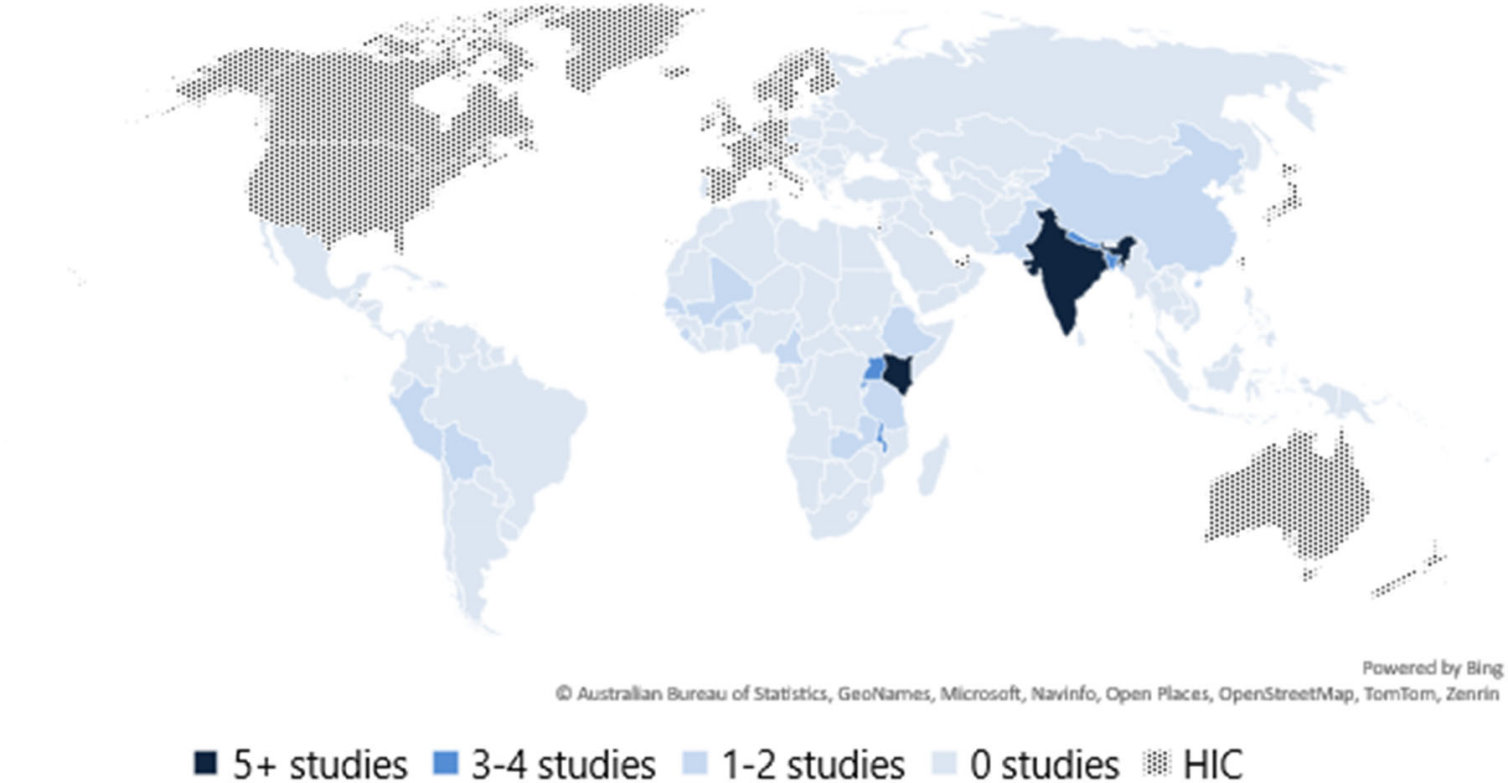
Geographical distribution of studies.

The map in Figure 3 displays the geographical distribution of the 47 studies included in our analysis. Most of these studies evaluated programmes within SSA (*n* = 30) and South Asia (*n* = 13), while only two studies evaluated programmes within Latin America and the Caribbean (LAC) and two in East Asia and the Pacific. Within regions, we also find a concentration of evidence. Within SSA, 23 studies (75%) took place within countries in eastern Africa, while in South Asia, 6 studies (46%) took place in India.

The country with the highest number of studies in our sample is Kenya (*n* = 8), closely followed by India (*n* = 6). We also identified multiple studies in Rwanda and Uganda (*n* = 4), Bangladesh and Nepal (*n* = 3) and Malawi, Senegal and Zambia (*n* = 2). In contrast to our EGM, we only identified two studies from China. Despite identifying 249 impact evaluations from China within our EGM, many of these studies focused on the business performance and generation of renewable energies and hence were excluded.

##### 5.1.2.3 Intervention Mechanism

Among our 47 included studies, the direct provision of technology was the most commonly evaluated mechanism (Table 5). This includes, for example, the direct provision of mini‐grids (e.g., Bahaj et al. 2019), SHS (e.g., Bensch et al. 2012) and improved cookstoves (e.g., Kelly 2018). Financial mechanisms were the second most common, including providing subsidies to reduce the cost of purchasing technologies (e.g., Meriggi et al. 2021), providing financial support towards covering energy costs (e.g., Numminen et al. 2018), as well as renting out technologies (e.g., Arráiz and Calero 2015).

**Table 5:** Number of studies by mechanism.

Mechanism	No. of studies
Direct provision	21
Financial (credit or subsidy)	17
Multi‐mechanism	6
Information provision	5
Opportunity to access/market expansion	5

*Note:* The sum of the studies in the above table is greater than 47, as multiple studies contained multiple treatment arms with different mechanisms. In these cases, the study was counted towards each mechanism.

Far fewer studies focused on our other two mechanisms. Information provision, such as market demonstrations (e.g., Urpelainen and Yoon 2017), were only evaluated in five studies. Opportunity to access/market expansion was also only evaluated in five studies and included, for example, the opportunities for households to access solar microgrids (e.g., Aklin et al. 2017).

Multi‐mechanism was used as a code whenever a programme had a treatment which was a combination of any of the four mechanisms. For instance, Mekonnen et al. (2021) provided a treatment which includes information on the benefits and use of solar lamps, while also providing a subsidy for the lamps at the same time.

##### 5.1.2.4 Intervention Technology

SHS was the most commonly evaluated technology (Table 6), this was most commonly a package of different household solar technologies. For instance, the Yeelen Ba programme in Burkina Faso offered three different packages of SHS providing enough electricity to feed at least two light bulbs for around 4–5 h (Bensch et al. 2015). Solar lamps/lanterns without mobile phone chargers were the second most commonly evaluated technology, though, when combined with the number of programmes focusing on solar lamps/lanterns with mobile phone chargers, the total number of studies rises to 15,^3^ making them the most commonly evaluated technology.

**Table 6:** Number of studies by technology.

Technology	No. of studies
Solar home systems (SHS)	14
Solar lamp/lantern	10
Solar mini‐grid	9
Solar lamp lantern with mobile phone charger	6
Solar improved cookstoves (ICS)	4
Micro‐hydro	3
Engine	1
General solar products	1
Solar market gardens (SMG)	1
Solar oven	1

*Note:* The sum of the studies in the above table is greater than 47 as multiple studies contained treatments with different technologies. In these cases, the study was counted towards each technology.

Solar mini‐grids were the third most common technology and consisted of small‐scale electricity networks that distributed power generated by solar panels to multiple users, such as the PVPA programme in China (Xiao et al. 2023). Solar improved cookstoves were also evaluated in four studies and included cookstoves, such as those used in the CAPS trial in Malawi, which provided a stove with a battery‐powered fan charged via a solar panel (Kelly 2018). Micro‐hydro was the technology of focus in three studies, all of which evaluated the impact of household electricity provision from micro‐hydro plants.

Four technologies were evaluated in just a single study. One programme provided an engine to farmers which could run off renewable oils (Araujo Bonjean et al. 2015), while another provided information and support to entrepreneurs selling solar products, though the exact nature of these products was never disclosed, and so they were coded as ‘general solar products’ (Bensch et al. 2021). Other studies evaluated programmes that provide solar ovens and solar market gardens (SMG), which integrate solar‐powered irrigation systems to enhance agricultural productivity (Burney et al. 2017).

##### 5.1.2.5 Intervention Scale

We classified the programmes evaluated within the included studies into three levels: local, regional and national, to indicate the scale at which the programme operated. Both national and regional level programmes were the most common, appearing in 18 and 17 studies respectively, while 11 studies evaluated programmes operating at the local level. Local‐level programmes were typically targeted towards specific communities, such as the CAPS trial conducted in Karonga District, located within the Northern Region of Malawi (Kelly 2018). Regional‐level programmes included broader geographical areas aiming to address common regional challenges. For instance, ACCIONA Microenergía Peru (AMP) targeted the region of Cajamarca to increase access to electricity in rural communities far from the national grid (Arraiz and Calero 2015). National‐level programmes covered entire countries and were typically led by the national government. For instance, the PVPA programme implemented by the Chinese Government (Xiao et al. 2023).

##### 5.1.2.6 Supply/Demand

The majority of programmes were implemented with a focus on the demand side of off‐grid technologies. Forty‐two programmes aimed to either increase demand for off‐grid technologies through providing information on their benefits or through overcoming the financial barrier to off‐grid technology ownership through the direct provision or financial mechanism. On the other hand, two programmes focused on supply‐side interventions, supporting those who supply off‐grid systems/products. For instance, the EnDev‐K programme in Kenya provided various business development services to entrepreneurs interested in selling solar products, including a mobilisation campaign, technical and business training, assistance to business set‐up, ongoing support and education of consumers (Bensch et al. 2016).

In addition, three studies in our sample evaluated programmes integrating both supply and demand‐side interventions. For example, the Yeelen Ba programme in Burkina Faso set up a company aiming to provide solar energy to households and small businesses while it also implemented information dissemination campaigns through village meetings and systems/product demonstrations (Bensch et al. 2015).

##### 5.1.2.7 Intervention Target

We captured details on both the target actor of a study, as well as whether they targeted an equity population. In terms of actors, 32 studies provided interventions towards households while 9 targeted communities. Fewer, just seven, targeted individuals or individual traders while two targeted schools.

Very few studies were targeted towards an equity population. Just 4 targeted either primary or secondary school students (e.g., Stojanovski et al. 2021), while three targeted women (e.g., Burney et al. 2017). The remaining 40 studies did not target any particular equity population.

##### 5.1.2.8 Implementation Agency

The agency that implemented programmes within our included studies was also captured when reported (Table 7). Government agencies were the most common implementers, appearing in 15 programmes. This includes both programmes implemented in a government's own country, such as the PVPA programme implemented by the Government of China, as well as programmes that relied on foreign government agencies, such as the EnDev‐K programme, which was implemented in Kenya by Deutsche Gesellschaft für Internationale Zusammenarbeit (GIZ) and the non‐profit SNV Netherlands Development Organisation (Bensch et al. 2021). For‐profit firms implemented 13 programmes. For instance, the D20g Go‐to‐Market pilot project was implemented by the social enterprise d.light to understand the social effects of small SHS in Uganda (IDInsight 2018).

**Table 7:** Number of studies by implementation agency.

Implementation agency	No. of studies
Government agencies	15
For‐profit firms	13
Non‐profit organisations	7
Charitable foundations	2
International aid agency	1

Non‐profit organisations implemented seven programmes. An example is the RCT in Kenya implemented by GiveWatts, which assessed the effects of solar products on education, time use and income outcomes (Lucchino 2017). Two programmes were implemented by charitable foundations and one by an international aid agency.

#### 5.1.3 Excluded Studies

Given that our EGM report provides a breakdown of excluded studies based on full text screening (including examples of specific cases), we instead focus on studies that were ‘near misses’ for our review. These studies have been screened out from our review based on the additional criteria we have added since our EGM (further examples are available in Appendix G).

Based on their outcomes, 16 papers were excluded from our review. These studies evaluated outcomes that were relevant to the EGM but excluded from our review's energy access, climate and socio‐economic groups. For instance, only two papers evaluated an *energy innovations* outcome (a category from our EGM), which was not included in the review's scope. These papers focused on the evaluation of government subsidies in China, specifically provided to businesses to increase research and development output (Wu et al. 2023; Jiang et al. 2021). Other papers were ultimately not included due to the outcome prioritisation process outlined in Appendix B. For instance, studies that measured health outcomes but through indicators that were not common among other papers reporting health outcomes, such as nutrition outcomes (e.g., Alaofè et al. 2019) and low birth weight (e.g., Best et al. 2022).

Aside from the outcomes measured, other papers were excluded from our review due to the off‐grid technology that they evaluated. While many technologies were common across papers, six studies evaluated niche technologies. Studies on solar dryers (Matavel et al. 2022; Nagwekar et al. 2020), solar suitcases (Rokicki et al. 2019), solar cold storage systems (Takeshima et al. 2021) and solar mosquito systems (Briët et al. 2017; Homan et al. 2016) were excluded.

### 5.2 Risk of Bias in Included Studies

For each estimate in our review, we conducted a risk of bias assessment, the results of which are outlined below. Appendix H provides the full breakdown of the assessment for each study.

**Figure 4:**
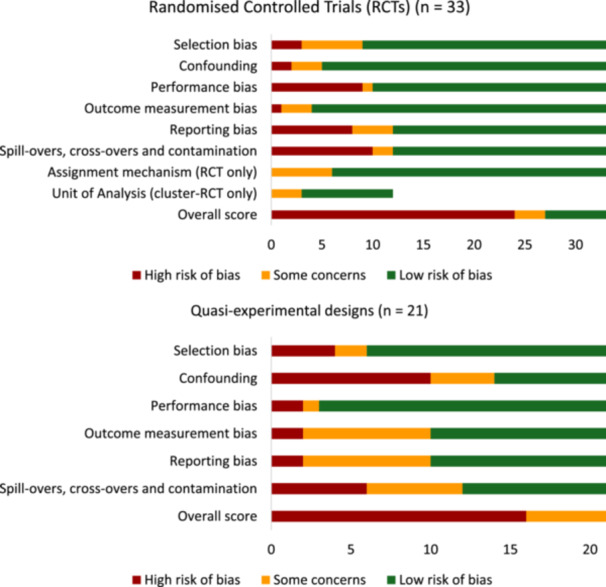
Summary of risk of bias assessment for RCTs and QEDs. A study was coded as ‘Low risk of bias’ when it provided clear, direct and robust evidence of being free from bias or when it likely avoided the bias, but minor concerns or slight limitations were still present. Conversely, ‘High risk of bias’ indicates that the study either did not provide clear evidence or was likely affected by bias. ‘Some concerns’ was used if a study lacked details to decide on a specific dimension. Two dimensions, assignment mechanism and unit of analysis, apply only to RCTs, with the latter dimension applicable only to cluster allocations.

The risk of bias assessment examined whether the design, implementation, analysis or reporting of the studies might introduce bias into their estimated effects. We conducted 54 risk of bias assessments, comprising 33 from RCTs and 21 from QEDs.^4^ Two papers were assessed twice. Figure 4 illustrates the risk of bias assessment dimensions for all included studies.

In our overall rating, we found that both RCTs (69%) and QEDs (76%) had similar proportions of studies assessed as high risk of bias. However, there was a notable difference when it came to low risk of bias assessments: while 19% of RCTs were assessed as low risk, only one QED study received a low risk of bias assessment. Similarly, 13% of RCTs and 19% of QEDs were assessed as having some bias concerns.

Spill‐overs, cross‐overs and contamination bias was the dimension most frequently assessed as high risk within RCTs (*n* = 10); in turn, confounding bias was most common in QEDs (*n* = 10). While many of the included studies were assessed as having a high risk of bias, in many cases this was due to studies failing to meet only one of the dimensions (in 14 of the 24 RCTs and in 7 of the 16 QEDs assessed as having a high risk of bias). Given the large number of studies with a high risk of bias, we did not exclude these from our analyses. We also conducted moderator analysis based on a study's overall risk of bias, where possible.

### 5.3 Synthesis of Results

#### 5.3.1 Quantitative Synthesis

We present the results of the quantitative synthesis organised according to the three main outcome groups of the review: energy access, climate and socio‐economic development outcomes. If meta‐analysis was not possible due to insufficient data, we present a narrative description of the outcome. Where meta‐analysis was feasible, the results for each outcome include a discussion of moderator, sensitivity and publication bias analyses, if applicable.

We assessed 13 moderators to help explain differences in results among studies, including contextual factors (such as the region and country of intervention) and key features of the intervention (such as technology type, level of implementation, intervention mechanism and scale, target actor and population, supply/demand focus and implementing agency), as well as study characteristics (overall risk of bias, study design and year of publication). Complementary information, including funnel plots and tables of these analyses, is provided in Appendix I.

As a summary of all analyses conducted, Table 8 displays the meta‐analysis and publication bias results across outcome categories covered in subsequent sections. Due to a limited number of studies, we were only able to assess publication bias for nine of the outcomes included in this review. Among these, evidence of publication bias was found in just one outcome—time spent studying, applicable to both children and adults—which reduces the confidence in the reliability and generalisability of the pooled effect for this outcome.

**Table 8:** Summary of the meta‐analyses results of off‐grid electrification technology interventions.

Group	Category	Outcome	*N*	SMD	95% CI	*p*‐value	Publ. bias
Energy access	Energy consumption	Hours of lighting use	12	0.16	−0.08, 0.40	0.187	No
		**Kerosene consumption**	**6**	**−0.57**	**−0.92, −0.23**	**0.001**	**N/A**
	Energy expenditure	Energy expenditure	18	0.15	−0.03, 0.32	0.104	No
	Technology uptake or upgrade	**Uptake compared to no intervention**	**2**	**0.18**	**0.11, 0.24**	**< 0.001**	**N/A**
		**Uptake compared to lower price paid/higher subsidy**	**3**	**−0.52**	**−0.76, −0.29**	**< 0.001**	**N/A**
	Technology usage	**Technology use**	**5**	**0.61**	**0.13, 1.09**	**0.012**	**N/A**
		Technology use (light only)	3	0.24	−0.13, 0.61	0.199	N/A
	Energy security	**Access**	**3**	**0.20**	**0.01, 0.34**	**0.036**	**N/A**
		Reliability	2	0.29	−0.16, 0.73	0.206	N/A
		Affordability	2	−0.01	−0.10, 0.08	0.830	N/A
Climate	Air quality	PM 2.5	5	0.16	−0.01, 0.32	0.070	N/A
	GHG emissions	CO_2_ emissions	1	N/A			N/A
Socio‐economic	Income	**Household (or personal) income**	**13**	**0.06**	**0.02, 0.10**	**0.007**	**No**
	Allocation of time	Time spent working (remunerated)	14	0.01	−0.03, 0.06	0.582	No
		Time spent on domestic work	14	0.02	−0.03, 0.06	0.461	No
		Domestic work: cooking	4	N/A			N/A
		Domestic work: caretaking	2	N/A			N/A
		Domestic work: collecting fuel	3	N/A			N/A
		Time spent on leisure	13	0.03	−0.03, 0.09	0.363	N/A
		Leisure: watching TV	2	N/A			N/A
		Leisure: radio listening	3	N/A			N/A
		Leisure: reading	2	N/A			N/A
		Leisure: playing	2	N/A			N/A
		Time spent resting/sleeping	6	−0.03	−0.13, 0.06	0.470	N/A
		**Time spent studying**	**13**	**0.11**	**0.03, 0.19**	**0.005**	**Yes**
		**Time spent studying (children only)**	**12**	**0.14**	**0.05, 0.23**	**0.003**	**No**
	Education	School attendance	6	0.03	−0.02, 0.09	0.246	N/A
		Test scores	5	0.00	−0.02,0.03	0.860	N/A
	Health	Respiratory illness	10	0.04	−0.02, 0.10	0.163	No
	Women's empowerment	**Women's empowerment**	**3**	**0.11**	**0.03, 0.18**	**0.008**	**N/A**

*Note:* Outcome subcategories in bold have a statistically significant SMD at a 5% significance level. N/A indicates that we did not conduct a meta‐analysis or publication bias analysis for these outcome categories. Publication bias tests were only conducted when there are at least 10 or more studies (or effects).

Abbreviations: 95% CI, confidence interval; *n*, number of estimates included in the analysis; SMD, standardised mean difference.

##### 5.3.1.1 Energy Access Group

The energy access outcome group covers measures of a household's access and utilisation of energy. This includes measures of use and uptake of off‐grid technologies and energy in general, energy security and expenditure on different energy sources.

###### 5.3.1.1.1 Energy Consumption

To assess the effects of off‐grid energy interventions on energy consumption, we considered the estimates reported for lighting hours and kerosene consumption. We found a significant reduction in kerosene consumption but no effect on lighting hours, as detailed below.

####### 5.3.1.1.1.1 Hours of Lighting Use

**Figure 5:**
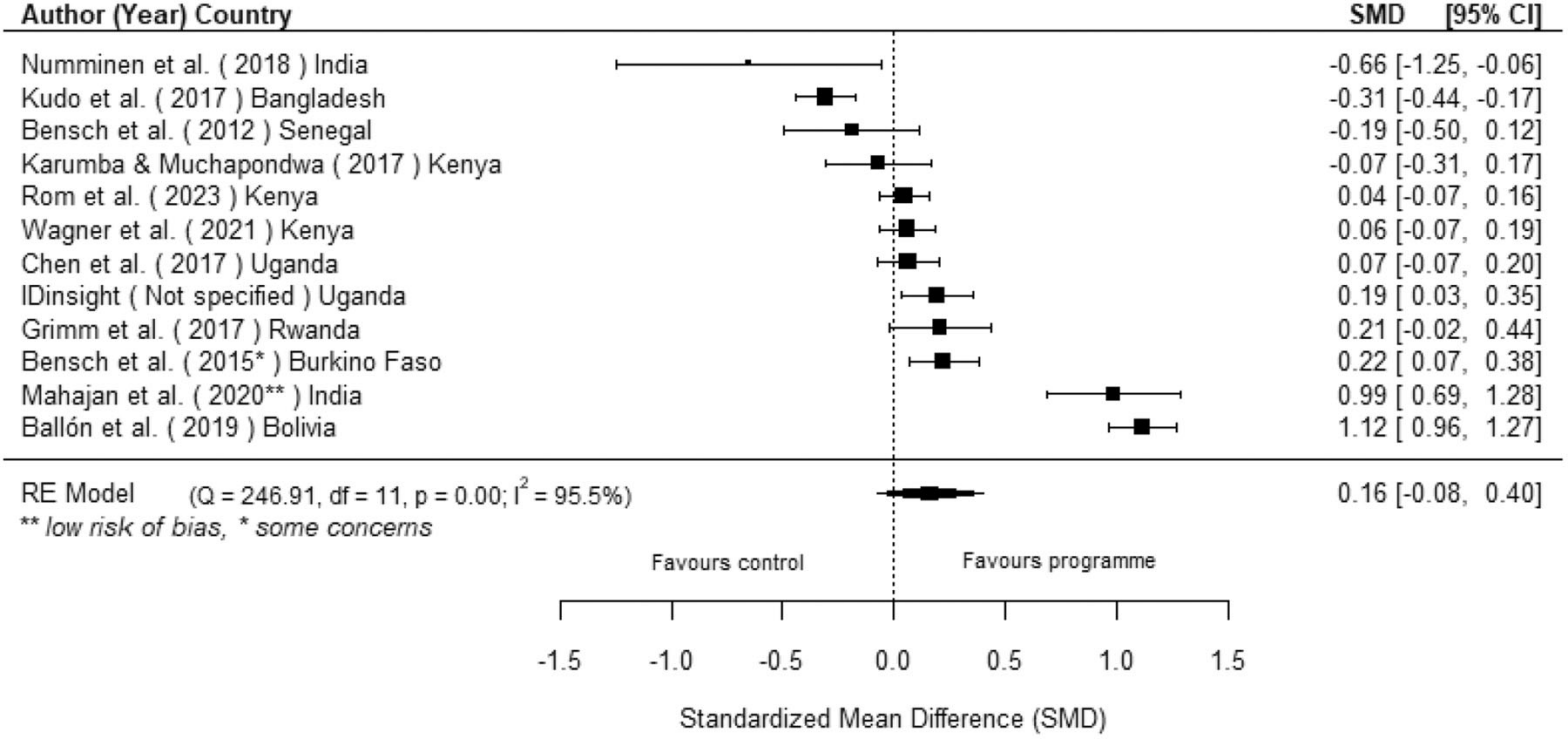
Forest plot showing estimates of the random‐effects model for the effect of off‐grid interventions on hours of lighting use.

We found 12 studies evaluating the effects of off‐grid interventions on the number of hours per day a household or individual uses lighting. Off‐grid electricity technologies did not change the hours of lighting use. The overall average weighted effect was small and positive but not statistically significant ($\hat{\mathrm{SMD}}$ = 0.16; 95% CI: −0.08 to 0.40; *p* = 0.187). The heterogeneity among the studies was substantial (*I* ^2^ = 95.54%). We rated all but two of the included effect sizes as having a high risk of bias (Figure 5).

While one study reported a higher estimate than that of the other studies (Ballón et al. 2019), removing this study would reduce the overall average effect but remain within the confidence interval of the main effect, suggesting that this study is not overly influential ($\hat{\mathrm{SMD}}=0.08,\;p=0.34$).

None of the moderator variables significantly contributed to the variation in estimates. The results from all but two of the included studies for this analysis were rated as having a high risk of bias. The average effect between the two studies with some concerns or low risk of bias was much larger, but, because of the substantial level of heterogeneity between both studies, the effect on the use of hours of light was still not statistically different from zero (${\hat{\mathrm{SMD}}}_{\mathrm{Low}\;\mathrm{or}\;\mathrm{med}\;\mathrm{ROB}}=0.60;\;95\%\;\mathrm{CI:}-0.15\;\mathrm{to}\;1.35;\;p=0.12$).

####### 5.3.1.1.1.2 Kerosene Consumption

**Figure 6:**
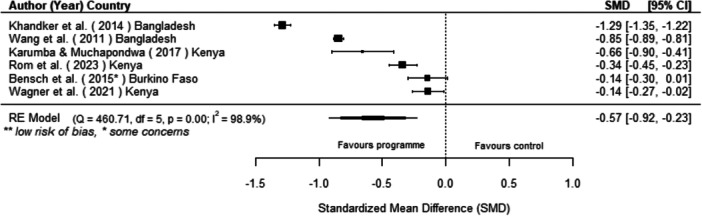
Forest plot showing estimates of the random‐effects model for the effect of off‐grid interventions on kerosene consumption.

We found six studies measuring the effects of off‐grid interventions on litres of kerosene consumed or purchased. Removing barriers to off‐grid electricity technologies through direct provision, market expansion, financing opportunities or information campaigns reduces kerosene consumption. The overall weighted effect was large, negative and statistically significant ($\hat{\mathrm{SMD}}=-0.57;\;95\%\mathrm{CI:}\;-0.92\;\mathrm{to}\;-0.23;\;p=0.001$). All the effect sizes in this analysis were negative and varied between −1.29 and −0.14 standard deviations. However, the heterogeneity among the studies was substantial ($I^{2}=98.91$%). Further, the effects from all included studies but one were rated as having a high risk of bias, and the effect size from the only study rated as having some concerns was not statistically different from zero (Figure 6).

None of the studies were overly influential or identified as an outlier, so results did not change when removing one study at a time. There was no difference in the effect sizes based on the type of technology, the scale of the intervention or its supply/demand focus. However, moderator analysis suggested that the effect was on average smaller in SSA interventions compared to South Asia (p<0.01), but greater for interventions implemented by a government agency with or without for‐profit firms compared to other implementation agencies (p<0.01). Also, adding an informational component to financial opportunities for off‐grid technologies further reduced kerosene consumption when compared to interventions providing only financial support (p<0.01). Other factors could not be tested as there was not enough variation.

###### 5.3.1.1.2 Energy Expenditure

**Figure 7:**
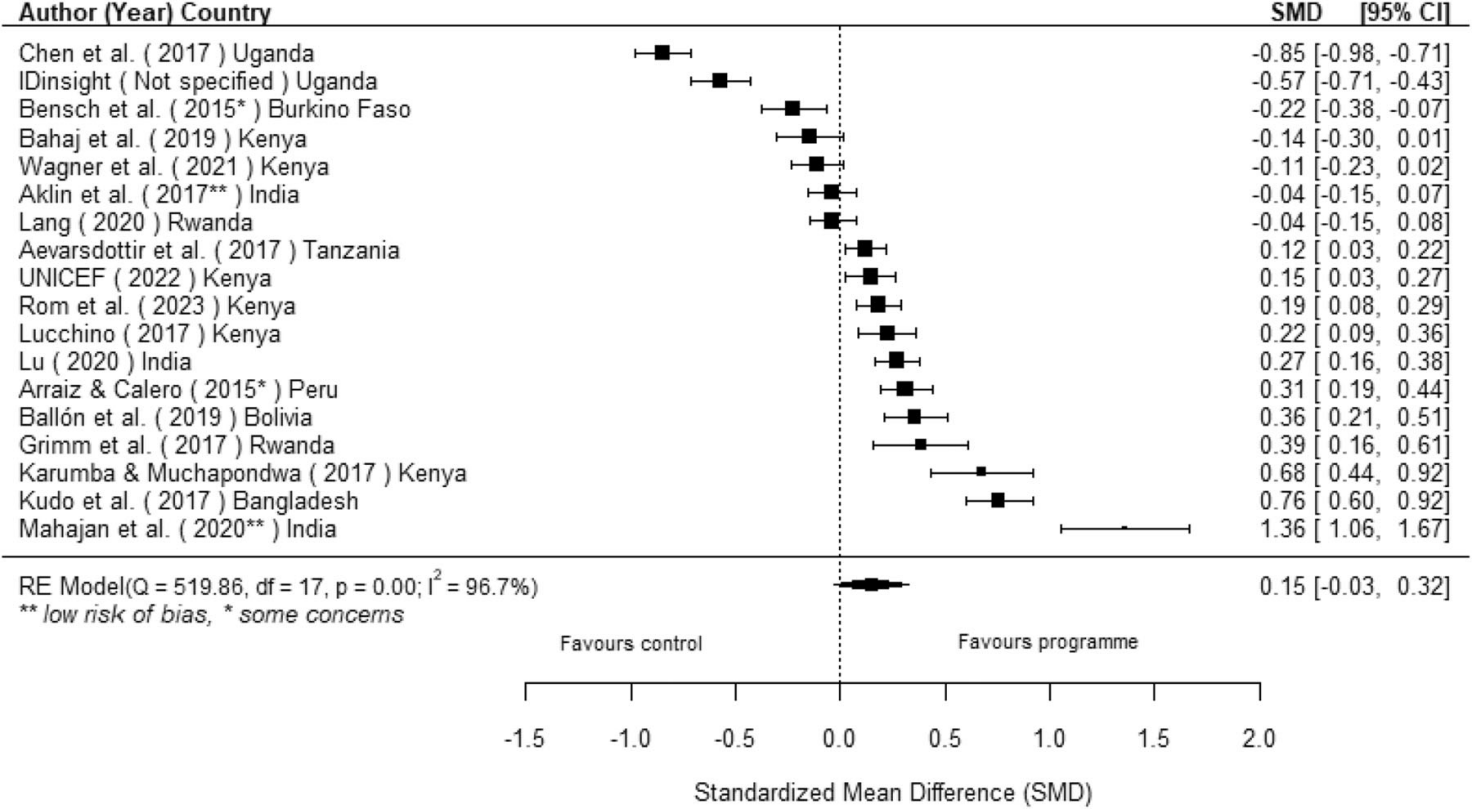
Forest plot showing estimates of the random‐effects model for the effect of off‐grid interventions on energy expenditure. We reversed the signs of effects on energy expenditure as we consider a reduction in this outcome to be positive.

A total of 18 studies reported 36 estimates of the effect on aggregated energy expenditure. For six studies reporting multiple estimates for different populations, treatment arms, follow‐up surveys or energy sources, we computed a composite measure to be used as an independent effect in the analysis. The random effects model suggested a reduction in energy expenditure. The overall average effect was small but not statistically significant ($\hat{\mathrm{SMD}}=0.15;\;95\%\;\mathrm{CI:}\;-0.03\;\mathrm{to}\;0.32;\;p=0.10$) and there was a large and significant level of heterogeneity among studies ($I^{2}=96.73$%). We rated all but four of the included effect sizes as having a high risk of bias (Figure 7).

We identified two potential outliers in this analysis (Chen et al. 2017; Mahajan et al. 2020). Removing the study by Chen et al. (2017) would increase the overall average effect and make it statistically significant ($\hat{\mathrm{SMD}}=0.20;\;p=0.01$). On the other hand, removing Mahajan et al. (2020) would reduce the overall average effect, but it would still not be significant ($\hat{\mathrm{SMD}}=0.08;\;p=0.04$). However, no study could be considered overly influential, as the average effect from leaving each of these out one at a time still falls under the confidence interval of the main effect. Therefore, for moderator analysis, we included all studies.

Most of the studies in this analysis came from SSA (k=12). Compared to these, the effects from LAC (k=2) were not statistically different, but the studies from South Asia (k=4) reported larger reductions in expenditure on average (*p* = 0.02). By country, the studies from Uganda reported a smaller reduction in expenditure on average (*p* < 0.01). Studies using a QED report larger increases in energy expenditure compared to experimental studies (*p* = 0.03). Other significant moderators were the type of technology and the intervention mechanism. The effects from studies evaluating SHS were significantly different compared to the effects for all other technologies (*p* < 0.01), but the average effect was not statistically significant (${\hat{\mathrm{SMD}}}_{\mathrm{SHS}}=-0.24;\;95\%\;\mathrm{CI:}-0.57\;\mathrm{to}\;0.09;\;p=0.15;\;k=6$). On the other hand, solar lamp/lanterns with mobile phone chargers had a significantly larger effect compared to other technologies (*p* = 0.02), and the average effect was large and statistically significant (${\hat{\mathrm{SMD}}}_{\mathrm{lamp}+\mathrm{charger}}=0.54;\;95\%\;\mathrm{CI:}\;0.21\;\mathrm{to}\;0.87;\;p<0.01;\;k=5$). The studies based on financial mechanisms showed a significantly different effect on energy expenditure compared to interventions that directly provided an energy technology (*p* = 0.04), but again, the average effect of providing credit or subsidies was not statistically significant (${\hat{\mathrm{SMD}}}_{\mathrm{financial}}=-0.09;\;95\%\;\mathrm{CI:}-0.35\;\mathrm{to}\;0.17;\;p=0.51;\;k=7$). Further, the effects of interventions implemented by for‐profit firms were significantly different than those of all other types of implementation agencies (*p* = 0.02), but the average effect was also not statistically significant (${\hat{\mathrm{SMD}}}_{\mathrm{For}-\mathrm{profit}}=-0.14;\;95\%\mathrm{CI:}-0.44\;\mathrm{to}\;0.16;\;p=0.37;\;k=7$). The technology level, scale, target actor, target population, year of publications and risk of bias rating were not significant moderators of the effect on energy expenditure.

Only four studies were not rated as having a high risk of bias, pooling the effect sizes of these effects resulted in a larger but much less precise and, therefore, still not statistically significant average effect on energy expenditure (${\hat{\mathrm{SMD}}}_{\mathrm{Low}\;\mathrm{or}\;\mathrm{med}\;\mathrm{ROB}}=0\text{.}33;\;95\%\;\mathrm{CI:}\;-0\text{.}11\;\mathrm{to}\;0\text{.}77;\;p=0\text{.}10$).

###### 5.3.1.1.3 Technology Uptake or Upgrade

We synthesise the effects of off‐grid interventions, including measures of the uptake of a new technology or the replacement/upgrade of an old technology, using two different comparison groups. We explored measures related to technology use in the next section.

####### 5.3.1.1.3.1 Technology Uptake or Upgrade Compared to No Intervention

**Figure 8:**
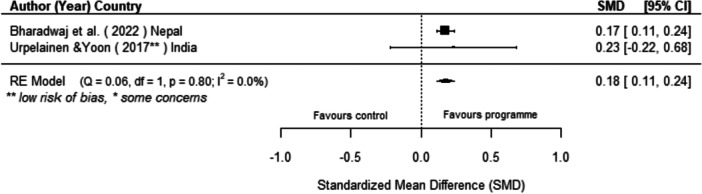
Forest plot showing estimates of the random‐effects model for the effect of off‐grid interventions on technology uptake or upgrade, compared to no intervention.

We included two studies evaluating the effects on measures of purchase or adoption of an off‐grid technology compared to no intervention (i.e., business as usual). The overall average effect from these two studies was small, positive and statistically significant, suggesting that interventions increased the technology uptake or upgrade ($\hat{\mathrm{SMD}}=0.18;\;95\%\;\mathrm{CI:}\;0.11\;\mathrm{to}\;0.24;\;p<0.01$). There was no level of heterogeneity in this analysis ($I^{2}=0.0$%). The effect size from one study was rated as having a high risk of bias, while the other effect size was rated as having a low risk of bias (Figure 8).

Both interventions included in this analysis were implemented in South Asia, looked at the effects of SHS from the demand‐side, targeting households. Other intervention characteristics varied between both studies but the small sample did not allow for sensitivity or moderator analysis.

####### 5.3.1.1.3.2 Technology Uptake or Upgrade Compared to Lower Price Paid/Higher Subsidy

**Figure 9:**
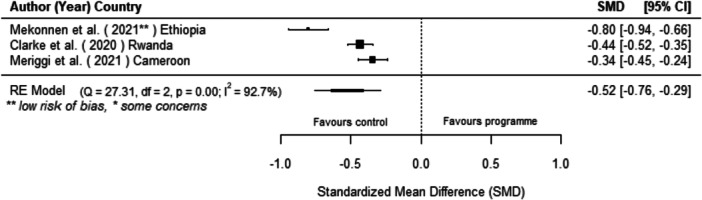
Forest plot showing estimates of the random‐effects model for the effect of off‐grid interventions on technology uptake or upgrade, compared to lower price paid/higher subsidy.

We identified three studies evaluating the effects on purchase or adoption of an off‐grid technology comparing different prices paid or subsidy levels. The overall average effect was large, negative and statistically significant ($\hat{\mathrm{SMD}}=-0\text{.}52;\;95\%\;\mathrm{CI:}\;-0\text{.}76\;\mathrm{to}\;-0\text{.}29;p<0\text{.}01$) and there was substantial heterogeneity in the effects used for this analysis ($I^{2}=92.70\%$). We found a significantly lower adoption of technologies when compared to technologies offered with higher subsidies or lower prices paid (Figure 9). These results should be taken with caution given the small sample size used in the analysis and the high risk of bias identified in two of the three included effect sizes.

All the studies included in this analysis were implemented in SSA, looked at the effects of solar lamps/lanterns from the demand‐side, households or individuals. Other intervention characteristics varied between studies but the small sample did not allow for moderator analysis. One study (Mekonnen et al. 2021) may be a potential outlier in the context of this model. However, none of the studies could be considered overly influential and removing Mekonnen et al. did not change the overall results ($\hat{\mathrm{SMD}}=-0.40,\;p<0.01$).

###### 5.3.1.1.4 Technology Usage

We explored the effects of off‐grid interventions on the usage of a technology after it has been received or bought. This includes usage measures of alternative technologies, for example, those that the intervention aimed to replace. We found a small but positive effect for usage of any technology, but a non‐statistically significant result for usage of lights.

####### 5.3.1.1.4.1 Technology Use

**Figure 10:**
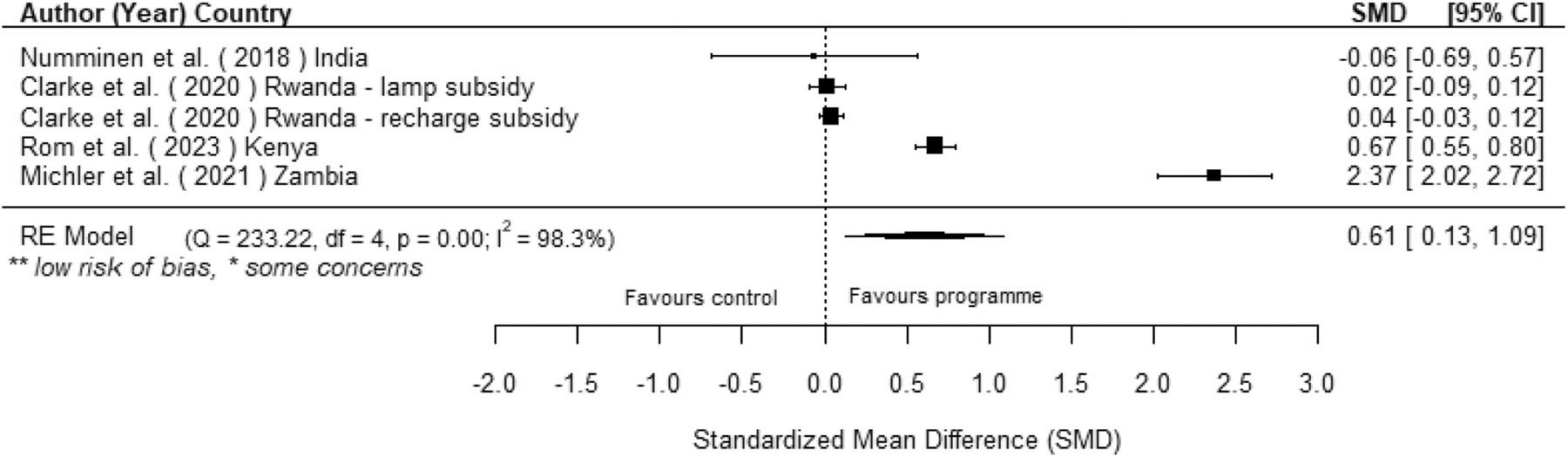
Forest plot showing estimates of the random‐effects model for the effect of off‐grid interventions on the usage of technologies. Two independent effect sizes from the same study were included in the analysis (Clarke et al. 2020). These came from different field experiments: one randomly varied the price of a solar light through subsidies and the other one provided the light for free and randomly assigned recharge coupons with reduced fees.

We included five independent effect sizes from four studies in the analysis of the effects on technology usage, including electricity, LED light, solar light and solar stove. Off‐grid interventions increased the household use of these technologies. The overall average effect based on the random‐effects model was large, positive and statistically significant ($\hat{\mathrm{SMD}}=0.61;\;95\%\;\mathrm{CI:}0.13\;\mathrm{to}\;1.09;\;p=0.01$). There was substantial heterogeneity in the effects used for this analysis ($I^{2}=98.28\%$) and all were rated as having a high risk of bias (Figure 10).

Our main result was driven by one potential outlier in the context of this model (Michler et al. 2021), which measured the effect on the use of solar stoves. According to the Cook's distances, this study could be considered overly influential; removing it would reduce the magnitude of the average effect and it would no longer be statistically significant ($\hat{\mathrm{SMD}}=0.20,\;p=0.25$). Further, all the effects from studies in this analysis were considered of high risk of bias.

There was insufficient variation in these studies to test most of the factors identified as potential moderators to explain differences in results. All interventions had a demand focus targeting households, and most were implemented in SSA at a regional level. The implementation agency was not a significant predictor of the effect, nor was the study's year of publication. When looking at the intervention mechanisms, however, direct provision was associated with a larger effect compared to other mechanisms (p=0.02).

####### 5.3.1.1.4.2 Technology Use (Light Only)

**Figure 11:**
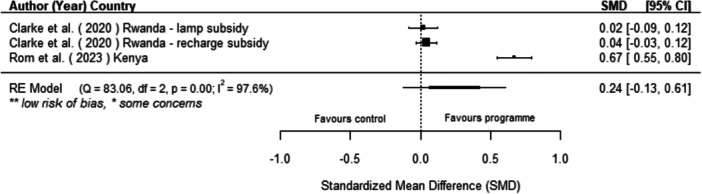
Forest plot showing estimates of the random‐effects model for the effect of off‐grid interventions on the usage of lighting technologies. Two independent effect sizes from the same study were included in the analysis (Clarke et al. 2020). These came from different field experiments: one randomly varied the price of a solar light through subsidies and the other one provided the light for free and randomly assigned recharge coupons with reduced fees.

We excluded the study looking at electricity usage to focus on the effects of the use of light, LED or solar alone. The three effects for this analysis were all positive and ranged from 0.02 to 0.67 standard deviations. However, since two of the effects were not statistically significant, nor was the average effect size from the random‐effects model. The pooled effect on light usage was moderate and positive but not statistically different from zero ($\hat{\mathrm{SMD}}=0.24;\;95\%\;\mathrm{CI:}\;-0.13\;\mathrm{to}\;0.61;\;p=0.20$). Heterogeneity for this analysis was still substantial ($I^{2}=97.59\%$) and we rated all the included effect sizes as having a high risk of bias (Figure 11).

When we focused on the use of light only, the study by Rom et al. (2023) may be considered a potential outlier. Removing this study reduced the magnitude of the overall average effect ($\hat{\mathrm{SMD}}=0.03,\;p=0.26$), which was still statistically insignificant and came from a single high risk of bias study.

###### 5.3.1.1.5 Energy Security

We defined energy security as the uninterrupted availability of energy sources at an affordable price. Includable measures of energy security were individual indicators of energy access, quality and reliability. We found mixed results, with a small but positive and significant effect for energy access.

####### 5.3.1.1.5.1 Access

**Figure 12:**
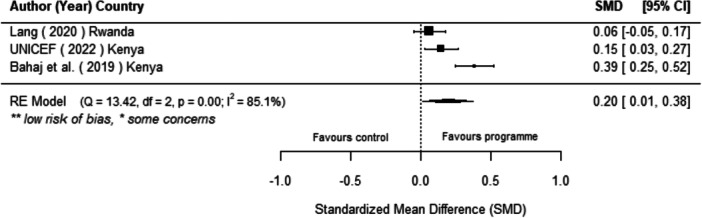
Forest plot showing estimates of the random‐effects model for the effect of off‐grid interventions on energy access.

Measures of energy access reported by the three studies in this analysis include household access to electricity, household access to energy for lighting and household connection to the grid. The observed effect sizes were positive and ranged from 0.063 to 0.387. The estimated average effect based on the random‐effects model was small, positive and statistically significant ($\hat{\mathrm{SMD}}=0\text{.}20;\;95\%\;\mathrm{CI:}\;0\text{.}01\;\mathrm{to}0\text{.}38;\;p=0\text{.}04$). The true effect appears to be heterogeneous ($I^{2}=85.06\%$). Hence, although the average effect is estimated to be positive, in some studies the true effect may be negative (Figure 12). Further, we rated all the effect sizes in this analysis as having a high risk of bias.

The positive result from this analysis should also be taken with caution due to the small sample size used in the model, and all effects being assessed as having a high risk of bias. There was not enough variation in these studies to allow for moderator analyses. However, removing the study by Bahaj et al. (2019), which may be a potential outlier, would reduce the overall average effect, but the effect would still be positive and statistically significant ($\hat{\mathrm{SMD}}=0.10,\;p=0.01$).

####### 5.3.1.1.5.2 Reliability

**Figure 13:**
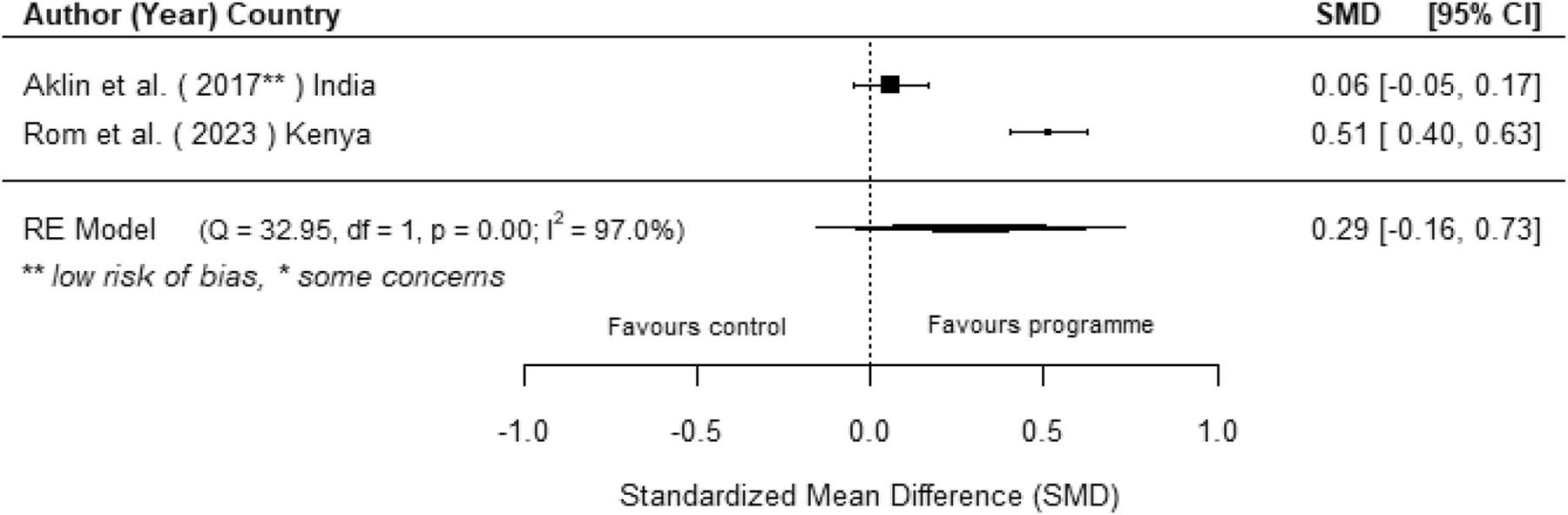
Forest plot showing estimates of the random‐effects model for the effect of off‐grid interventions on energy reliability.

Two studies reported outcomes on energy reliability using as measures the number of hours a day that electricity is usually available for the household and the probability of lighting interruption. The pooled effect was moderate and positive (improved reliability) but not statistically significant ($\hat{\mathrm{SMD}}=0.29;\;95\%\;\mathrm{CI:}\;-0.16\;\mathrm{to}\;0.73;\;p=0.21$). Even with only two studies, the level of heterogeneity in this analysis was substantial ($I^{2}=96.97\%$). One of the estimates in this analysis was rated as having a low risk of bias and the other as having a high risk of bias (Figure 13). The small sample size used in the model did not allow for sensitivity or moderator analysis.

####### 5.3.1.1.5.3 Affordability

**Figure 14:**
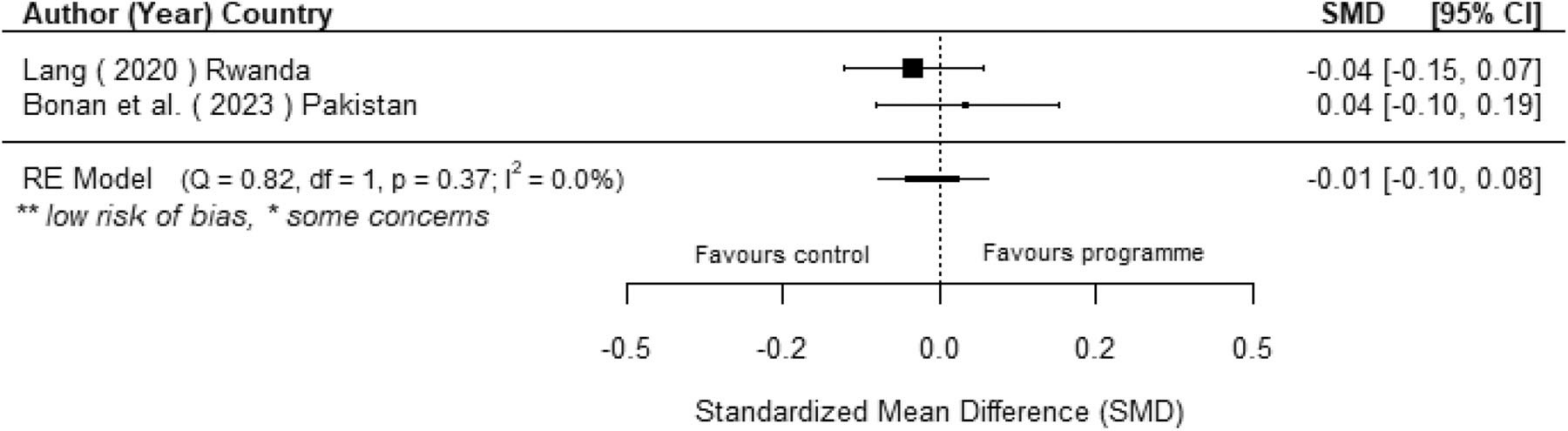
Forest plot showing estimates of the random‐effects model for the effect of off‐grid interventions on energy affordability.

Two studies reported insignificant effects on energy affordability using measures of the average successive days with zero balance before top‐up and an indicator for borrowing for solar energy. The pooled effect was very small and not statistically significant ($\hat{\mathrm{SMD}}=-0\text{.}01;\;95\%\;\mathrm{CI:}-0\text{.}10\;\mathrm{to}\;0\text{.}08;\;p=0\text{.}83$). There was no heterogeneity in this analysis ($I^{2}=0.00\%$). The small sample size used in the model did not allow for sensitivity or moderator analysis, and we rated the effect sizes from both studies as having a high risk of bias (Figure 14).

##### 5.3.1.2 Climate Group

Among climate outcomes relevant to this review, we included measures of air quality and GHG emissions. Few studies evaluated a climate outcome, and we did not find statistically significant results.

###### 5.3.1.2.1 Air Quality

**Figure 15:**
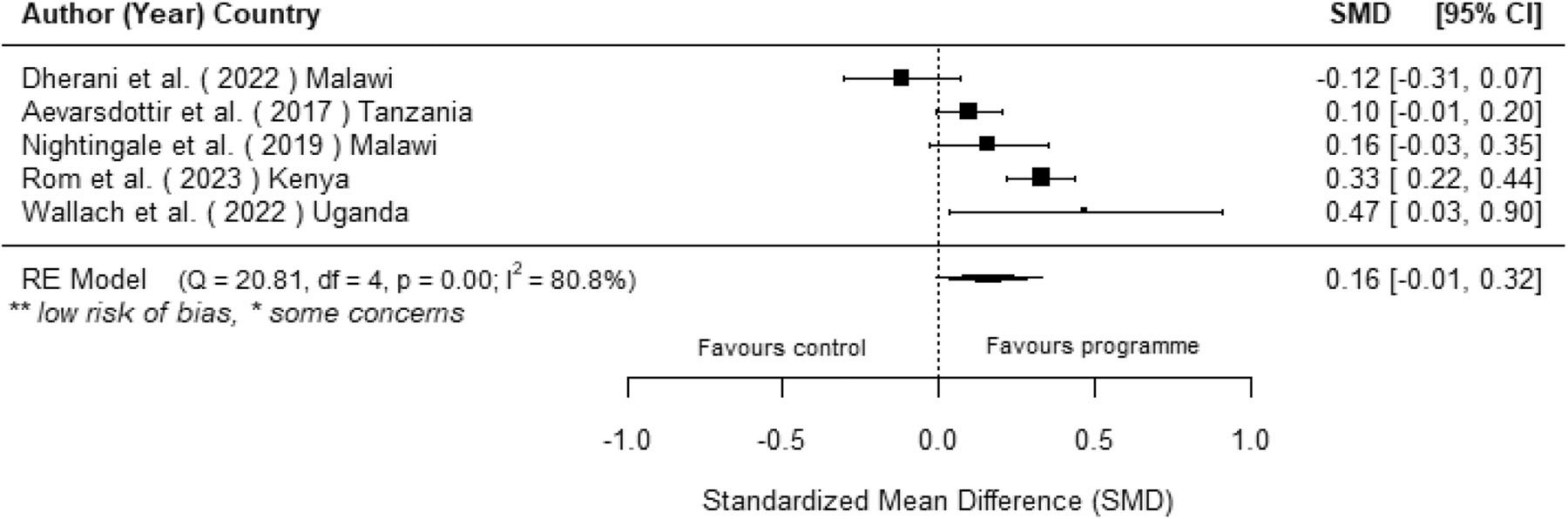
Forest plot showing estimates of the random‐effects model for the effect of off‐grid interventions on air quality. We reversed the signs of effects on air pollution measures as we consider a reduction in this outcome to be positive and a reflection of improved air quality.

We found five studies exploring the effects of off‐grid technologies on indoor air pollution using particulate matter of size 2.5 micrometres in diameter or smaller in the air (PM2.5). There is no consistent evidence of an increase or reduction in air quality associated with off‐grid technologies. The average effect size was not statistically different from zero ($\hat{\mathrm{SMD}}=0\text{.}16;\;95\%\;\mathrm{CI:}\;-0\text{.}01\;\mathrm{to}\;0\text{.}32;\;p=0\text{.}07$) and the heterogeneity level was large and significant ($I^{2}=80.78\%$). This result should be taken with caution as all effects used in the model were rated as having a high risk of bias (Figure 15).

The effects from the two studies evaluating solar improved cookstoves were not statistically different from the effects found for solar lamps/lanterns with or without phone chargers in three studies (p=0.16). The intervention mechanism did not help explain differences in the results either, as effects from direct provision in three studies did not differ from other mechanisms in the remaining studies (p=0.66). There was not enough variation in other factors to allow for moderator analysis. All studies came from SSA, were implemented at the household level with a demand focus and were rated as having a high risk of bias. Most studies did not target a specific population and did not specify the implementation agency. Finally, none of the studies were considered potential outliers, and leaving out one study at a time did not change the results.

###### 5.3.1.2.2 GHG Emissions

We identified one study in the outcome category GHG emissions through measures of carbon related emissions (CO_2_) and non‐carbon related emissions, for example, methane (CH_4_), nitrous‐oxide (N_2_O) and fluorinated gases (Rom et al. 2023). The study was implemented in SSA and evaluated the effect of owning a solar lamp on CO_2_ emissions. We rated this study as having a high risk of bias.

##### 5.3.1.3 Socio‐Economic Development Group

The group of socio‐economic development outcomes includes measures of income, time allocation (for remunerated and domestic work, leisure, resting or sleeping, and studying), education (school attendance and test scores), health (respiratory illness) and women's empowerment.

###### 5.3.1.3.1 Income

**Figure 16:**
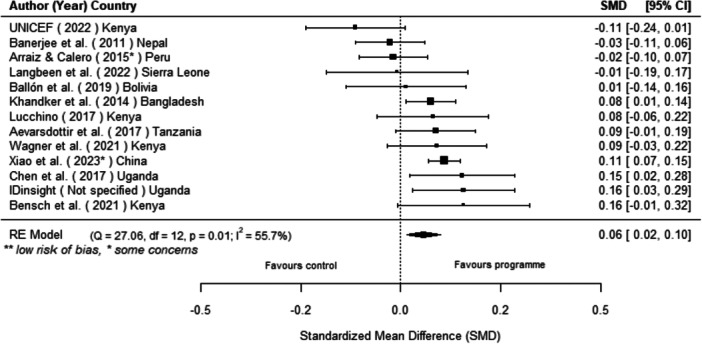
Forest plot showing estimates of the random‐effects model for the effect of off‐grid interventions on income.

We found 13 studies reporting estimates of the effect of off‐grid technologies on measures of a household or individual's income. Off‐grid technologies have the potential to increase a household's income. The estimated average effect based on the random‐effects model was positive and statistically significant but very small ($\hat{\mathrm{SMD}}=0.06;95\%\;\mathrm{CI:}\;0.02\;\mathrm{to}\;0.10;\;p=0\text{.}01$). The level of heterogeneity in this analysis was moderate ($I^{2}=55.65\%$), and all but a couple of the estimates were assessed as having a high risk of bias (Figure 16).

There was no indication of outliers in the context of this model, and none of the studies were considered to be overly influential. Results did not vary by geographic region or country, nor did they differ based on the studies' year of publication. The intervention design features, such as technology type, mechanism, target population and scale, among others, were also not useful in explaining the heterogeneity level in this analysis. If we focus our analysis exclusively on estimates without a high risk of bias, two studies remain (Arraiz and Calero 2015 and Xiao et al. 2023). The overall weighted average effect decreases and is no longer statistically significant (${\hat{\mathrm{SMD}}}_{\mathrm{Low}\;\mathrm{or}\;\mathrm{med}\;\mathrm{ROB}}=0\text{.}05,\;95\%\;\mathrm{CI:}\;-0\text{.}07\;\mathrm{to}\;0\text{.}18;\;p=0\text{.}42$), but this result should be interpreted with caution given the small number of estimates contributing to this analysis.

###### 5.3.1.3.2 Allocation of Time

In this section, we synthesise quantitative effects for all outcomes measured in time units. Other than an increase in the time spent studying, there was no evidence of an effect of off‐grid technologies on the time spent in other activities, including remunerated or domestic work, leisure, or resting.

###### 5.3.1.3.3 Time Spent Working (Remunerated)

**Figure 17:**
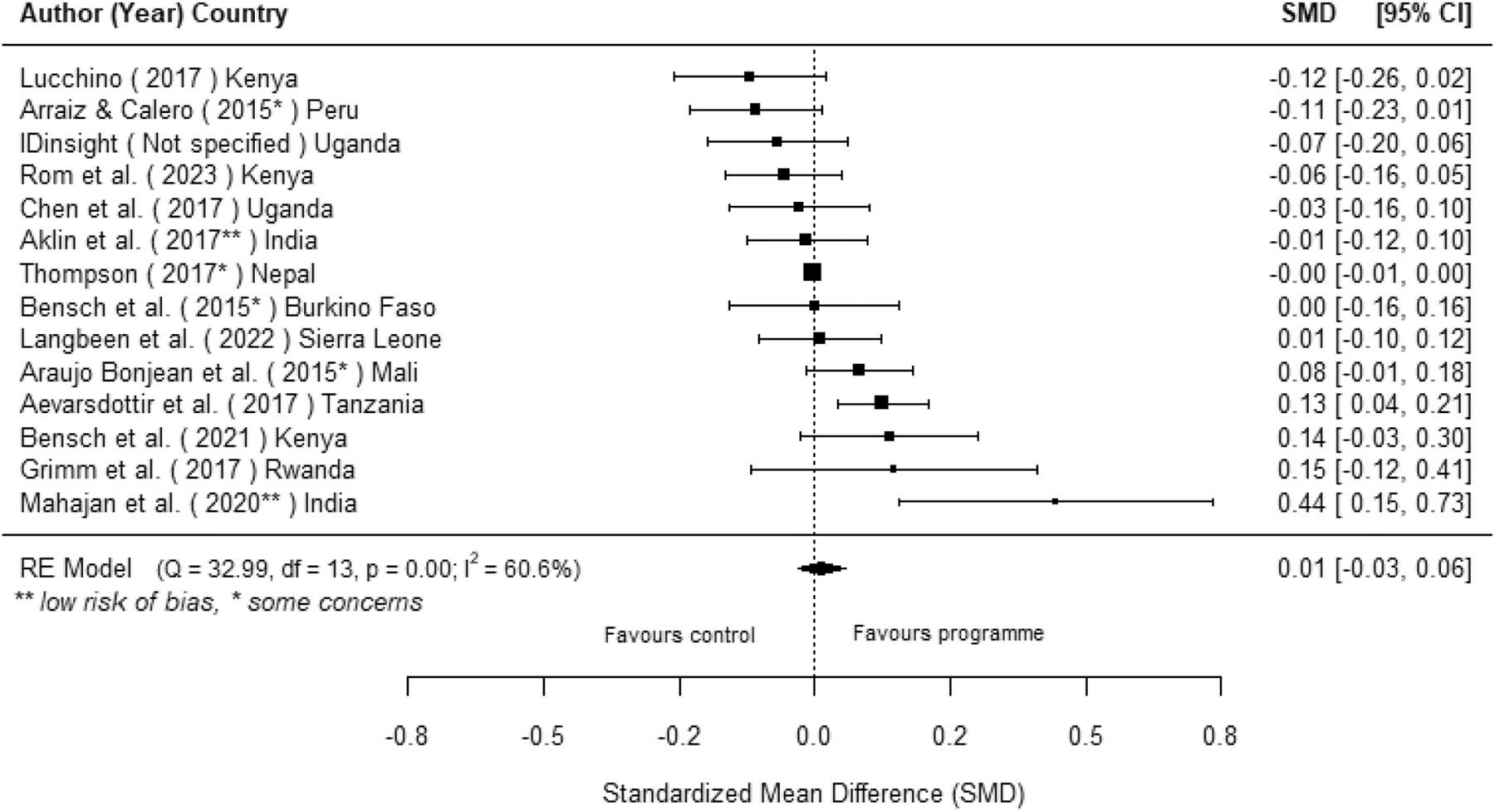
Forest plot showing estimates of the random‐effects model for the effect of off‐grid interventions on time spent working.

We included a total of 14 studies reporting the effects of off‐grid technologies on the time spent by individuals or households on income‐generating activities.^5^ We found no effect on time spent on remunerated work. The overall average effect based on the random‐effects model was very small and not statistically different from zero ($\hat{\mathrm{SMD}}=0.01;\;95\%\;\mathrm{CI:}\;-0.03\;\mathrm{to}\;0.06;\;p=0\text{.}58$). Estimates used in this analysis were moderately heterogeneous ($I^{2}=60.59\%$), and again, most estimates were rated as having a high risk of bias (Figure 17).

The type of off‐grid technology was the only moderator that helped explain differences in the results among studies. Effects from solar lamps with mobile phone chargers were significantly larger than the effects from other technologies (p<0.01). Solar lamps/lanterns with mobile charges increased the time spent working (${\hat{\mathrm{SMD}}}_{\mathrm{lamp}+\mathrm{charger}}=0.20;\;95\%\;\mathrm{CI:}\;0.03\;\mathrm{to}\;0.38;p=0\text{.}02;\;k=3$). There were no significant differences in the results based on the studies' risk of bias rating. Removing the eight studies rated as having a high risk of bias, the effect of off‐grid technologies on working hours was still not statistically different from zero (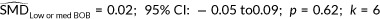).

None of the studies could be considered an outlier, nor did results change when we left one study at a time.

###### 5.3.1.3.4 Time Spent on Domestic Work

**Figure 18:**
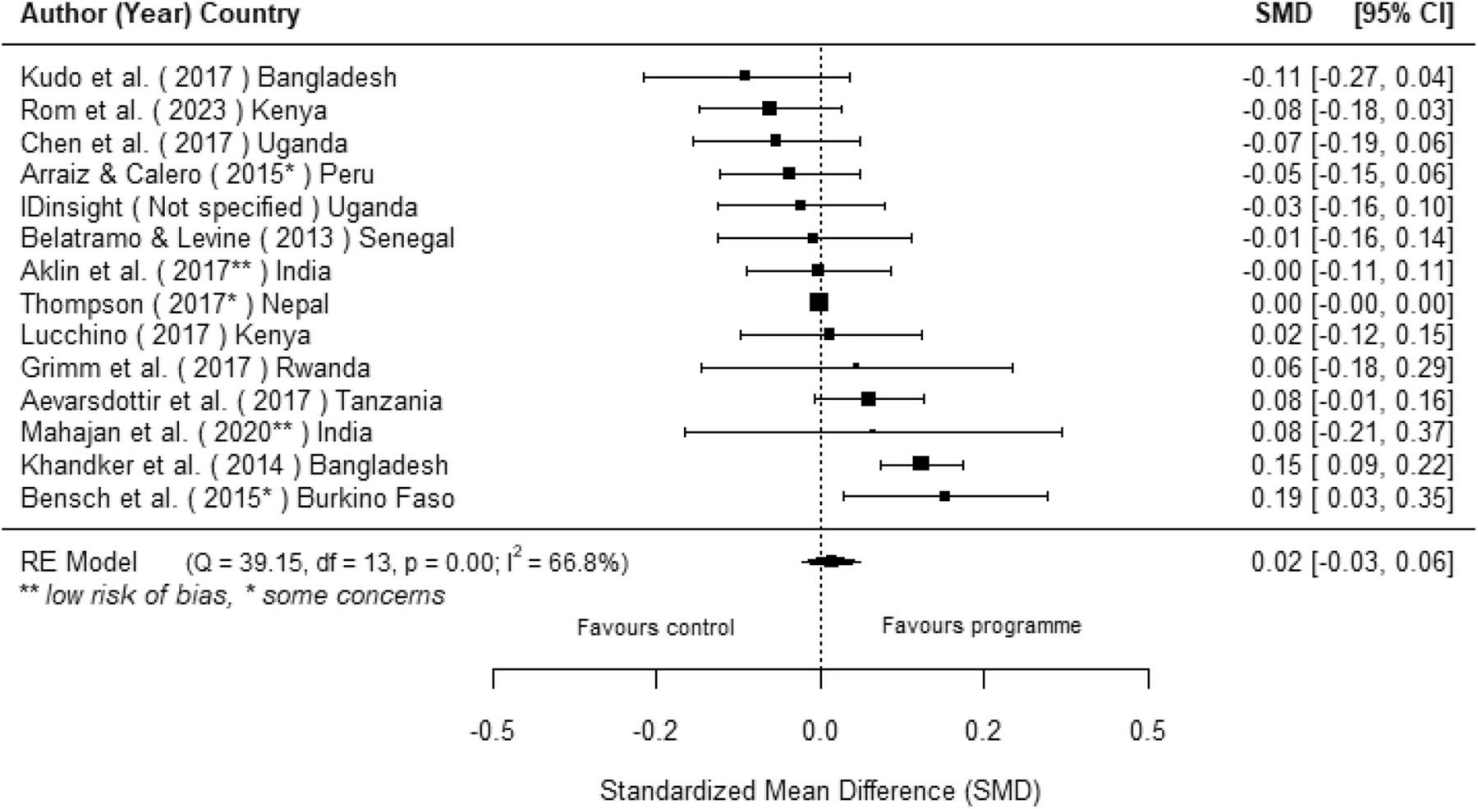
Forest plot showing estimates of the random‐effects model for the effect of off‐grid interventions on time spent on domestic work. We reversed the sign for time spent on domestic work as we consider the reduction in this measure to be positive.

Our quantitative synthesis is based on the effects of the overall time spent on domestic work reported in 14 studies, but we also identified studies reporting the effects on specific activities such as cooking (k=4), caretaking (k=2), and collecting fuel (k=3). The overall pooled effect on domestic work based on the random‐effects model was very small and not statistically significant ($\hat{\mathrm{SMD}}=0.02;\;95\%\;\mathrm{CI:}-0.03\;\mathrm{to}\;0.06;\;p=0.46$). The level of heterogeneity in this analysis was moderate ($I^{2}=66.79\%$), and only five studies were not rated as having a high risk of bias (Figure 18).

One study (Khandker et al. 2014) may be a potential outlier in the context of this model and could be considered overly influential. Removing this study did not change the overall result of no effect on the time spent on domestic work, as the average effect size was still very small and not statistically significant ($\hat{\mathrm{SMD}}=-0.001,\;p=0.96$).

Studies with a focus on the demand side (e.g., donating solar lamps with a mobile charger evaluated in Bensch et al. 2015) showed statistically less time spent on domestic work when compared to interventions focused on both supply and demand (e.g., training and awareness‐raising activities for staff, the POs and customers in addition to subsidised prices and access to credit evaluated by Khandker et al. 2014) (p<0.01). We found no statistical difference in results based on geographic region or country. Technology type, implementation mechanism and intervention scale did not help explain differences in results either. Study characteristics such as the year of publication and the risk of bias rating were not significant moderators either. Removing all effect sizes rated as having a high risk of bias does not change the overall result of no effect on the time spent on domestic work as the result is still not statistically different from zero (${\hat{\mathrm{SMD}}}_{\mathrm{Low}\;\mathrm{or}\;\mathrm{med}\;\mathrm{ROB}}=0\text{.}01,\;95\%\;\mathrm{CI:}\;-0\text{.}04\;\mathrm{to}\;0\text{.}07;\;p=0\text{.}63;\;k=5$).

###### 5.3.1.3.5 Time Spent on Leisure

**Figure 19:**
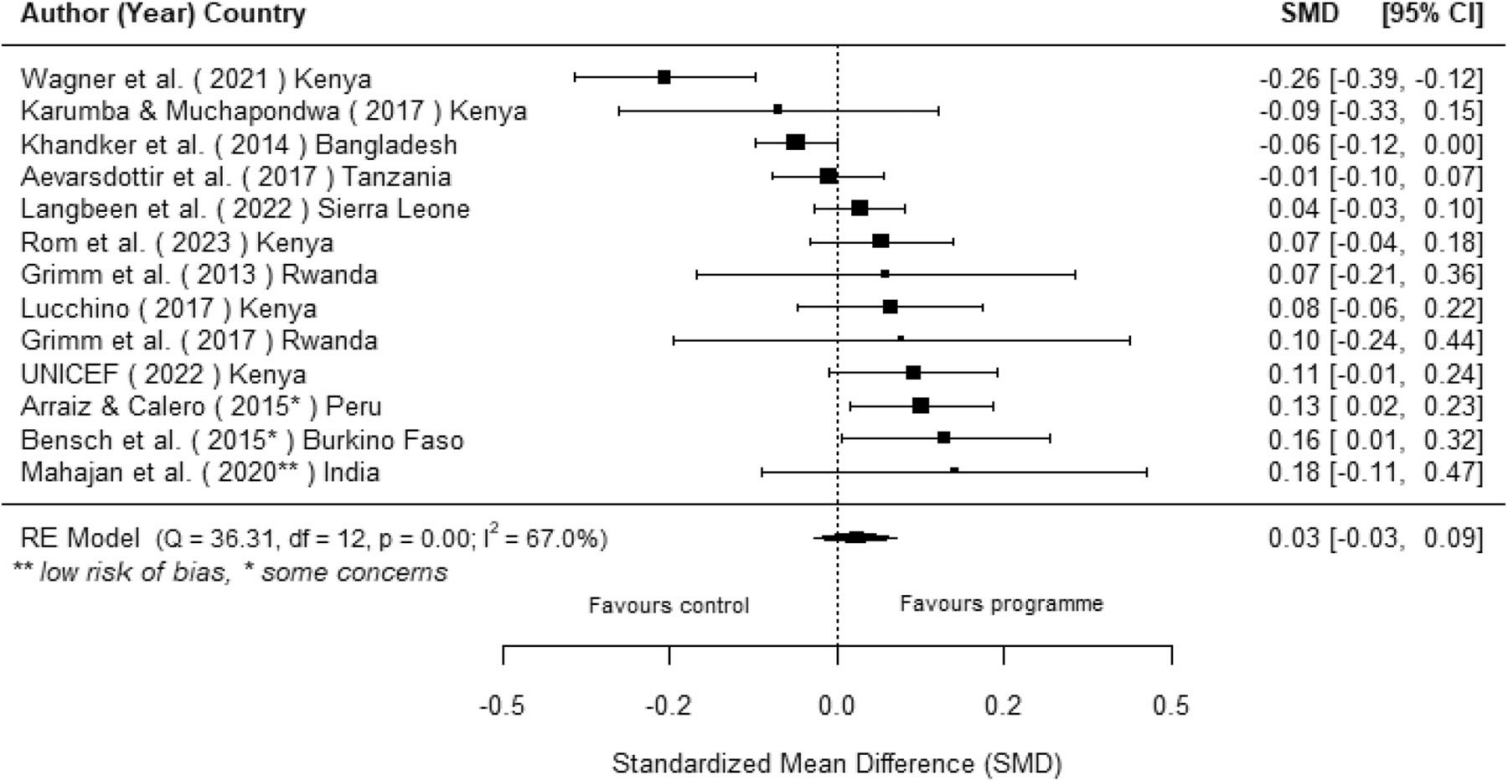
Forest plot showing estimates of the random‐effects model for the effect of off‐grid interventions on time spent on leisure.

Our quantitative synthesis is based on the effects for the overall time spent on leisure reported in 13 studies, but we also identified studies reporting the effects on specific activities such as watching TV (k=2), radio listening (k=3), reading (k=2) and playing (k=2). On average, interventions did not have a significant effect on time spent on leisure (e.g., socialising, praying, playing, reading or radio listening). The overall average effect based on the random‐effects model was very small and not statistically different from zero ($\hat{\mathrm{SMD}}=0\text{.}03;\;95\%\;\mathrm{CI:}\;-0\text{.}03\;\mathrm{to}\;0\text{.}09;\;p=0\text{.}36$). The level of heterogeneity in this analysis was moderate ($I^{2}=66.95\%$), and we rated most effects as having a high risk of bias (Figure 19).

One study (Wagner et al. 2021) may be considered a potential outlier. Removing this study increased the overall average effect on leisure time and made it marginally significant ($\hat{\mathrm{SMD}}=0.05,p=0\text{.}06$). Compared to national programmes, regional programmes showed a larger positive effect (p<0.01). Pooling the effects of the six studies with a regional scale indicated that off‐grid technologies implemented in a region significantly increase the time spent on leisure (${\hat{\mathrm{SMD}}}_{\mathrm{Regional}}=0.11;\;95\%\;\mathrm{CI:}0.05\;\mathrm{to}\;0.16;\;p<0.01$) and there was no heterogeneity for this analysis. Other contextual and intervention design characteristics did not help explain differences in the results. Compared to effects rated as having some concerns (Arraiz and Calero 2015; Bensch et al. 2015) or low risk of bias (Mahajan et al. 2020), the effects rated as having high risk of bias were smaller (p=0.04). The overall average effect excluding high‐risk of bias studies was small, positive and statistically significant (${\hat{\mathrm{SMD}}}_{\mathrm{Low}\;\mathrm{or}\;\mathrm{med}\;\mathrm{BOB}}=0\text{.}14;\;95\%\;\mathrm{CI:}\;0\text{.}06\;\mathrm{to}\;0\text{.}23;\;p<0\text{.}01;\;k=3$), and there was no heterogeneity in this analysis either. However, these results should be considered with caution given the small sample size used for it.

###### 5.3.1.3.6 Time Spent Resting/Sleeping

**Figure 20:**
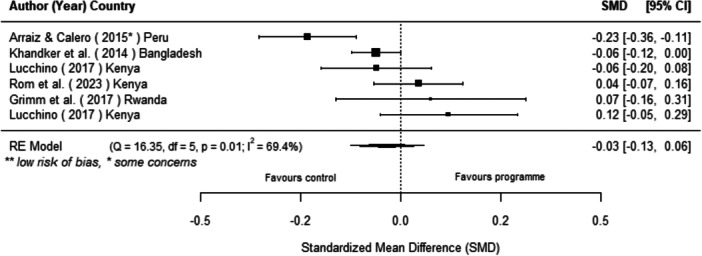
Forest plot showing estimates of the random‐effects model for the effect of off‐grid interventions on time spent resting or sleeping.

We included six studies in the analysis of the effect of off‐grid technologies on resting or sleeping and found a null overall effect. The overall average effect based on the random‐effects model was very small and not statistically different from zero ($\hat{\mathrm{SMD}}=-0\text{.}03;\;95\%\;\mathrm{CI:}\;-0\text{.}13\;\mathrm{to}\;0\text{.}06;\;p=0\text{.}47$). The level of heterogeneity in this analysis was moderate ($I^{2}=69.42\%$), and all but one of the included studies were rated as having a high risk of bias (Figure 20).

There was no indication of outliers influencing the overall average effect. However, significant results from moderator analysis are based on two groups of studies. On one side, four studies implemented in SSA by a non‐government agency used an experimental design. The other two studies evaluated interventions implemented by the government, used a QED and were not in SSA. The effects from quasi‐experimental studies were on average smaller than those from experimental studies in SSA, however, the pooled effect was still not statistically significant (${\hat{\mathrm{SMD}}}_{\mathrm{QED}}=-0\text{.}14;\;95\%\;\mathrm{CI:}\;-0\text{.}31\;\mathrm{to}\;0\text{.}03;\;p=0\text{.}10;k=2$). Other study and intervention characteristics were not significant moderators.

###### 5.3.1.3.7 Time Spent Studying

**Figure 21:**
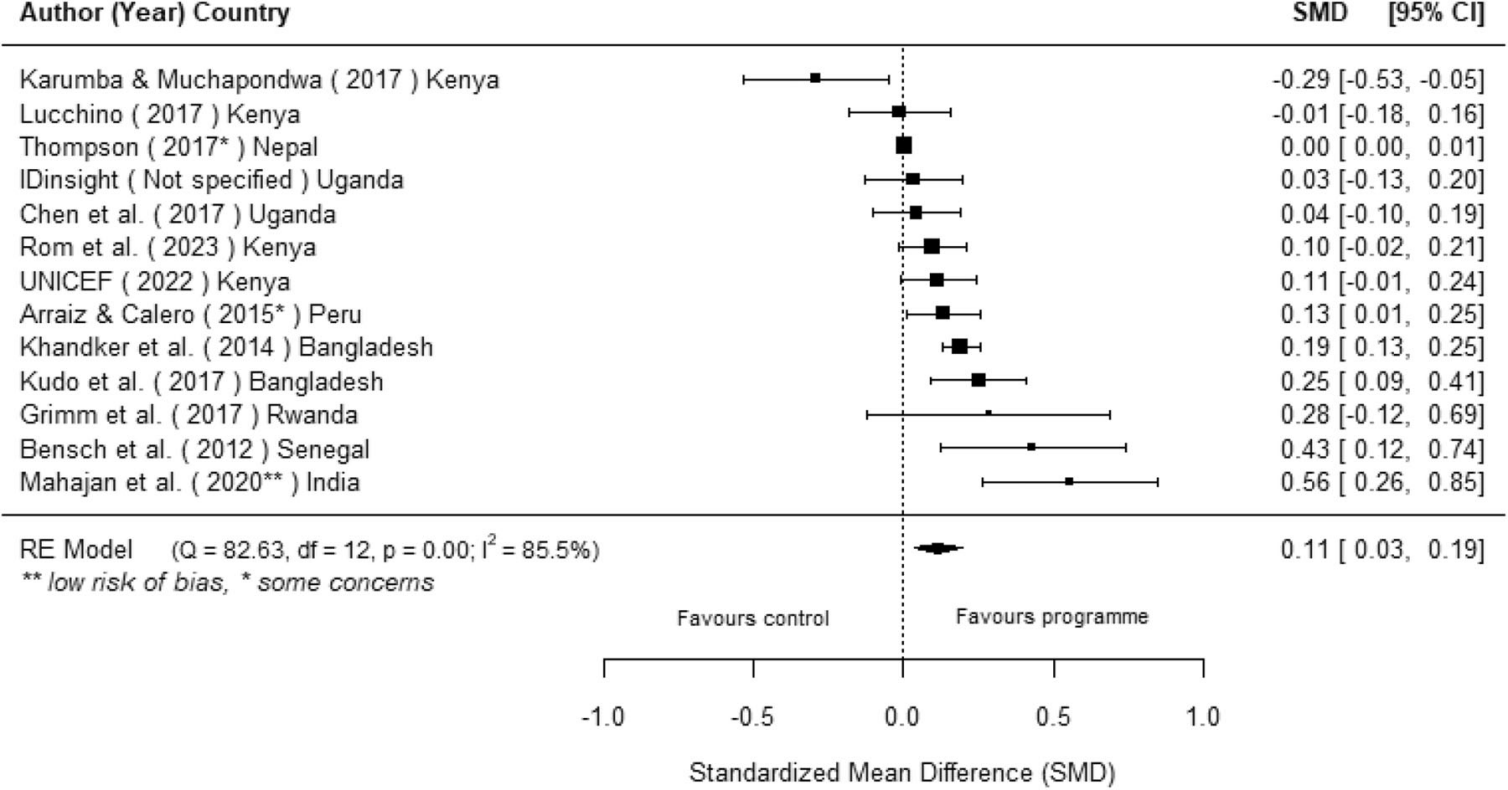
Forest plot showing estimates of the random‐effects model for the effect of off‐grid interventions on time spent studying.

We included 13 studies in the analysis of the effect on the number of hours a student spends studying, either captured in school or at home.^6^ Off‐grid technologies can increase the time spent studying. The overall average effect was moderate, positive and statistically significant ($\hat{\mathrm{SMD}}=0.11;\;95\%\;\mathrm{CI:}\;0.03\;\mathrm{to}\;0.19;\;p<0.01$). However, the level of heterogeneity in this analysis was large and significant ($I^{2}=85.48\%$), and only three studies were not rated as having a high risk of bias (Figure 21).

Factors that helped explain differences in the results included the type of technology and the intervention mechanism. Interventions using solar lamps/lanterns with mobile phone chargers showed a larger effect compared to the rest of the technologies (*p* < 0.01), while SHS showed a larger effect compared to micro‐hydro plants (*p* = 0.02). Interventions that provided the opportunity to access technology through market expansion showed a significantly smaller effect on time spent studying compared to those that directly provided the technology (*p* = 0.03).

When we rerun the analysis only for those studies where results were not rated as having a high risk of bias, the overall average effect on time studying is larger in magnitude but less precise and only marginally significant (${\hat{\mathrm{SMD}}}_{\mathrm{Low}\;\mathrm{or}\;\mathrm{med}\;\mathrm{BOB}}=0\text{.}17;\;95\%\mathrm{CI:}\;-0\text{.}03\;\mathrm{to}\;0\text{.}38;\;p=0\text{.}09;\;k=3$).

None of the studies were considered an outlier and the main result was not sensitive to leaving a study out one at a time. However, this was the only outcome in the review for which there is significant evidence of funnel plot asymmetry, suggesting an unbalanced representation of results in the published literature (t=2.77,df=11,p=0.02). The presence of publication bias indicates that the true effect size could be smaller, or not significant, than the pooled effect presented above.

###### 5.3.1.3.8 Time Spent Studying (Children Only)

**Figure 22:**
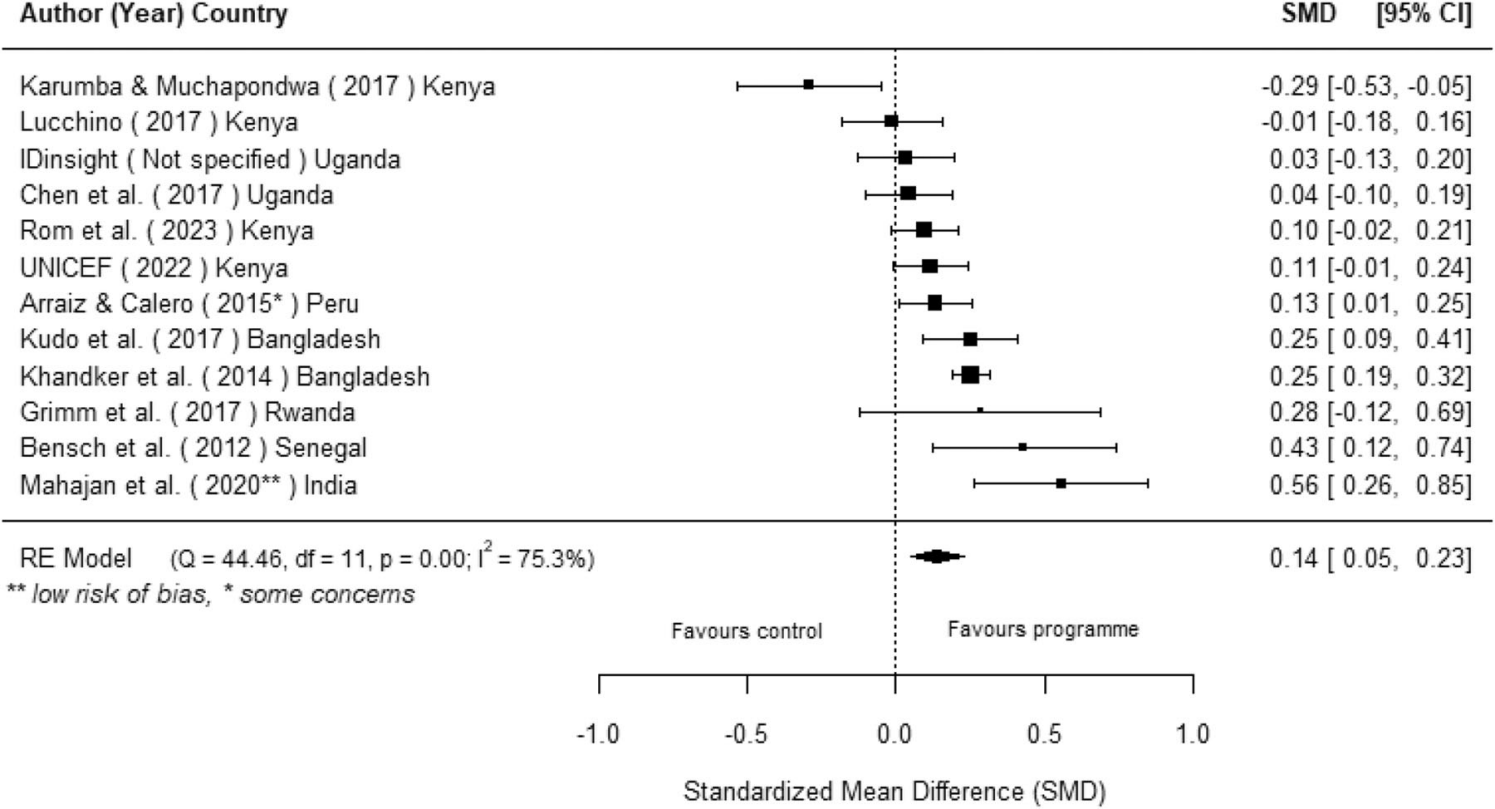
Forest plot showing estimates of the random‐effects model for the effect of off‐grid interventions on time spent studying by children only.

Interventions showed a positive and significant increase in time spent studying by children. We included 12 studies in this analysis. The estimated average effect based on the random‐effects model was small, positive and statistically significant ($\hat{\mathrm{SMD}}=0.14;\;95\%\;\mathrm{CI:}\;0.05\;\mathrm{to}\;0.23;\;p<0.01$). The true effects, however, appear to be heterogeneous ($I^{2}=75.26\%$) and all but two effect sizes were rated as having a high risk of bias (Figure 22).

Moderator analyses here showed similar results to those for the effects on time studying, regardless of age. Again, the effects found for solar lamps/lanterns with mobile phone chargers were on average larger compared to other technologies (p=0.03), while the opportunity to access or market expansion was a mechanism with significantly smaller effects on the time spent studying by children compared to studies that directly provided the technology (p=0.01).

When we removed effect sizes rated as having a high risk of bias from the analysis, the overall average effect was no longer significant (${\hat{\mathrm{SMD}}}_{\mathrm{Low}\;\mathrm{or}\;\mathrm{med}\;\mathrm{BOB}}=0\text{.}32;\;95\%\;\mathrm{CI:}\;-0\text{.}09\;\mathrm{to}\;0\text{.}73;\;p=0\text{.}13;\;k=2$). However, this result should be interpreted with caution, given the small sample size it is based on.

###### 5.3.1.3.9 Education

We assessed the effects of interventions on education using school attendance and test scores. We found positive, but not statistically significant effects for both attendance and test scores.

###### 5.3.1.3.10 School Attendance

**Figure 23:**
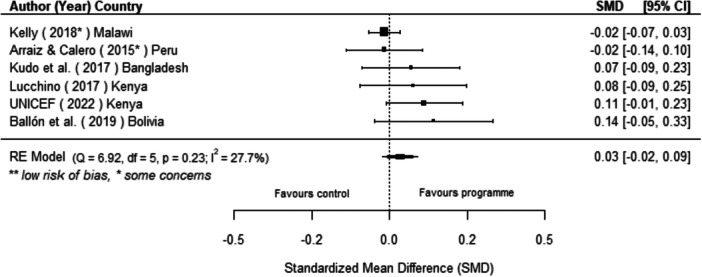
Forest plot showing estimates of the random‐effects model for the effect of off‐grid interventions on school attendance.

We found six studies looking at the effects on the time a student spends attending school, absenteeism ratios, percentage attendance, or the proportion of children regularly attending school. Included studies do not show a statistically significant effect on school attendance. The estimated average effect based on the random‐effects model was very small and not statistically different from zero ($\hat{\mathrm{SMD}}=0.03;\;95\%\;\mathrm{CI:}\;-0.02\;\mathrm{to}\;0.09;\;p=0.25$). There was no significant amount of heterogeneity in the true effects ($I^{2}=27.72\%$), but only two studies were not rated as having a high risk of bias (Figure 23).

From the moderators tested, we found no significant differences across groups. Compared to high risk of bias studies, those rated as having some concerns reported a marginally smaller effect (*p* = 0.01) so that the pooled effect becomes negative, but it is still very small and not statistically significant (${\hat{\mathrm{SMD}}}_{\mathrm{Low}\;\mathrm{or}\;\mathrm{med}\;\mathrm{BOB}}=-0\text{.}02;\;95\%\;\mathrm{CI:}\;-0\text{.}06\;\mathrm{to}\;0\text{.}03;\;p=0\text{.}48;\;k=2$). Finally, none of the studies were considered an outlier, nor were any of the effect sizes in the analysis overly influential.

###### 5.3.1.3.11 Test Scores

**Figure 24:**
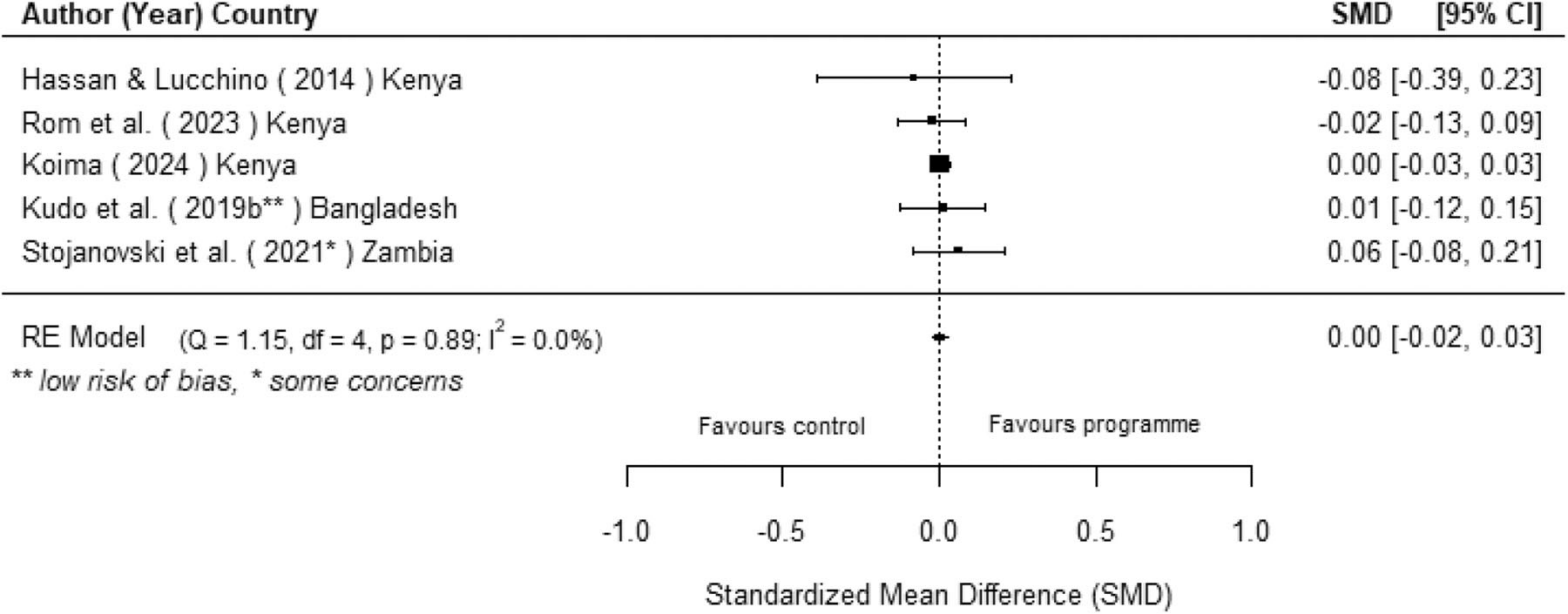
Forest plot showing estimates of the random‐effects model for the effect of off‐grid interventions on test scores.

We include the results from five studies in our synthesis of the effects of off‐grid interventions on test scores. Off‐grid technologies do not change the average test scores of students. The overall average effect based on the random‐effects model was very small and not statistically significant ($\hat{\mathrm{SMD}}=0.002;\;95\%\;\mathrm{CI:}\;-0.02\;\mathrm{to}\;0.03;\;p=0.86$). There was no significant amount of heterogeneity in this analysis ($I^{2}=0.00\%$), but most effect sizes used were rated as having a high risk of bias (Figure 24).

From the moderators tested, we found no significant differences across groups. Removing the effect sizes with a high risk of bias, the results did not change (${\hat{\mathrm{SMD}}}_{\mathrm{Low}\;\mathrm{or}\;\mathrm{med}\;\mathrm{BOB}}=0\text{.}04;\;95\%\;\mathrm{CI:}-0\text{.}06\;\mathrm{to}\;0\text{.}13;\;p=0\text{.}49;\;k=2$). None of the studies had a value larger than ± 2.576, and hence, there was no indication of outliers.

###### 5.3.1.3.12 Respiratory Illnesses

**Figure 25:**
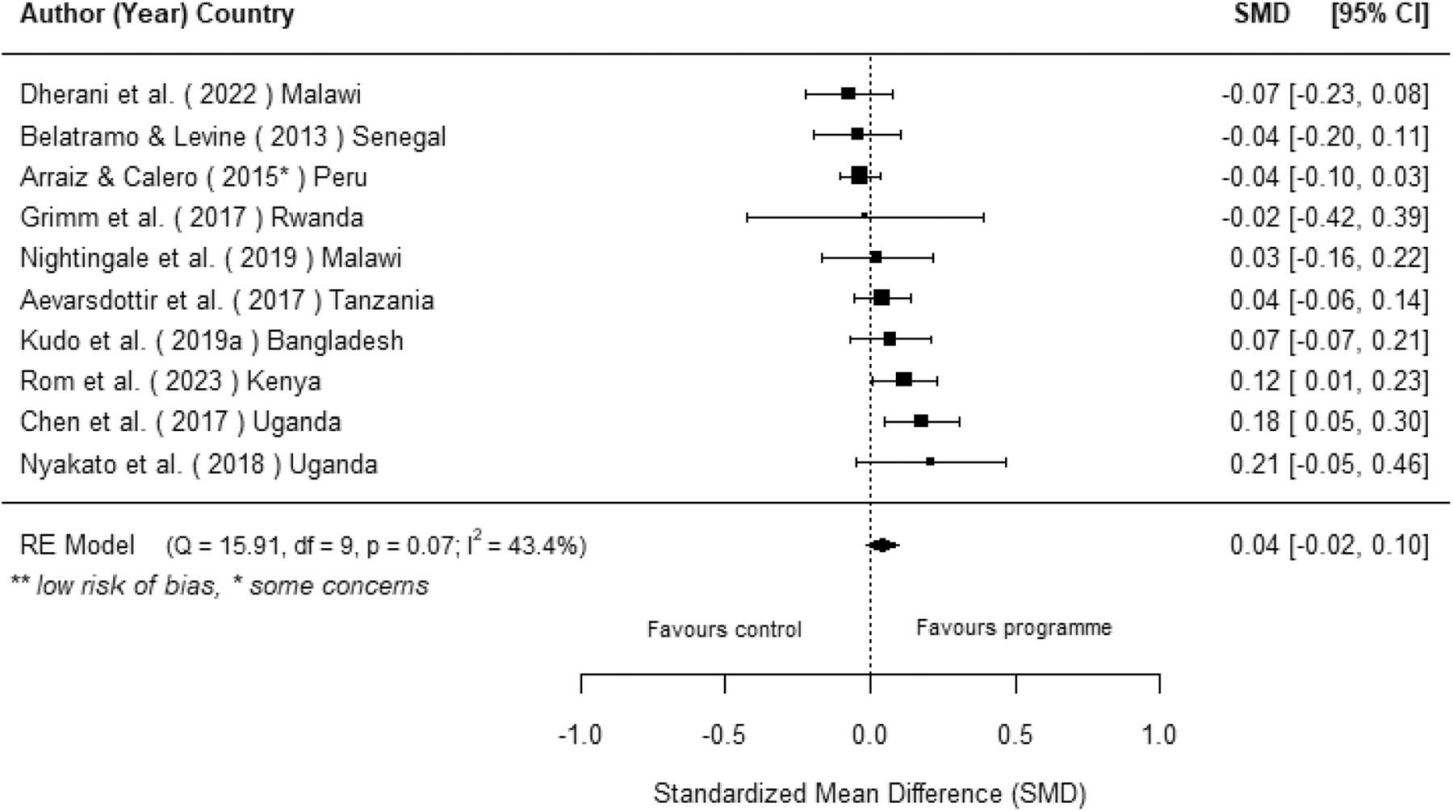
Forest plot showing estimates of the random‐effects model for the effect of off‐grid interventions on respiratory illnesses. We reversed the signs of effects on respiratory illness as we consider a reduction in this outcome to be positive.

Measures of respiratory illness reported in the 10 studies used in this analysis included indices and individual indicators of respiratory diseases or symptoms.^7^ We found no effect of off‐grid technologies on this outcome. The overall average effect size based on the random effects model was very small and not statistically different from zero ($\hat{\mathrm{SMD}}=0\text{.}04;\;95\%\;\mathrm{CI:}\;-0\text{.}02\;\mathrm{to}\;0\text{.}10;p=0\text{.}16$). We observed some heterogeneity in the effects used in this analysis ($I^{2}=43.43\%$). All but one estimate were rated as having a high risk of bias (Figure 25).

From the moderators tested, we found no significant differences across groups other than a larger effect on average for studies from Uganda (*p* = 0.02) Most studies in this analysis came from SSA, one from South Asia and one from Latin America. All interventions were demand‐focused, with varying mechanisms and scale, which did not help explain differences in results. There was only one study not rated as having a high risk of bias. None of the studies were an outlier or overly influential.

###### 5.3.1.3.13 Women's Empowerment

**Figure 26:**
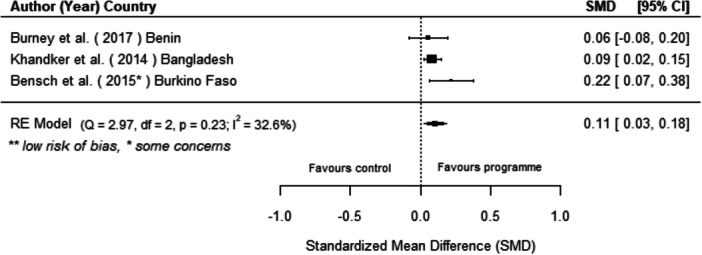
Forest plot showing estimates of the random‐effects model for the effect of off‐grid interventions on women's empowerment.

Based on three studies that reported comparable women's empowerment outcomes, we found a small statistically significant positive effect on women's decision‐making ($\hat{\mathrm{SMD}}=0\text{.}11;\;95\%\;\mathrm{CI:}\;0\text{.}03\;\mathrm{to}0\text{.}18;\;p<0\text{.}01$).^8^ Further, there was no significant amount of heterogeneity in this analysis ($I^{2}=32.61\%$). This finding should be taken with caution, given the small sample size for the analysis and the risk of bias rating of the included studies (Figure 26). This outcome only includes decision‐making indicators, and no other aspects of women's empowerment.

There was insufficient variation in the included studies to allow for moderator analyses. None of the studies could be considered an outlier nor were any of the effect sizes overly influential.

#### 5.3.2 Qualitative Synthesis

This section details the evidence we identified to answer review question 3. Here, we are interested in understanding which factors may or may not have impacted programme effectiveness, as opposed to understanding the impact of the included programmes from a qualitative lens. As discussed above, we conducted an additional search to identify documents to help answer this question. Our search led to the identification of 39 documents that provided qualitative and descriptive information related to barriers and facilitators of the effectiveness of the programmes included within our review. In addition, 30 of the included impact evaluations provided details on barriers and facilitators either through a dedicated qualitative analysis within a mixed‐method evaluation or through descriptions of programme implementation.

Our analysis is constructed around and builds upon the work of Sovacool (2013), which identified 10 themes that contributed to the success or failure of renewable energy access programmes in the Asia‐Pacific region. These are: appropriate technology; income generation; financing; political leadership; capacity building; programmatic flexibility; marketing and awareness; stakeholder engagement; community ownership; technical standardisation. Our analysis finds that these themes are pertinent to contexts beyond the Asia‐Pacific region, in addition to other factors, such as the impact of COVID‐19 and societal norms, that can also act as further barriers off‐grid interventions face. Given that we are unable to make causal claims from the documents identified for this analysis, the results here should be interpreted with caution and viewed as suggestive only.

##### 5.3.2.1 Process and Quality of the Qualitative Evidence

Having identified 69 documents with relevant information on barriers and facilitators, we began by grouping these documents based on their study design. From these documents, we identified 19 data‐based qualitative or mixed‐methods papers, 7 of which included impact evaluations. As these papers utilised qualitative data analysis techniques, it was possible to conduct a critical appraisal to provide a sense of the trustworthiness of the findings based on their research design, sample and analysis approach. The tool used to appraise these studies critically is available in Appendix E, while the full appraisal results can be found in Appendix H.2.

After our appraisal, we scored three studies as high quality. These studies utilised defensible research designs, appropriate samples, were rigorous in conduct and provided contextualised and credible claims. Though they did provide some reflexivity in the researcher's own bias and positionality, these aspects could have been developed further. Nine studies were deemed of moderate quality, which lacked reflexivity but also suffered from other issues, mainly in the rigour of their methods and the credibility of their findings. Three studies were rated as low quality, which further suffered in regard to the research designs and samples used. The four studies that scored critical quality contained major flaws in regard to their sample, rigour and contextualisation and, as such, have not been included in our barriers and facilitators analysis.

Among these 19 papers, 13 were mixed‐methods studies. We also appraised their approach to integrating quantitative and qualitative findings. Ten studies were able to provide a relevant rationale for combining quantitative and qualitative studies, while those that did not made no attempt to justify why quantitative and qualitative findings were combined. All mixed‐methods papers provided a rationale for combining different types of data and provided evidence that this combination led to the emergence of new findings. However, fewer than half were transparent and rigorous in their methods for combining different types of data and even fewer discussed the limitations of their integration.

Aside from the qualitative and mixed‐methods papers, we were also able to identify barriers and facilitators in other documents. Additional barriers and facilitators data were available in 23 of our included impact evaluations. Though these evaluations did not utilise mixed‐methods, they did provide anecdotal notes on programme implementation while discussing the setup of interventions and in their conclusions. We were also able to identify five descriptive quantitative studies that did not utilise an IE method but provided information pertinent to the barriers and facilitators of our included studies. Three project documents did not focus on evaluating the impacts of programmes but instead provided descriptive details about their implementation. We also identified six literature reviews that provided an overview of an included off‐grid technology programme.

There is a large variation between the studies identified for the barriers and facilitators analysis. While project documents or evidence from impact evaluations of small‐scale programmes may provide details across the entirety of a programme's implementation area and timeline, studies of large‐scale national programmes, such as China's PVPA programme, usually focus only on certain areas at specific points in time. While qualitative and mixed‐methods papers attempted to account for this with the contextualisation of their results, barriers and facilitator data from other evaluations of large‐scale programmes may only reflect issues for certain regions and areas. This, alongside the few high and moderate‐quality studies identified for this analysis, should caution the interpretation of the results.

Our analysis section overviews the findings from the high, moderate and low quality qualitative and mixed‐methods papers for each of the 10 themes highlighted by Sovacool (2013). We also highlight any other barriers, facilitators and unintended consequences from these studies. Only when information is scarce from qualitative and mixed‐methods papers studies do we refer to the findings from other documentation types (impact evaluations, project documents, literature reviews, descriptive quantitative papers). Additionally, Table 9 and Table 10 summarise each barrier and facilitator theme with example quotes.

###### 5.3.2.1.1 Appropriate Technology

Ensuring that the technology provided in programmes is suitable in local contexts can become a prerequisite to intervention effectiveness. Issues with inappropriate technology were highlighted as barriers in five papers. Two papers noted that microgrids were unable to provide adequate energy to communities in Sierra Leone (Etienne and Robert 2024) and Senegal (Levine et al. 2022). Within the Rural Electrification Senegal (ERSEN) programme, participating households had taken to purchasing additional solar products (such as lamps) to compensate for inadequate power supply. Aside from microgrids, specific instances of inappropriate technologies were also highlighted. Urmee and Harries (2011) found that SHS systems provided under the Bangladesh SHS programmes were not able to support the number of lamps required, Grimm et al. (2013) found that pico‐PV systems provided in Rwanda did not have the correct mobile phone adapter and Cundale et al. (2017) found that participants in a cookstove programme in Malawi did not allow their children to use the stoves due to the risk of them catching on fire.

Only two papers provided evidence that the choice of an appropriate technology was a facilitator of intervention effectiveness. Cundale et al. (2017) highlighted that over three‐quarters of respondents in their survey found the cookstove technology deployed to have no disadvantages, while Bensch et al. (2015) noted that almost all village representatives in the Yeelan Ba project in Burkina Faso identified the SHS as appropriate, especially given that grid‐electrification was unlikely to reach the rural areas of the programme.

###### 5.3.2.1.2 Income Generation

Coupling off‐grid interventions with income‐generating activities may lead to greater programme success, yet three papers highlighted barriers to income‐generating activities from both the implementer and participant sides. Two programmes have noted issues in receiving payments for services provided. In the ERSEN programme in Senegal, though most of the initial costs for setting up microgrids were covered by donors, the grids themselves were supposed to operate financially autonomously through the collection of household energy bills. Despite this, there were significant challenges with collecting household bills, leading to some households being cut‐off and impacting the microgrid business model (Etienne and Robert 2024). Kenya's Mwangaza Mashinani project was designed to piggyback an ongoing cash transfer programme, Inua Jamii, to allow households to pay for the solar supplies they rented. Due to payment delays with the cash transfer, often households were late on repayment or missed it altogether. Further issues were related to collecting revenue from older, vulnerable and rural households, which agents struggled to reach (UNICEF 2022). In China's PVPA programme, communities were able to financially benefit from the PV systems and spend funds on community enhancement. Yet, many households felt the financial benefits of the programme were not large enough, and some village leaders had their requests to increase the number of PV stations denied (Lo 2021).

Five studies found that integrating income‐generating activities can further the effectiveness and use of off‐grid technologies. Two programmes incorporated microcredit/franchises into their intervention to support the sustainability of their programmes. In Peru, micro‐franchises were set up to supply operation and maintenance to SHS as well as sell other solar products (Eras‐Almeida et al. 2019). In the Bangladeshi SHS programme, incorporating micro‐credit allowed the programme to move away from a pure reliance on cash sales and open the market to a greater number of households and small businesses (Urmee and Harries 2011). In China's PVPA programme, participants used solar project profits to develop community infrastructure, improving the economic and environmental conditions of the community (Chen et al. 2021; Wang et al. 2023). While there were delays to cash transfers during the Mwangaza Mashinani project in Kenya, its alignment with the larger cash transfer programme reduced transaction costs and sought to ensure that top‐up payments were used for their intended purposes (UNICEF 2022).

###### 5.3.2.1.3 Financing

Off‐grid technologies can be costly for users, and as such, many interventions aim to overcome this financial hurdle. Despite this, the costs of these technologies and their operation can still pose a significant barrier to intervention effectiveness, as highlighted in eight papers. Even with the provision of subsidies, the cost of the initial technology adoption, whether it be a cookstove (Cundale et al. 2017) or a solar PV system (Chen et al. 2021), may still be too high for households to afford, which may further limit the expansion of off‐grid services provided (UNICEF 2022). When technologies were provided directly to participants in Rwanda, these were sold to help ease financial hardship, even after having signed contracts to prevent this practice (Grimm et al. 2013). Entrepreneurs and partner organisations who were provided with support to expand energy markets struggled to overcome the upfront cost of setting up their operations, and without recovering loan amounts, they were unable to expand their services in Kenya and Bangladesh (Bensch et al. 2016; Urmee and Harries 2011). Beyond the initial costs of the technology, two papers also found that operational fees in Sierra Leone (Levine et al. 2022) and fees for loaning the technologies in Burkina Faso (Bensch et al. 2015) led to complaints from participants who found the fees too high, and in the case of the loans, were unable to understand why they were paying such a high price for technology that they did not own.

Two papers did, however, highlight financing as a facilitator. Unlike Chen et al. (2021), Wang et al. (2023) found that the subsidised cost of solar PV as part of the PVPA programme in China led to participants installing systems, as it was now affordable to them. Eras‐Almeida et al. (2019) discussed how the Acciona programme in Peru conducted a pre‐intervention analysis of household energy costs to ensure that the tariff for solar energy matched what participants were previously paying for conventional energy.

###### 5.3.2.1.4 Political Leadership

The lack of government support and/or inadequate policy frameworks can limit the effectiveness of sustainable energy interventions, as supported by two papers. Urmee and Harries (2011) highlighted the case of Bangladesh's SHS programme, describing that despite the enactment of a national renewable energy policy in 2008, government investments in supporting rural electrification projects had been minimal. They pointed out that the absence of special incentives and tax provisions limited private sector involvement in rural electrification. This lack of support reduces motivation to invest in rural electrification projects, limiting the progress of sustainable energy solutions in rural areas. In the case of China's PVPA programme, Lo (2021) discussed that local village leaders had no influence over critical decisions of the PV stations, which were determined by higher‐level governments. The Chinese government's top‐down approach, justified by officials as necessary due to the technical nature of PV technology, may overlook community needs, thus limiting the project's benefits.

On the other hand, four papers found that political leadership, through supportive policies and strategic alignment with national goals, can facilitate the successful implementation of sustainable energy interventions. In Kenya, the alignment of the Mwangaza Mashinani project with the social protection sector's priorities illustrates how integrating energy access initiatives with broader poverty reduction and welfare improvement objectives can enhance their impact (UNICEF 2022). Similarly, in Peru, strong political support at all levels contributed to building community trust and ensured the implementation and longevity of off‐grid interventions (Eras‐Almeida et al. 2019). Moreover, two papers highlighted that within China, trust in the government led to greater levels of community participation and confidence in the sustainability of the PVPA programme (Chen et al. 2021; Wang et al. 2023).

###### 5.3.2.1.5 Capacity Building

Building the capacity of local actors to enact and manage off‐grid programmes can lead to their long‐term sustainability, yet two papers found significant barriers related to capacity building. Urmee and Harries (2011) observed that the SHS programme in Bangladesh faced growth constraints due to a lack of managerial capacity. Programme implementers deemed it necessary to increase the supply of individuals capable of managing programmes, requiring training in technical and managerial skills. However, the lack of incentives to attract and retain high‐quality staff was another related challenge. On the issue of retaining staff, Bahaj et al. (2019) noted that after 1 year of operation of the electrical mini‐grid project in Kenya, a technician started his own business, and some trained managers also moved on to other jobs in the sub‐location. This highlighted the challenge of retaining staff who leverage their new skills for better opportunities elsewhere.

Three programmes found that when capacity building is embedded into programmes, this can facilitate positive programme effects. In the Bangladeshi SHS programme, building customers' capacity to maintain the system was reported to help reduce misuse and ultimately lower maintenance costs (Urmee and Harries 2011). Similarly, in rural communities in Peru, enhancing local people's capacity for SHS maintenance was identified as a key factor in reducing system error rates and positively impacting operation and maintenance costs (Eras‐Almeida et al. 2019). In Kenya, Bahaj et al. (2019) described a well‐established ongoing support system for local capacity in mini‐grid operation and maintenance, which can support intervention sustainability.

###### 5.3.2.1.6 Programmatic Flexibility

During the implementation of any programme, unexpected issues may arise, and the flexibility of implementers to address them may support the effectiveness of the programmes. No studies identified programmatic flexibility as a barrier. However, one descriptive quantitative study noted that in the case of China's PVPA programme, flexibility may be associated with greater impact, given the scale of the country and the socio‐economic and environmental conditions of specific counties (Liao and Fei 2019).

Four studies highlighted programmatic flexibility as a facilitator of effectiveness. In two programmes, payment structures were altered to suit local contexts. In Kenya, microgrid bill payments were not collected on time despite pressure from local managers and the community. In response, the project changed to a pre‐payment metering system, which resolved the non/late‐payment problem, with 97% of bills being paid on time (Bahaj et al. 2019). In Sierra Leone, mini‐grid operators initially utilised two payment systems before moving to a monthly tariff, which was seen as a more viable option for the local context (Levine et al. 2022). In Peru, the Acciona programme was able to identify that the SHS faced failures, leading to intervention communities losing confidence. In response, the programme pivoted to an updated system, which had fewer failures (Eras‐Almeida et al. 2019). In Bangladesh, the strategic use of donor funds to provide short‐term loans to programme operators, rather than direct subsidies, aimed to create a sustainable SHS market (Urmee and Harries 2011).

###### 5.3.2.1.7 Marketing and Awareness

Whether and how information reaches the target population can be a crucial element for the success or failure of sustainable energy interventions. Two papers identified barriers to providing programme information. Marketing and awareness‐raising campaigns may face significant barriers due to scattered and isolated populations, encountering high costs when promoting projects where the target populations are dispersed and difficult to reach, as was the case for the Acciona programme (Eras‐Almeida et al. 2019). In Kenya, the implementers of the Mwangaza Mashinani project faced difficulties in providing adequate information and ensuring participants' understanding of key aspects of the project (UNICEF 2022). The lack of established communication systems created challenges across the delivery chain, affecting project targeting, enrolment, payment and repayment processes. In addition, the Mwangaza Mashinani project struggled to communicate technological information to participants due to low technology literacy, compromising registration and implementation processes (UNICEF 2022).

When marketing and awareness‐raising strategies are enacted effectively, it can support the uptake and use of technologies, as highlighted in five papers. Despite issues with communicating technological information, the Mwangaza Mashinani project in Kenya underlined the importance of in‐person meetings with village leaders and beneficiary welfare committee members for detailed information exchange on the programme as a whole (UNICEF 2022). In Burkina Faso, regular visits by Yeelen Ba technicians and the proximity of programme agencies to villages enhanced community engagement (Bensch et al. 2015). China's PVPA programme benefited from continuous communication of village leaders with residents as well as other forms of personal interactions (e.g., participants observing neighbours), which increased the willingness to adopt PV technology (Chen et al. 2021; Wang et al. 2023). In addition, programme officers in Bangladesh's SHS programme emphasised the critical relevance of raising awareness and strengthening market demand among potential users to facilitate programme expansion (Urmee and Harries 2011).

###### 5.3.2.1.8 Stakeholder Engagement

Low or lack of stakeholder engagement in the decision‐making and implementation processes of interventions was identified as a barrier to programme effectiveness in three papers. In the Mwangaza Mashinani project in Kenya, some stakeholders felt that they were informed but not actively involved during the project conceptualisation and design phases (UNICEF 2022). This lack of engagement persisted into the implementation phase, where only a few stakeholders and the implementing consortium were actively engaged. In China's PVPA programme, Lo (2021) observed that despite evidence of public consultation, crucial decisions, such as the size of the PV station and financial models, were made by county governments without input from local villagers. This lack of local involvement may have limited the potential benefits of the initiative. Urmee and Harries (2011) highlighted the need for better information sharing and networking among programme implementers to improve the issues around the component selection and approval process, as well as quality control, in the Bangladeshi SHS programme.

In four papers, active stakeholder engagement and strong partnerships were highlighted as facilitators. In Kenya, the Mwangaza Mashinani programme benefited from significant stakeholder input. Both technology suppliers, d.light and Bright Sky Solar, showed interest in expanding their products, as they were encouraged by the social impact of the programme and the de‐risking measures for suppliers. Technical working groups at the county level were also well‐attended and active, as highlighted by stakeholders (UNICEF 2022). In Sierra Leone, stakeholders, including the Ministry of Energy and mini‐grid operators, noted that the initiation of the United Nations Office for Project Services (UNOPS) was crucial for project startup and that their leadership was effective and necessary (Levine et al. 2022). In China, though some issues with a top‐down approach to the PVPA programme have been previously noted, the many opportunities for stakeholder engagement through village assemblies and representatives have also been highlighted as facilitators (Lo 2021). Eras‐Almeida et al. (2019) emphasised that strong partnerships among promoters, the public sector, funders, and communities, as well as involving local companies in leadership roles, increased trust in the SHS in Peru. The project implementing company utilised local resources, maintained close relationships with local people, and collaborated closely with the government to improve regulations, enhancing solar technology accessibility for people living in rural areas.

###### 5.3.2.1.9 Community Ownership

Creating a sense of community ownership is crucial for sustainable energy interventions. However, three papers identified barriers that can undermine this. In the Senegalese ERSEN programme, the village management committee, created to collect payments and resolve conflicts, ceased to function due to tensions over payment delays, electricity distribution and equipment maintenance (Etienne and Robert 2024). In Bangladesh's SHS programme, beneficiaries did not view themselves as the technology owners until the equipment was fully paid off, leading to poor maintenance practices (Urmee and Harries 2011). In addition, deviations from the enrolment process, such as waiving the commitment fee or having it paid by someone other than the beneficiary, undermined the sense of ownership in Kenya's Mwangaza Mashinani project (UNICEF 2022).

On the other hand, three papers highlighted the positive influence of community ownership. The Acciona programme in Peru created a sense of ownership by relying on local stakeholders familiar with the community's culture and language to play key roles in service delivery, sales and funding (Eras‐Almeida et al. 2019). In Peru, rural electrification committees included at least one woman and acted as intermediaries between operators and beneficiaries, collecting fees and assisting with maintenance, while also promoting gender equality (Eras‐Almeida et al. 2019). Similarly, the Mwangaza Mashinani project in Kenya embedded its structures within communities by establishing village committees and ‘community champions’, which facilitated maintenance and knowledge sharing (UNICEF 2022). Lastly, also in Kenya, a mini‐grid programme included cooperative memberships. Consumer and public pressure to lower tariff prices was communicated to these cooperatives, who reduced tariffs. This boosted market demand and reflected the cooperative's responsiveness to community needs (Bahaj et al. 2019).

###### 5.3.2.1.10 Technical Standardisation

Technical standardisation can pose a significant barrier to the success of sustainable energy interventions, particularly in terms of system/equipment maintenance and support services, as highlighted by three papers. In the CAPS project in Malawi, participants expressed concerns about the maintenance of cookstoves (e.g., difficulties in finding replacement parts and repair services), leading them to revert to traditional three‐stone fires (Cundale et al. 2017). Similarly, participants in China's PVPA programme reported insufficient follow‐up maintenance services, such as challenges locating appropriate contacts for system/equipment repair (Wang et al. 2023). Grimm et al. (2013) stressed the critical role of an after‐sales network, which in the case of the Pico‐PV intervention in Rwanda was practically non‐existent. Despite being stated in the programme proposal, the authors' follow‐up survey revealed a lack of trained agents and non‐functional technical support, which reduced customers' motivation to pay for the higher price technology and allowed the spreading of negative rumours about the technology.

Effective technical standardisation through system maintenance support and the availability of spare components were identified as key facilitators in one paper. Bangladesh's SHS programme covered systems maintenance during the microcredit loan period (3–4 years), which programme implementers viewed as crucial for increasing participants' SHS adoption and enhancing the programme's overall sustainability (Urmee and Harries 2011). In this perspective, implementers saw this programme component as risk mitigation of poor system performance, which could otherwise lead to a loss of faith in the SHS and damage the programme's reputation (Urmee and Harries 2011).

**Table 9:** Examples of barriers identified.

Theme and programmes reporting this finding	Evidence example	Papers contributing to this finding
Appropriate Technology	‘Moreover, in spite of the presentation of the ToughStuff kit in the proposal as being a field‐tested and appropriate technology designed for the needs of African households, the kit does not seem to be mature for widespread marketing. The power pack was not working in many cases and the mobile phone charger marketed during the first months (and thus also used for the RCT) was not adapted to the needs in the Rwandan market’. (Grimm et al. 2013, 47)	Etienne and Robert (2024); Levine et al. (2022); Cundale et al. (2017); Grimm et al. (2013); Urmee and Harries (2011)
Income Generation	‘The business model relies on the payment of a flat rate by connected households … However, a few months after connection, several households had trouble paying the monthly fee. The operator cut off one household in 2015 due to non‐payment … Ultimately, economic difficulties diminish households' energy budget and impact the MG business model’. (Etienne and Robert 2024, 6–7)	Etienne and Robert (2024); UNICEF (2022); Lo (2021)
Financing	‘Others indicated that although they were willing to participate, the initial installation cost was still too high for them. For instance, in Hong'an County (Group C), the initial investment required for each village power station was 24,000 RMB; even though this sum is split among the government, enterprises, and village households, the initial investment for each household was still substantial, about 8000 RMB’. (Lo 2021, 7)	Levine et al. (2022); UNICEF (2022); Chen et al. (2021); Cundale et al. (2017); Bensch et al. (2016); Bensch et al. (2015); Grimm et al. (2013); Urmee and Harries (2011)
Political Leadership	‘An example of the lack of a supporting government framework cited by POs was the introduction in 2007 of a 5% import duty solar modules that further increased the costs of the solar systems POs offered their customers. The lack of special incentives and taxation provisions such as those used to support similar rural electrification programs in India [17] were considered to be the reason for the low level of involvement of private financial institutions in the programs in Bangladesh’. (Urmee and Harries 2011, 2827–2828)	Lo (2021); Urmee and Harries (2011)
Capacity Building	‘After one year of project operation, one of the technicians started his own business near the trading center, selling electrical goods and services. This highlights the difficulty in retaining staff who are trained as part of a mini‐grid project and then—quite rightly—use their new skills to obtain improved employment opportunities elsewhere. The project also trained three managers, some of whom have moved on to other jobs in the sub‐location’. (Bahaj et al. 2019, 2828)	Bahaj et al. (2019); Urmee and Harries (2011)
Programmatic Flexibility	No qualitative or mixed‐method paper provided evidence on programmatic flexibility as a barrier to intervention effectiveness.	
Marketing and Awareness	‘Our findings show that appropriate communication systems were not adequately set up at the start of the project, leading to challenges across the delivery chain. In relation to targeting and enrolment, chiefs reported that they received limited information about the project enrolment process, and this lack of clarity around eligibility and enrolment filtered down to the CCs, BWCs, and households themselves’. (UNICEF 2022, 24)	UNICEF (2022); Eras‐Almeida et al. (2019)
Stakeholder Engagement	‘During the conceptualisation and design phase, most stakeholders (in particular, the MoE and KOSAP) felt that they were informed about the pilot project but were not actively involved in the design. Similarly, the solar suppliers stated that they were only involved in the project after the project had been designed and once they were selected, after responding to the request for proposals’. (UNICEF 2022, 34)	UNICEF (2022); Lo (2021); Urmee and Harries (2011)
Community Ownership	‘This committee ceased to function after less than a year due to the lack of commitment of its members, according to the village chief … A second aspect, related to bill collection, also contributed to the weakening of the management committee. Initially, this committee oversaw the collection of fees and handed them to the operator. This responsibility was later transferred to the village chief, who abandoned this task due to tensions with the operator over payment delays … Tensions also arose regarding electricity management’. (Etienne and Robert 2024, 7)	Etienne and Robert (2024); UNICEF (2022); Urmee and Harries (2011)
Technical Standardisation	‘Those that did find problems with the stove were largely concerned about the future maintenance of the stove once CAPS finished and damage to the pots. Concerns were raised over where to get replacement parts and where to go for repairs’. (Cundale et al. 2017, 5)	Wang et al. (2023); Cundale et al. (2017); Grimm et al. (2013)
Other Barriers	‘The qualitative analysis suggests that mobility seems to be a particular challenge for women in the solar business. In order to expand the solar business, it is often necessary to extend one's outreach. This, however, involves travelling and even over‐night stays. It was mentioned that this is a particular problem for women, as husbands might not allow such over‐night stays and are hesitant about travelling in general. According to the qualitative interviews, the notion of women having to be close to home for taking care of the family seems to still persist’. (Bensch et al. 2016, 49)	Etienne and Robert (2024); Levine et al. (2022); UNICEF (2022); Sundararajan et al. (2021); Chen et al. (2021); Cundale et al. (2017); Bensch et al. (2016); Urmee and Harries (2011); Bensch et al. (2015)

**Table 10:** Examples of facilitators identified.

Theme and programmes reporting this finding	Evidence example	Papers contributing to this finding
Appropriate Technology	‘Almost all village representatives considered the Yeelen Ba technology as appropriate for their village. It was often highlighted that solar panels are the obvious second‐best choice, since grid electricity is unlikely to arrive soon in this remote area’. (Bensch et al. 2015, 66)	Cundale et al. (2017); Bensch et al. (2015)
Income Generation	‘The micro‐credit mechanism is recognized as the fundamental strength of the Bangladeshi SHS program and what has made it unique. It is seen to have allowed the SHS program to move away from a pure reliance on cash sales and to open up the market to a much greater number of households and small rural businesses that are unable to participate in a cash sales SHS program’. (Urmee and Harries 2011, 2827)	Wang et al. (2023); UNICEF (2022); Chen et al. (2021); Eras‐Almeida et al. (2019); Urmee and Harries (2011)
Financing	‘Personal perceived cost‐benefit reflects the economic characteristics of the project (such as income, subsidies, costs, and risks, etc.) that rural residents generally pay attention to. At the same time, some of them will adopt the project out of curiosity and personal image improvement (i.e., show off) brought by the project. I can collect my investment within 5 years and make profit in the next 15 years. (SX‐3.3‐4)’. (Wang et al. 2023, 6)	Wang et al. (2023); Eras‐Almeida et al. (2019)
Political Leadership	‘I trust the government and believe Solar PV poverty alleviation policy is a useful public assistance program … Since our country is vigorously promoting the solar PV poverty alleviation program, I think it is right to follow this national policy. National policies are always aligned with the people's work’. (Chen et al. 2021, 6–7)	Wang et al. (2023); UNICEF (2022); Chen et al. (2021); Eras‐Almeida et al. (2019)
Capacity Building	‘Training for program participants and for technicians installing and maintaining systems was considered by almost all program implementers to be costly but important. Training is considered a business strategy aimed at reducing the PO's costs of maintaining the systems’. (Urmee and Harries 2011, 2827)	Bahaj et al. (2019); Eras‐Almeida et al. (2019); Urmee and Harries (2011)
Programmatic Flexibility	‘It was initially planned to use a monthly post‐payment system to reduce costs, as it was hoped that the combination of a local manager and community peer pressure would enforce bill payment. However, this proved not to be the case and some businesses were unwilling or unable to pay their bills. Changing from monthly to bi‐weekly payments reduced the problem, but did not eradicate it. Therefore, at the beginning of 2014, a pre‐payment metering system was retrofitted to the mini‐grid, with all transactions taking place in the cooperative office, with electricity credit loaded onto the customers' smart card after payment This solved the non/late‐payment problem and within two months, 97% of bills were paid up‐to‐date’. (Bahaj et al. 2019, 13)	Levine et al. (2022); Eras‐Almeida et al. (2019): Bahaj et al. (2019)
Marketing and Awareness	‘The agents' repetition of key details persuaded villagers to participate in the project. Village leaders stressed the low operation cost and cheaper electricity, as well as the economic benefits and limited availability of government assistance, creating a sense of urgency’. (Chen et al. 2021, 6)	Wang et al. (2023); UNICEF (2022); Chen et al. (2021); Bensch et al. (2015); Urmee and Harries (2011)
Stakeholder Engagement	‘A well‐articulated partnership among promoters, the public sector, funders, and communities has helped to establish a lasting commitment to implement long‐term projects. Most importantly, to raise trust among partners, especially with the public sector and communities, it seems to be a plus for rural electrification that promoters have to be local companies … Acciona.org Peru and acciona.org Mexico recruit local people to lead and manage their projects, keeping a close relationship with acciona.org Spain’. (Eras‐Almeida et al. 2019, 10–11)	Levine et al. (2022); UNICEF (2022); Lo (2021); Eras‐Almeida et al. (2019)
Community Ownership	‘For example, in Peru, a committee consists of a group of at least three people, one of which has to be a woman. The committee works as a connection between the operator (acciona.org) and the beneficiaries, collecting fees and helping the operator on preventive maintenance. Rural electrification is promoting gender equality in Peru and Mexico, an initiative that ENERGETICA should embrace. One of the key aspects leading to the success of these projects is trust among partners, especially among local governments, promoters, and communities’. (Eras‐Almeida et al. 2019, 11)	Bahaj et al. (2019); Eras‐Almeida et al. (2019); UNICEF (2022) UNICEF (2022); Bahaj et al. (2019); Eras‐Almeida et al. (2019)
Technical Standardisation	‘The systems are covered under warranty during the microcredit loan period (three–four years). Providing system maintenance support and ensuring that spare components are readily available was seen by program implementers as important for increasing participant satisfaction, ensuring that the benefits of owning a SHS are maximised and for improving the overall sustainability of the program. Poor system performance was seen as a business risk as it would quickly translate into a loss of faith in the solar systems and to lead to reputation damage. The POs therefore tend to hold spare parts in offices within no more than a few kilometers from the project area’. (Urmee and Harries 2011, 2827)	Urmee and Harries (2011)
Other Facilitators	‘Another reason she gave for the higher take‐up observed in certain areas of the Kourinion and Djigouera district is the concentration of households of the Toussian ethnicity. Toussian women are known to have more influence on the decision making in their households. First, they may stress more the benefits of electricity for women and children and, second may persuade their husbands to rather rent a long‐lasting higher‐quality panel than to buy a lower‐quality private panel without any after‐sales service’. (Bensch et al. 2015, 34)	Ponticiello et al. (2023); Wang et al. (2023); Chen et al. (2021); Bahaj et al. (2019); Bensch et al. (2015); Urmee and Harries (2011)

###### 5.3.2.1.11 Other Barriers

Aside from the 10 pre‐identified themes, additional barriers and facilitators were also captured when presented in high, moderate or low quality qualitative and mixed‐methods studies. In total, nine papers provided additional barriers and six identified additional facilitators.

###### 5.3.2.1.12 Contextual Aspects

Existing gendered social norms and myths were reported to hamper desired intervention effects. In the EnDev Kenya programme, mobility constraints hindered women in the solar business, as expansion often required travel and overnight stays, which many husbands opposed (Bensch et al. 2016). This belief that women should stay home limited their business opportunities, leading to lower sales and income generation compared to their male counterparts. In Sierra Leone's rural renewable energy project, Levine et al. (2022) noted that it was initially believed that after the areas received electrification through the intervention, community members would seek treatment at community health centres (CHCs). However, some community members still preferred traditional healers, highlighting the need for community sensitisation to encourage the use of CHCs.

Natural disasters and health crises can also contribute to system losses and customer defaults, resulting in substantial disruption to interventions. In Bangladesh's SHS programme, the Sidor tornado in 2007 destroyed many SHS systems and panels (Urmee and Harries 2011). In China's PVPA programme, the COVID‐19 pandemic severely disrupted business activities, delayed installations and maintenance and reduced solar power generation efficiency (Chen et al. 2021).

Unfavourable economic environments were also reported to negatively affect interventions. Bensch et al. (2016) observed that local markets for solar and stoves in the EnDev Kenya programme quickly became saturated. Over half of the entrepreneurs in these sectors often had to sell outside their sub‐county, with many mentioning customer distance as a business challenge. In Bangladesh's SHS programme, Urmee and Harries (2011) identified rising fuel prices and increased transport costs as significant risks. These higher costs reduced the frequency of deliveries, causing delays in the supply of equipment and spare components.

###### 5.3.2.1.13 Implementation Side

Providing unreliable and low‐quality products was identified as a barrier in three papers. In the ERSEN programme in Senegal, flawed batteries deviated from the intended quality and negatively impacted the project's outcomes (Etienne and Robert 2024). In a different context, Bensch et al. (2015) found that subscription to the Yeelen Ba programme in Burkina Faso was hindered by insufficient power of the provided panels, as the panels could not adequately recharge essential devices. Similarly, UNICEF (2022) highlighted that many participants in the Mwangaza Mashinani programme in Kenya received non‐functional devices.

The lack of access to information on devices and payment options was another critical issue identified in two studies. Bensch et al. (2015) found that many Yeelen Ba users in the Sidi village of Burkina Faso preferred annual payments over monthly ones, but were unaware of the existing cheaper annual option. UNICEF (2022) observed that low literacy and numeracy levels among users hindered their ability to make informed decisions about devices. Additionally, their midline survey results showed that most households did not use the appropriate channels for reporting complaints, which diminished the grievance mechanism's effectiveness and reduced the likelihood of households receiving necessary support.

Conflicts of interest in hiring technicians were reported as a barrier in one programme. In Senegal, Etienne and Robert (2024) noted that a village chief appointed a family member to the paid role of system technician. This individual was criticised for poor knowledge of the system and improper management of the power station. Consequently, villagers decided to replace him with a more qualified local technician.

Lastly, one study highlighted concerns over battery theft in Rwanda, with some villagers claiming that their batteries were stolen by thieves or neighbours, causing anxiety among participants about securing their batteries (Sundararajan et al. 2021).

###### 5.3.2.1.14 Other Facilitators

####### Social and Economic Status

In a household solar lighting intervention in Uganda, adopting the technology enhanced participants' social status and respectability within their communities, reducing energy expenditure conflicts and encouraging household interactions (Ponticiello et al. 2023). In the Yeelan Ba programme in Burkina Faso, the social status of women within the Toussian ethnic group played a critical role as they were able to influence decision‐making and emphasise the benefits of electricity within their households, persuading their husbands to adopt sustainable energy solutions (Bensch et al. 2015).

####### Peer Influence and Community Relations

In China's PVPA programme, peer effects had a positive influence on the adoption of PV technology, where their willingness to adopt it increased through observing neighbours and engaging in community demonstrations (Chen et al. 2021; Wang et al. 2023). Similarly, participants of the Yeelan Ba programme in Burkina Faso reported a ‘leadership effect’, in which observing the experience of the first participant in their village was a strong factor for technology adoption (Bensch et al. 2015).

####### Unintended Consequences

Alongside barriers and facilitators to intervention effectiveness, five studies highlighted both positive and negative unintended consequences of programmes. In Senegal, some microgrid participants from the ERSEN programme believed that the presence of the microgrids meant their communities were overlooked for connections to the centralised grid, given that nearby villages had recently been connected (Etienne and Robert 2024). In the CAPS project in Malawi, some participants had taken to re‐wiring the solar aspect of their cookstove, repurposing it to power radios, lights and other technologies (Cundale et al. 2017). Three papers reported on the prestige that solar lights and microgrids had brought to households and communities. In Uganda, solar lights were viewed as a status symbol due to their visibility, with neighbouring children studying in households where lights had been supplied and participants recognising that they gained a sense of respect from others in the community (Ponticiello et al. 2023; Sundararajan et al. 2021). In China, the PVPA programme had led to a sense of pride in the community among participants. The funds received from the solar PVs perpetuated this sense of pride as they were used to improve roads, install public restrooms and generally improve living quality (Chen et al. 2021).

### 5.4 Integration of Quantitative and Qualitative Results

Our systematic review has addressed three research questions, two of which have utilised quantitative analysis based on impact evaluations, and one of which utilised qualitative analysis drawing from qualitative and mixed‐methods studies. Though these studies seek to answer different questions, there are opportunities to integrate the findings when they address the same cross‐cutting themes. In this section, we aim to use our qualitative analysis to help understand our quantitative findings. Readers are cautioned against drawing strong policy conclusions based on the discussion below, given the small number of studies on which these findings are based.

#### 5.4.1 Implementation Features

Our quantitative analysis showed few differences in effects across outcomes between programmes implemented at the local, regional and national levels. The exception to this was for time spent on leisure activities, where regional programmes were shown to have a larger effect than national programmes. Our qualitative analysis also supported the idea that regional programmes may lead to increased participant engagement under certain circumstances. Qualitative studies highlighted that regional and local programmes, those with greater levels of community participation and channels for community input, might lead to greater engagement amongst participants.

Quantitative analyses also found that for two outcomes, kerosene consumption and time spent resting, programmes which had government agencies as implementing partners were more effective than programmes with other implementing partners. Qualitative findings suggest there was greater engagement by participants when they saw their government promoting the programme. On the other hand, national programmes, often promoted by governments, may have issues when mechanisms for community participation are not present or when the level of trust between communities and governments is low.

#### 5.4.2 Programme Characteristics

Both the quantitative and qualitative analyses highlighted different features related to programme technologies and mechanisms that may help explain programme effectiveness. Across three outcomes, energy expenditure, time spent working and time spent studying, quantitative analyses found solar lamps/lanterns with mobile phone chargers to have larger positive effects than other, larger technologies. This is in contrast to the findings from qualitative studies, which highlighted that in some programmes, participants felt that the technologies provided to them did not meet their energy needs and were not of a large enough size. In these cases, participants purchased additional solar technologies. One qualitative study also highlighted the need to ensure that technologies are appropriate to local contexts; for instance, a programme brought a mobile phone charger from a neighbouring country where the programme was already operating, but it did not fit the mobile phones of the participants.

In terms of programme mechanism, the quantitative analysis found that providing information as well as financial support was more effective at reducing kerosene consumption compared to providing financial support on its own. This finding is supported by the qualitative analysis, where multiple studies highlighted the benefits of information and marketing components. Participants expressed positivity towards programmes where focal points were working in the community, who spread awareness of the technologies and the benefits of their uptake. Participants were also more prone to maintaining systems to a higher standard when programmes included an information mechanism dedicated to system maintenance.

#### 5.4.3 Programme Targeting

When assessing whether there were any differences in effects between programmes that targeted different actors (e.g., households, individuals, businesses) or populations (e.g., women, children), quantitative analyses found no significant differences across outcomes. Qualitative studies found that targeting different actors, women in particular, may act as either a barrier or facilitator to programme effectiveness, depending on the context. For example, while in one programme women were unable to travel for entrepreneurial purposes due to social norms, limiting their ability to engage with the programme, in a different context, women had significant influence over household purchase decisions and so could persuade their husbands to participate in the programme.

## 6 Discussion

### 6.1 Summary of Main Results

Our systematic review analysed the impact of off‐grid technology interventions on energy access, climate and socio‐economic outcomes in LMICs. We included 47 studies and identified a *relatively recent evidence base primarily focused on SSA*.

To assess the effectiveness of off‐grid interventions, we meta‐analysed the effects reported by included studies for each outcome in which there was sufficient and comparable evidence. We also conducted moderator analysis where possible, but due to the small sample sizes in each analysis, we urge caution when interpreting the results. In terms of *energy access outcomes*, we found that programmes significantly reduced kerosene consumption: there is a 66% chance that a recipient of off‐grid interventions will consume less kerosene than a person among those not receiving the intervention. We found a positive and significant increase in the usage of the off‐grid technologies being evaluated, such that there is a 67% chance that an intervention participant will be more likely to use the technology than someone not receiving the intervention. Similarly, the effect on the purchase and adoption of technologies may increase if the alternative is business as usual and when it is offered at a lower price: there is a 55% chance that a recipient will be more likely to adopt the technology than a person among those not receiving the intervention, and a 64% chance compared to a person among those receiving the technology at a lower price or with a larger subsidy. We also found a small increase in energy access such that the chance that a participant will have more access to energy than a person not receiving the intervention is 56%; however, we found no effect on other energy security measures, such as reliability and affordability; however, these findings draw from a very small sample of studies. Finally, there was no effect of these interventions on the hours of lighting usage or energy expenditure.

*Only five studies reported climate‐related outcomes*. We meta‐analysed the effects on air quality and found no significant change in indicators of air pollution. Only one study reported the effect of off‐grid interventions on CO_2_ emissions and therefore we could not provide any conclusions for this outcome category.

*The largest body of evidence was found for socio‐economic outcomes*. We identified a positive and significant, albeit very small, effect on income, such that the chance that an off‐grid intervention recipient will have a larger income than a person from the control group is 52%. Off‐grid technologies do not change time allocation other than the time spent studying: there is a 53% chance that a participant will spend more time studying than a person among those not receiving the intervention. For children, this chance is 54%; however, we found no effects on school attendance or test scores. This finding, however, may overstate the true effect, as the evidence shows signs of publication bias.

We also did not find a significant change in respiratory illnesses and symptoms associated with off‐grid energy programmes. Finally, based on a very small sample of studies, we found a small and significant increase in women's empowerment, measured by decision‐making indicators: there is a 53% chance that a female participant in off‐grid interventions will be more empowered to make decisions than a woman among those not receiving the intervention. Due to the heterogeneity of the studies included in our review, as well as a lack of data, we were unable to complete moderator analyses for most of the variables considered.

We also sought to understand barriers and facilitators to programme success. Drawing on Sovacool's (2013) framework, we identified finance, local involvement, information and technical knowledge as factors that may lead to programmes being more or less effective.

*Financial support is an important mechanism by which programmes may aim to increase the uptake and use of off‐grid technologies.* However, findings from the qualitative analysis highlighted that in some circumstances, subsidies provided to households were not large enough, and potential participants were still unable to afford technologies. Qualitative studies also underlined that including an income‐generating aspect within programmes, such as microcredit, may allow businesses and suppliers of off‐grid technologies to take greater risks as they may not be as dependent on sales to expand their operations.

*Local involvement in the development and implementation of programmes was found to be a factor in the success of programmes, especially for those implemented at the national level.* Studies stressed that programmes implemented at the national level may not have adequate mechanisms in place for input from local stakeholders. When programmes did involve local stakeholders and community leaders, participants appreciated this as they felt that their voices were being heard. However, in some circumstances, even when there were community meetings and pathways for involvement, local stakeholders still felt that their voices were not listened to.

*Information and marketing were highlighted in multiple qualitative studies as a key factor in participant engagement*. Having programme operators available for discussions and marketing within communities contributed to greater visibility of new technologies and programmes. However, reaching out to scattered and rural populations can also pose challenges.

*Providing technical knowledge was also emphasised by qualitative studies as a way to ensure that systems were maintained, though there were barriers to delivering this knowledge.* Supporting households and individuals in their ability to maintain the systems provided is one way in which programmes could have sustained effects. However, low literacy and numeracy levels can be an issue for knowledge transfer in some cases. In others, trained engineers for maintaining community systems, such as microgrids, can leave the programme for higher‐paid roles elsewhere.

### 6.2 Overall Completeness and Applicability of Evidence

Our review has taken a broad approach to the topic of off‐grid technologies with no exclusion criteria placed on the mechanism of included programmes, which allowed for the inclusion of many different interventions and technologies to address the research questions. Despite this, the evidence base we identified is uneven across contexts, mechanisms and technologies.

Twenty‐nine of the impact evaluations reported energy access outcomes. These studies mainly focused on SSA (mostly East Africa) and South Asia (mostly India), and the interventions were primarily based on financial mechanisms. This makes it difficult to generalise results around energy access to other regions and mechanisms. In contrast, we identified very few studies, only five, that evaluated climate outcomes. All five of these studies were based in SSA, and four were in countries in Eastern Africa. These studies mainly focused on solar lamps/lanterns and cookstoves, using financial or direct provision mechanisms. The small number of studies evaluating climate outcomes likely represents a small sample of off‐grid interventions in LMICs. Lastly, 32 studies reported socio‐economic outcomes. While these studies evaluated a range of technologies, they mostly used the direct provision mechanism in SSA countries. Hence, the findings related to socio‐economic outcomes may have reduced external validity to other regions and mechanisms.

### 6.3 Quality of the Evidence

The risk of bias assessment aims to identify potential biases introduced at the design, implementation, analysis or reporting of impact evaluations. The majority of the included impact evaluations were assessed as having a high risk of bias, and only six RCTs and one QED were rated as having a low risk of bias. Despite the overall risk of bias scores, most studies were assessed as having a high risk of bias due to failing to meet only one bias dimension; fewer studies had extensive bias issues across multiple dimensions. In contrast, almost two‐thirds of the qualitative studies appraised were rated as high or moderate quality. However, we excluded 20% of our qualitative studies due to critical flaws in conducting and/or reporting the research.

Aside from the quality of the primary evidence, studies included in the review often measured similar outcome constructs but with a broad range of indicators. This hindered the direct comparison of effects across studies. For instance, while ‘access to energy’ should, intuitively, be a straightforward outcome, the three studies evaluating this outcome reported three different measures (i.e., whether a household has electricity, whether a household has access to a grid connection, and whether a household has access to Tier 1 energy for lighting). Heterogeneity across outcomes also extended to composite measures, such as energy expenditure and time allocation. For instance, time spent on leisure activities was measured using a combination of different indicators (e.g., watching TV, listening to the radio, reading, playing), while some studies did not specify individual activities covered by leisure time.

### 6.4 Potential Biases in the Review Process

Our review process has several limitations that should be considered when interpreting our results. First, limited data meant we were unable to fully evaluate outcomes as presented in our conceptual framework (Figure 1). Though our conceptual framework presents socio‐economic improvements occurring as a result of increases in energy access and positive climate outcomes, the limited number of studies that assessed each outcome meant that we were only able to test for direct effects between interventions and outcome groups.

Second, the prioritisation of outcomes and the heterogeneity with which they were reported limited the comparability of outcomes. The data extraction process prioritised aggregated outcome measures (e.g., energy expenditure), and individual measures (e.g., expenditure on lighting, phone charging, kerosene, batteries, etc.) were only extracted when no composite measures were reported. In these cases, we then constructed composite measures based on these individual measures. This meant that we were unable to identify and explore the full list of studies that reported relevant but specific outcome measures.

Third, missing data in the reports meant that some effects or studies were excluded from the meta‐analysis as there was insufficient data to calculate effect sizes. Though we made every effort to contact authors to obtain additional data, when authors did not respond, we had to exclude the effects or studies.

### 6.5 Agreements and Disagreements With Other Studies or Reviews

This systematic review is the most up‐to‐date and comprehensive review on the effects of off‐grid technology interventions in LMICs. Our findings have agreements and disagreements with previous reviews, which can mainly be explained by the comprehensiveness of our review.

The review from Huang et al. (2022) was based on a descriptive synthesis of 44 studies solely focusing on the PVPA programme in China. However, both reviews found an increase in the income of off‐grid technology programme participants. Cissé's (2022) review looked at sustainable development outcomes, but its main objective was to assess whether experimental or QEDs were more likely to identify favourable outcomes. The review found similar results across designs. Our moderator analysis also found a few differences between study designs, though we identified significant differences for two outcomes, energy expenditure and time spent resting. Our review cannot be directly compared to that of Khogali et al. (2022), which found positive impacts of electrification on health system indicators, such as quality of service and mortality. The health outcome we were able to analyse (respiratory illness, for which we found no significant effect) was not reported in the previous review.

Finally, our findings are in both agreement and disagreement with those of Moore et al. (2020). In terms of energy access, Moore et al. found a non‐statistically significant effect of electrification interventions (both on and off‐grid) on lighting use and technology ownership. Our review identified a small but significant positive effect on technology uptake or upgrade, but a non‐statistically significant effect on lighting use. For education outcomes, both reviews found a significant and positive effect on study time as well as a positive but non‐significant effect on test scores. In addition, but both reviews also found non‐significant results on time spent on domestic activities, active hours (time spent resting) and employment. Differences between the reviews may be explained by Moore's inclusion of on‐grid electrification programmes, an intervention excluded from our review.

## 7 Authors' Conclusions

### 7.1 Implications for Practice and Policy

Based on the findings of this review, we suggest that future programming consider the following areas to help ensure that the provision of technologies is also building on/drawing from structures that enable their success:

- Ensure that individual off‐grid programmes draw from or are *aligned with national policies and frameworks* to facilitate buy‐in and support programme sustainability.
- *Involve national and local governments as implementation partners*, particularly when levels of trust between communities and governments is high. Seeing government agencies actively involved in programmes can contribute to greater engagement and, thus, to improved outcomes at the household level.
- Embed *appealing income‐generating activities* into off‐grid programmes to attract implementers and support programme sustainability.
- *While promoting solar lamps/lanterns with mobile phone chargers can be a safe bet, confirming the energy needs of communities and households is a prerequisite.* The design of future programmes should consider building in scoping exercises to identify the energy needs and current infrastructure of households and communities. Ensure that there are also sufficient resources for system/equipment maintenance and support services to enhance the sustainability of programmes and avoid negative participant experiences.
- Identify *subsidies, payment structures, and other suitable ways to make access to off‐grid technologies attainable*, both for initial adoption as well as for the continued operation of the off‐grid technologies.
- Consider offering *incentives and capacity building* into programme designs to help attract and retain high‐quality staff.
- *Ensure that the provision of key information is built into off‐grid programmes, including stating the benefits of using the technologies, as well as how to use and maintain them.* Integrating information components within programmes can increase the levels of engagement and technology maintenance.
- Design programmes with *clear communication systems across the delivery chain and channels for community input* to increase engagement and ownership, particularly in regional and local programmes. When technology‐based communication systems are being considered, verify that the technology literacy of participants is sufficient to engage effectively.
- Consider *gender transformative approaches* to help overcome gendered norms and mobility constraints that may limit the participation of women.

### 7.2 Implications for Research

To keep building up the evidence base around off‐grid technologies, the findings of this review point to several action points for future research, including:

- *Researchers can contribute to building up this evidence base by focusing on contextual gaps*. For instance, the evidence base in regions beyond SSA (outside East Africa in particular) and South Asia should be increased, and more supply‐side off‐grid interventions or those with information provision components should be evaluated. Additionally, few studies conducted analyses focusing on the gendered impacts of off‐grid interventions.
- *Researchers should design studies that include standard outcome measures.* While there are agreed key concepts across SDG7, such as *energy security*, researchers and research commissioners can advocate for the use of standard indicators to increase comparability across programmes. This may also help build up evidence of the effects of these interventions on energy access and climate‐related outcomes.
- *Future research should mitigate the risks of common biases and flaws.* Developing approaches to anticipate and mitigate biases and critical flaws in research can help improve the quality of the off‐grid evidence base, which can lead to growing confidence and greater opportunities to make stronger policy recommendations.
- *Future research should prioritise mixed‐methods evaluations to understand why off‐grid interventions may or may not be effective.* Researchers and commissioners can consider embedding qualitative components into impact evaluations to leverage the information from programme implementation and allow for a richer understanding of programmes from its participants' perspective.
- *Future studies should aim for more transparent and comprehensive reporting.* In our review, we found signs of publication bias for just one outcome—time spent studying—but this suggests that some effects may appear stronger than they really are if studies with smaller or null results are not published. To improve the reliability of future evidence, researchers should consider registering their studies in advance and sharing both significant and non‐significant results. Funders and journals can also help by encouraging the publication of all findings, not just those with positive results.

## Author Contributions

Content: Cem Yavuz, Constanza Gonzalez Parrao, Zafeer Ravat, María Daniela Anda León, Sanghwa Lee, Paulo Fernandes, Quinn Reifmesser, Frederick Gaved, Samantha Pilato. Systematic review methods: Constanza Gonzalez Parrao, Cem Yavuz, Zafeer Ravat, María Daniela Anda León, Birte Snilstveit. Statistical analysis: Constanza Gonzalez Parrao, María Daniela Anda León, Zafeer Ravat, Cem Yavuz. Information retrieval: Cem Yavuz, Paulo Fernandes.

## Conflicts of Interest

The authors declare no conflicts of interest.

## 1 Transparent Peer Review

The peer review history for this article is available at: https://www.webofscience.com/api/gateway/wos/peer-review/10.1002/cl2.70060.

## Plans for Updating This Review

There are currently no plans to update this review.

## Differences Between Protocol and Review

The citation for our qualitative critical appraisal tool has been updated since the publication of our protocol to more accurately reflect its origins.

## Sources of Support

This review was co‐funded by Sustainable Energy for All (SEforALL) and 3ie.

## Supporting information

REVISED ‐ Appendices ‐ Off‐Grid Technology SR.

supmat.docx.

## Data Availability

Full search strings alongside full data set used for the quantitative synthesis, along with its corresponding codebook, can be accessed in 3ie's Dataverse (forthcoming). https://dataverse.harvard.edu/dataverse/3ie.

## Endnotes

1 Available at: https://www.3ieimpact.org/blogs/towards-sdg-7-mapping-evidence-access-sustainable-energy-lmics.

2 We considered two or more papers linked when they were published by the same authors studying the same intervention and research question. In many cases, linked papers have the same sample and analysis but a different publication status (e.g., working paper and journal article). In other cases, papers use a subset of a data set of the main paper(s), and/or include additional analysis, and/or measures additional outcomes.

3 Fourteen studies evaluated either a solar lamp/lantern with or without a mobile phone charger. This is fewer than the sum of these technologies in Table 5 as multiple studies had separate treatment arms for each technology.

4 Lucchino (2017) reported a series of studies, with two chapters conducting separate RCTs to measure different programmes that we included in our synthesis. In this case, each chapter was assessed separately. Araujo Bonjean et al. (2015) used a combination of an RCT, difference‐in‐difference, and propensity score matching techniques in their analysis, requiring separate risk of bias assessments for each RCT and QED method.

5 We were unable to include Banerjee et al. (2011) in this analysis due to incomplete sample size data. We contacted authors but they were unable to provide the additional data required for analysis.

6 We were unable to include Banerjee et al. (2011) in this analysis due to incomplete sample size data. We contacted authors but they were unable to provide the additional data required for analysis.

7 We were unable to include Banerjee et al. (2011) in this analysis due to incomplete sample size data. We contacted authors but they were unable to provide the additional data required for analysis.

8 We were unable to include Banerjee et al. (2011) in this analysis due to incomplete sample size data. We contacted authors but they were unable to provide the additional data required for analysis.
