# Supplementary material for: Improving Energy Access, Climate and Socio‐Economic Outcomes Through Off‐Grid Electrification Technologies: A Systematic Review
Source: Campbell Syst Rev. 2025 Aug 15;21(3):e70060. doi: 10.1002/cl2.70060 (PMC12355195; doi:10.1002/cl2.70060)
Supplement: Supplementary file 1 — REVISED ‐ Appendices ‐ Off‐Grid Technology SR. [file CL2-21-e70060-s002.docx]

**Improving energy access, climate and socio-economic outcomes through off-grid electrification technologies: A systematic review**

**Appendices**

**Contents**

[Appendix A: Inclusion criteria 1](#_Toc184130972)

[A.1: Study designs 1](#_Toc184130973)

[A.2: Participants 3](#_Toc184130974)

[Appendix B: Evidence Gap Map to Systematic Review process 1](#_Toc184130975)

[B.1: Intervention selection process 1](#_Toc184130976)

[B.2: Outcome selection process 1](#_Toc184130977)

[B.3: Outcome prioritisation 2](#_Toc184130978)

[Appendix C: Example search strategy 6](#_Toc184130979)

[Database Example 6](#_Toc184130980)

[Grey Literature Example: 10](#_Toc184130981)

[Appendix D: Data extraction forms 11](#_Toc184130982)

[D.1: Study characteristics form 11](#_Toc184130983)

[D.2: Quantitative extraction form 14](#_Toc184130984)

[Appendix E: Risk of bias tools 19](#_Toc184130985)

[E.1: Quantitative risk of bias tool 19](#_Toc184130986)

[E.2: Qualitative risk of bias tool 28](#_Toc184130987)

[Appendix F: Characteristics of included studies 37](#_Toc184130988)

[F.1: Overview of included studies 37](#_Toc184130989)

[F.2: Moderator characteristics of included studies 43](#_Toc184130990)

[Appendix G: Examples of excluded studies 49](#_Toc184130991)

[Appendix H: Risk of bias and critical appraisal results 51](#_Toc184130992)

[H.1: Risk of bias assessment of experimental impact evaluations 51](#_Toc184130993)

[H.2: Risk of bias assessment of quasi-experimental impact evaluations 55](#_Toc184130994)

[H.3: Critical appraisal of qualitative studies 57](#_Toc184130995)

[H.4: Critical appraisal of mixed-methods studies 58](#_Toc184130996)

[Appendix I: Funnel plots 60](#_Toc184130997)

[Appendix J: Moderator analyses (full results) 69](#_Toc184130998)

[Appendix reference list 75](#_Toc184130999)

**List of Tables**

[Appendix Table 1. Income classifications of countries 3](#_Toc183160496)

[Appendix Table 2. Table of SR outcomes mapped to EGM outcomes 4](#_Toc183160497)

[Appendix Table 3. Full moderator results 69](#_Toc183160498)

**List of Figures**

[Appendix Figure 1. Funnel plot for hours of lighting use 60](#_Toc183078924)

[Appendix Figure 2. Funnel plot for energy expenditure (all) 60](#_Toc183078925)

[Appendix Figure 3. Funnel plot for household (or personal) income 61](#_Toc183078926)

[Appendix Figure 4. Funnel plot for time spent working (renumerated) 62](#_Toc183078927)

[Appendix Figure 5. Funnel plot for time spent on domestic work 63](#_Toc183078928)

[Appendix Figure 6. Funnel plot for time spent on leisure (hours) 64](#_Toc183078929)

[Appendix Figure 7. Funnel plot for time spent study (all) 65](#_Toc183078930)

[Appendix Figure 8. Funnel plot for time spent studying (children only) 66](#_Toc183078931)

[Appendix Figure 9. Funnel plot for respiratory illness 67](#_Toc183078932)

# Appendix A: Inclusion criteria

## A.1: Study designs

We included studies that look at the effect of an intervention aimed at promoting sustainable energy in L&MICs. Specifically, we included studies that adopt methods estimating effects that can be attributed to an intervention, as compared to what would have happened in the absence of the intervention.

Studies were excluded if they did not evaluate the effectiveness of an intervention delivered in a real-world setting (i.e., experiments conducted in tightly controlled settings, like those of a laboratory) were excluded. Screening questions used to help determine whether a study qualified as an effectiveness study included (answering yes signals the study may have been conducted in a lab setting):

- Is the study primarily designed to determine to what extent a specific technique, technology, treatment, procedure or service works under ideal conditions rather than attempt to answer a question relevant to the roll- out of a large programme?
- Is the intervention being carried out by the researchers themselves (e.g. by applying fertiliser in test plots to measure effects on plant growth), or by the people who would carry it out at scale (e.g. farmers applying fertilizer to their crops)?
- Does the study evaluate an intervention that is, or could easily be implemented as, a social policy or programme, or is it “basic science” research on biophysical mechanisms?
- Are participants exposed to an artificial scenario created by the researchers to simulate a policy or programme, rather than being exposed to such a programme in the real world (i.e., is the study an artefactual/framed field experiment)?

We excluded studies that test the efficacy of off-grid technologies. Often utilizing quantitative energy models (Sovacool et. al. 2018), efficacy studies test the effects of technologies under simulated environments, abstract from real-world settings.

Particularly, we included studies that implement at least one of the following study designs that are widely used to evaluate intervention effectiveness (Aloe et. al. 2017; Reeves et. al. 2017).

A) Prospective studies that allocate participants to treatment and control groups using random assignment or quasi-experimental methods:

1. Randomized controlled trials (RCTs), with assignment at individual, household, community, or other cluster level, and quasi-RCTs using prospective methods of assignment (such as alternation).
2. Natural experiments with clearly defined intervention and comparison groups, which exploit natural randomness in implementation assignment by decision makers (e.g. public lottery) or random errors in implementation.

B) Non-randomized designs with selection on unobservables:

1. Regression discontinuity designs (RDD) or fuzzy-RDD
2. Instrumental variables (IV)
3. Endogenous treatment-effects models, endogenous switching regression, and other methods synonymous to the Heckman two step model.
4. Difference-in-differences (DID), two-way fixed-effects (TWFE), and two-way Mundlak regressions (TWM):
5. DiD models include an interaction term between a time and intervention variable in a regression model. They may also regress an intervention variable on an outcome variable measuring the changes in outcomes over time or present a t-test comparing changes in outcomes over time between the intervention and control group.
6. TWFE regressions must include time fixed-effects and unit fixed-effects at the level of the intervention (or lower). For example, if the intervention varies at a village level, it must include either village fixed-effects or fixed-effects of a smaller unit, such as households.
7. Interrupted time series (ITS) models, with or without a contemporaneous comparison group. The ITS regression model must adjust for autocorrelation, or it can use autoregressive integrated moving-average (ARIMA) models. An ITS model should include pre-intervention outcome data for a minimum of three time periods.
8. Weighting and matching approaches which control for observable confounding, including non-parametric approaches (e.g. statistical matching, covariate matching, coarsened-exact matching, propensity score matching) and parametric approaches (e.g. propensity-weighted multiple regression analysis).
9. Synthetic control methods.

Natural experiments where the assignment to intervention and control groups was not part of a planned experiment could use different includable designs (e.g. RCT, RDD, ITS). These cases were categorised as RCT, RDD, ITS, etc.

## A.2: Participants

We included studies that evaluated interventions in low- and middle-income countries (L&MICs) as defined by the World Bank income group classification (Appendix Table 1).^[[1]](#footnote-2)^ Studies were classified based on the year the intervention began.^[[2]](#footnote-3)^ Multi-country studies were included if there was at least one effect estimate reported for an L&MIC. Within these countries, we did not exclude studies based on their setting.

Appendix Table 1. Income classifications of countries

| **LOW- AND MIDDLE-INCOME COUNTRIES (L&MICs)** | | | | | |
| --- | --- | --- | --- | --- | --- |
| Afghanistan | | Eritrea | Marshall Islands | | Syrian Arab Republic |
| Albania | | Ethiopia | Mauritania | | Tajikistan |
| Algeria | | Fiji | Mexico | | Tanzania |
| Angola | | Gabon | Micronesia, Federal States | | Thailand |
| Armenia | | Gambia, The | Moldova | | Timor-Leste |
| Azerbaijan | | Georgia | Mongolia | | Togo |
| Bangladesh | | Ghana | Montenegro | | Tonga |
| Belarus | | Grenada | Morocco | | Tunisia |
| Belize | | Guatemala | Mozambique | | Turkey |
| Benin | | Guinea | Myanmar | | Turkmenistan |
| Bhutan | | Guinea-Bissau | Namibia | | Tuvalu |
| Bolivia | | Guyana | Nauru | | Uganda |
| Bosnia and Herzegovina | | Haiti | Nepal | | Ukraine |
| Botswana | | Honduras | Nicaragua | | Uzbekistan |
| Brazil | | India | Niger | | Vanuatu |
| Bulgaria | | Indonesia | Nigeria | | Vietnam |
| Burkina Faso | | Iran, Islamic Republic | Pakistan | | West Bank and Gaza |
| Burundi | | Iraq | Papua New Guinea | | Yemen, Republic |
| Cambodia | | Jamaica | Paraguay | | Zambia |
| Cameroon | | Jordan | Peru | | Zimbabwe |
| Cape (Cabo) Verde | | Kazakhstan | Philippines | |  |
| Central African Republic | | Kenya | Rwanda | |  |
| Chad | | Kiribati | Samoa | |  |
| China | | Korea, Democratic Republic | São Tomé and Principe | |  |
| Colombia | | Kosovo | Senegal | |  |
| Comoros | | Kyrgyz, Republic | Serbia | |  |
| Congo, Democratic Republic | | Lao PDR | Sierra Leone | |  |
| Congo, Republic | | Lebanon | Solomon Islands | |  |
| Costa Rica | | Lesotho | Somalia | |  |
| Côte d'Ivoire (Ivory Coast) | | Liberia | South Africa | |  |
| Cuba | | Libya | South Sudan | |  |
| Djibouti | | Macedonia, FYR | Sri Lanka | |  |
| Dominica | | Madagascar | St. Lucia | |  |
| Dominican Republic | | Malawi | St. Vincent and the Grenadines | |  |
| Ecuador | | Malaysia | Sudan | |  |
| Egypt, Arab Republic | | Maldives | Suriname | |  |
| El Salvador | | Mali | Swaziland | |  |
| **FORMER LOW- AND MIDDLE-INCOME COUNTRIES** | | | | | |
| Czechoslovakia | | | | | |
| Gibraltar (High income: 2009-2010) | | | | | |
| Mayotte (High income: 1990) | | | | | |
| Netherlands Antilles (High income: 1994-2009) | | | | | |
| Serbia and Montenegro | | | | | |
| USSR | | | | | |
| Yugoslavia | | | | | |
| **TRANSITIONAL COUNTRIES** | | | | | |
| **Name** | **L&MIC period** | | | **High-income country period** | |
| American Samoa | 1990-present | | | 1987-1989 | |
| Antigua and Barbuda | 1987-2001; 2003-2004; 2009-2011 | | | 2002; 2005-2008; 2012-present | |
| Argentina | 1987-2013; 2015-2016; 2018-present | | | 2014; 2017 | |
| Aruba | 1991-1993 | | | 1987-1990; 1994-present | |
| Bahrain | 1990-2000 | | | 1987-1989; 2001-present | |
| Barbados | 1987-1988; 1990-1999; 2001; 2003-2005 | | | 1989; 2000; 2002; 2006-present | |
| Chile | 1987-2011 | | | 2012-present | |
| Croatia | 1992-2007; 2016 | | | 2008-2015; 2017-present | |
| Cyprus | 1987 | | | 1988-present | |
| Czech Republic | 1992-2005 | | | 2006-present | |
| Equatorial Guinea | 1987-2006; 2015-present | | | 2007-2014 | |
| Estonia | 1991-2005 | | | 2006-present | |
| Guam | 1990-1994 | | | 1987-1989; 1995-present | |
| Greece | 1987-1995 | | | 1996-present | |
| Hungary | 1987-2006; 2012-2013 | | | 2007-2011; 2014-present | |
| Isle of Man | 1990-2001 | | | 1987-1989; 2002-present | |
| Latvia | 1991-2008; 2010-2011 | | | 2009; 2012-present | |
| Lithuania | 1991-2011 | | | 2012-present | |
| Macao (SAR) | 1987-1993 | | | 1994-present | |
| Malta | 1987-1988; 1990-1997; 1999; 2001 | | | 1989; 1998; 2000; 2002-present | |
| Mauritius | 1987-2018; 2020-present | | | 2019 | |
| Nauru | 2016-2018 | | | 2015; 2019-present | |
| New Caledonia | 1987-1994 | | | 1995-present | |
| Northern Mariana Islands | 1992-1994; 2002-2006 | | | 1995-2001; 2007-present | |
| Oman | 1987-2006 | | | 2007-present | |
| Palau | 1987-2015; 2021-present | | | 2016-2020 | |
| Panama | 1987-2016; 2020 | | | 2017-2019; 2021-present | |
| Poland | 1987-2008 | | | 2009-present | |
| Portugal | 1987-1993 | | | 1994-present | |
| Puerto Rico | 1987-1988; 1990-2001; | | | 1989; 2002-present | |
| Republic of Korea (aka South Korea) | 1987-1994; 1998-2000 | | | 1995-1997; 2001-present | |
| Romania | 1987-2018; 2020 | | | 2019; 2021 | |
| Russia | 1991-2011; 2015-present | | | 2012-2014 | |
| Seychelles | 1987-2013 | | | 2014-present | |
| Slovak Republic | 1992-2006 | | | 2007-present | |
| Slovenia | 1992-1996 | | | 1997-present | |
| Saudi Arabia | 1990-2003 | | | 1987-1989; 2004-present | |
| St. Kitts and Nevis | 1987-2010 | | | 2011-present | |
| Trinidad and Tobago | 1987-2005 | | | 2006-present | |
| Uruguay | 1987-2011 | | | 2012-present | |
| Venezuela | 1987-2013; 2015-present | | | 2014 | |

# Appendix B: Evidence Gap Map to Systematic Review process

## B.1: Intervention selection process

Moving from the EGM into our SR was a process which involved multiple steps for both interventions and outcomes. Our original EGM was built around a framework encompassing SDG 7 and encapsulated energy access, efficiency and the use of renewable energy. After completing our EGM and presenting the results to SEforALL, they highlighted several areas of potential interest for this SR. Based on these areas, and having explored our EGM to see which topics were ripe for synthesis (intervention-outcomes areas with high numbers of impact evaluations and no recent medium or high confidence systematic reviews), we agreed upon the topic of off-grid technologies.

After having agreed to focus on off-grid technologies, one member of the core research team then filtered the EGM (using the EGM’s technology filter) to identify all those which were off-grid. The full list of technologies was then presented to SEforALL and three technologies were cut and excluded from our review, these were: solar dryers (Matavel et al., 2022; Nagwekar et al., 2020), solar-powered mosquito fans (Homan et al., 2016) and solar suitcases (Rokicki et al., 2019). Unlike the off-grid technologies included in our review, these technologies provide one specific use and so were expected to have narrow causal pathways which focused on few outcomes.

With the full list of studies based on these interventions, we then moved on to selecting the outcomes to be included in our review.

## B.2: Outcome selection process

As well as providing the scope for a review of off-grid technologies, SEforALL also proposed an outcome framework based on energy access, climate and socio-economic outcomes (broadly similar to our EGM outcome framework). We began by mapping our EGM outcomes into this new framework and confirming this mapping with SEforALL.

Once we had this confirmed, we then scoped which outcomes our studies (relevant based on their technology) evaluated (based on our EGM’s data). Based on this scoping, we were able to drop seven EGM outcomes from our review.

For the newly formed **energy access** category, the EGM outcomes *knowledge, awareness and attitudes* as well as *behaviour adoption (excluding technology usage)* were dropped for having little relevance to energy access (in the case of *behaviour adoption* no studies of relevance evaluated it as an outcome).

For the newly formed **climate** outcome group, the EGM outcome, *water consumption*, was dropped as no relevant studies evaluated this as an outcome.

Finally, for the newly formed **socio-economic** outcome group, the EGM outcomes, *assets value*, *land productivity*, *public service delivery* and *crime and security* were dropped as they were included in relatively few studies, compared to other EGM outcomes placed in the socio-economic group. Across all three groups, any EGM outcome that was measured in fewer than three relevant studies was dropped, ultimately, we were left with 12 EGM outcomes of relevance. The decision to drop these categories was also made in light of resource constraints.

At this stage we had agreed upon the EGM outcome that would be included in our review, and how these mapped to the new groups (energy access, climate and socio-economic) that we had created. A further stage of refinement was necessary, however, as many of our EGM categories were broad and included multiple outcomes within them. Our aim here was to take the larger, more heterogenous categories, and to form smaller homogenous outcomes.

For example, the EGM category *education*, included any education outcome or indicator. When looking at how our studies of relevance measured education, we identified three distinct measures. These were *study hours*, *school attendance* and *test scores*. When looking at all outcome categories from the EGM we saw similar trends. At this point, to increase the likelihood that we could conduct meta-analysis on certain outcomes we chose to expand some of the larger EGM categories into multiple categories for this review (Appendix Table 2).

When deciding whether or not to expand a category, based on our scoping, a clear cut-off of five studies emerged. This meant that if five or more studies measured an EGM outcome using a certain measure or indicator, then it would be expanded.

## B.3: Outcome prioritisation

Following previous 3ie systematic reviews, we utilised a process known as outcome prioritisation to ensure that the scope of the review remained manageable. Outcome prioritisation was the next step in deciding which outcomes would be extracted for our review. Once we had agreed on the included outcomes, we followed a similar process to look at what indicators were most common, for example, energy expenditure as an outcome was measured with different indicators across studies.

Across all outcomes, our priority was to extract composite measures. For instance, if a study reported energy expenditure as a composite measure including expenditure across all energy sources, this was extracted. We only extracted measures for single sources, such as expenditure on lighting or candles, when composite measures were not available. Using the single sources reported within studies, we were then able to create composite measures.

Given our priority of extracting composite measures, we only report on the analysis for composite measures. Any analysis of single measures would not be accurate given that some studies contained both composite and single measures, in which case only composite measures were extracted. Any analysis of single measures, such as expenditure on candles, would not include all possible studies.

Appendix Table 2. Table of SR outcomes mapped to EGM outcomes

| **Outcome Group** | **EGM Outcome category** | **SR Outcome** | **Preferred indicator** | **Secondary indicator** | **Tertiary indicator** |
| --- | --- | --- | --- | --- | --- |
| Energy Access | Energy net savings or consumption | **Lighting hours** | Lighting hours per day (overall) | Lighting hours per day (appliance specific) | NA |
|  |  | **Kerosene use** | Kerosene consumption | Kerosene expenditure | NA |
|  | Technology uptake | **Technology uptake** | Adoption or ownership | NA | NA |
|  | Usage of the technology | **Technology usage** | Recall of usage | Recharge frequency | NA |
|  | Energy security | **Energy security** | Any energy security indicator | NA | NA |
| Climate | Air quality/ pollution | **Air quality** | PM 2.5 | NA | NA |
|  | GHG emissions | **GHG emissions** | CO2 emissions | NA | NA |
| Socio-economic | Income, savings and expenditure | **Income** | Household or personal income (overall) | Other income sources | NA |
|  |  | **Energy expenditure** | Energy expenditure (aggregated) | Lighting expenditure (aggregated) | Disaggregated energy expenditure |
|  | Health status, comfort and wellbeing | **Respiratory illness** | Respiratory illness indices or composite measures | Disaggregated respiratory issues | NA |
|  |  | **Eye illness** | Eye problem indices or composite measures | Disaggregated eye issues | NA |
|  | Education | **Study hours** | Composite measure of study time | Home based study time | NA |
|  |  | **School attendance** | Time spent in school | Absenteeism | NA |
|  |  | **Test scores** | Aggregated test scores | Disaggregated subject test scores | NA |
|  | Allocation of time | **Active hours** | Rest time (including sleeping) | Hours awake | NA |
|  |  | **Household/ domestic activities** | Composite measure of time spent on household/ domestic activities | Time spent on disaggregated household/ domestic activities. | NA |
|  |  | **Leisure activities** | Composite measure of time spent on leisure activities | Time spent on disaggregated leisure activities. | NA |
|  | Employment | **Employment** | Working hours (overall) | Working hours (disaggregated) | NA |
|  | Women's empowerment | **Women's empowerment** | Decision making index | Disaggregated measure of decision making | NA |

# Appendix C: Example search strategy

Database Example

CAB Abstracts <1973 to 2023 Week 25>

Search Strategy:

1 (afghanistan or albania or algeria or american samoa or angola or "antigua and barbuda" or antigua or barbuda or argentina or armenia or armenian or aruba or azerbaijan or bahrain or bangladesh or barbados or republic of belarus or belarus or byelarus or belorussia or byelorussian or belize or british honduras or benin or dahomey or bhutan or bolivia or "bosnia and herzegovina" or bosnia or herzegovina or botswana or bechuanaland or brazil or brasil or bulgaria or burkina faso or burkina fasso or upper volta or burundi or urundi or cabo verde or cape verde or cambodia or kampuchea or khmer republic or cameroon or cameron or cameroun or central african republic or ubangi shari or chad or chile or china or colombia or comoros or comoro islands or iles comores or mayotte or democratic republic of the congo or democratic republic congo or congo or zaire or costa rica or "cote d’ivoire" or "cote d’ ivoire" or cote divoire or cote d ivoire or ivory coast or croatia or cuba or cyprus or czech republic or czechoslovakia or djibouti or french somaliland or dominica or dominican republic or ecuador or egypt or united arab republic or el salvador or equatorial guinea or spanish guinea or eritrea or estonia or eswatini or swaziland or ethiopia or fiji or gabon or gabonese republic or gambia or "georgia (republic)" or georgian or ghana or gold coast or gibraltar or greece or grenada or guam or guatemala or guinea or guinea bissau or guyana or british guiana or haiti or hispaniola or honduras or hungary or india or indonesia or timor or iran or iraq or isle of man or jamaica or jordan or kazakhstan or kazakh or kenya or "democratic people’s republic of korea" or republic of korea or north korea or south korea or korea or kosovo or kyrgyzstan or kirghizia or kirgizstan or kyrgyz republic or kirghiz or laos or lao pdr or "lao people's democratic republic" or latvia or lebanon or lebanese republic or lesotho or basutoland or liberia or libya or libyan arab jamahiriya or lithuania or macau or macao or republic of north macedonia or macedonia or madagascar or malagasy republic or malawi or nyasaland or malaysia or malay federation or malaya federation or maldives or indian ocean islands or indian ocean or mali or malta or micronesia or federated states of micronesia or kiribati or marshall islands or nauru or northern mariana islands or palau or tuvalu or mauritania or mauritius or mexico or moldova or moldovian or mongolia or montenegro or morocco or ifni or mozambique or portuguese east africa or myanmar or burma or namibia or nepal or netherlands antilles or nicaragua or niger or nigeria or oman or muscat or pakistan or panama or papua new guinea or new guinea or paraguay or peru or philippines or philipines or phillipines or phillippines or poland or "polish people's republic" or portugal or portuguese republic or puerto rico or romania or russia or russian federation or ussr or soviet union or union of soviet socialist republics or rwanda or ruanda or samoa or pacific islands or polynesia or samoan islands or navigator island or navigator islands or "sao tome and principe" or saudi arabia or senegal or serbia or seychelles or sierra leone or slovakia or slovak republic or slovenia or melanesia or solomon island or solomon islands or norfolk island or norfolk islands or somalia or south africa or south sudan or sri lanka or ceylon or "saint kitts and nevis" or "st. kitts and nevis" or saint lucia or "st. lucia" or "saint vincent and the grenadines" or saint vincent or "st. vincent" or grenadines or sudan or suriname or surinam or dutch guiana or netherlands guiana or syria or syrian arab republic or tajikistan or tadjikistan or tadzhikistan or tadzhik or tanzania or tanganyika or thailand or siam or timor leste or east timor or togo or togolese republic or tonga or "trinidad and tobago" or trinidad or tobago or tunisia or turkey or turkmenistan or turkmen or uganda or ukraine or uruguay or uzbekistan or uzbek or vanuatu or new hebrides or venezuela or vietnam or viet nam or middle east or west bank or gaza or palestine or yemen or yugoslavia or zambia or zimbabwe or northern rhodesia or global south or africa south of the sahara or sub-saharan africa or subsaharan africa or africa, central or central africa or africa, northern or north africa or northern africa or magreb or maghrib or sahara or africa, southern or southern africa or africa, eastern or east africa or eastern africa or africa, western or west africa or western africa or west indies or indian ocean islands or caribbean or central america or latin america or "south and central america" or south america or asia, central or central asia or asia, northern or north asia or northern asia or asia, southeastern or southeastern asia or south eastern asia or southeast asia or south east asia or asia, western or western asia or europe, eastern or east europe or eastern europe or developing country or developing countries or developing nation? or developing population? or developing world or less developed countr* or less developed nation? or less developed population? or less developed world or lesser developed countr* or lesser developed nation? or lesser developed population? or lesser developed world or under developed countr* or under developed nation? or under developed population? or under developed world or underdeveloped countr* or underdeveloped nation? or underdeveloped population? or underdeveloped world or middle income countr* or middle income nation? or middle income population? or low income countr* or low income nation? or low income population? or lower income countr* or lower income nation? or lower income population? or underserved countr* or underserved nation? or underserved population? or underserved world or under served countr* or under served nation? or under served population? or under served world or deprived countr* or deprived nation? or deprived population? or deprived world or poor countr* or poor nation? or poor population? or poor world or poorer countr* or poorer nation? or poorer population? or poorer world or developing econom* or less developed econom* or lesser developed econom* or under developed econom* or underdeveloped econom* or middle income econom* or low income econom* or lower income econom* or low gdp or low gnp or low gross domestic or low gross national or lower gdp or lower gnp or lower gross domestic or lower gross national or lmic or lmics or third world or lami countr* or transitional countr* or emerging economies or emerging nation?).ti,ab,hw. (3684931)

2 (afghan or afghans or afghani or albanian? or algerian? or american samoan? or angolan? or antiguan? or barbudan? or argentine? or argentinian? or argentinean? or armenian? or aruban? or azerbaijani? or bahraini? or bangladeshi? or bangalees or bajan? or belarusian? or byelorussian? or belizean? or beninese? or bhutanese or bolivian? or bosnian? or botswana or batswana or brazilian? or brasilian? or bulgarian? or burkinabe or burkinese or burundian? or cape verdean? or cabo verdean? or cambodian? or khmer or cameroonian? or central african? or chadian? or chilean? or chinese or colombian? or comorian? or congolese or costa rican? or ivorian? or croatian? or cuban? or cypriot? or czech? or djiboutian? or dominican? or ecuadorian? or egyptian? or salvadoran? or equatorial guinean? or equatoguinean? or eritrean? or estonian? or swazi? or swati? or ethiopian? or fijian or gabonese or gabonaise or gambian? or georgian? or ghanaian? or gibraltarian? or greek? or grenadian? or guamanian? or guatemalan? or guinean? or bissau guinean? or guyanese or haitian? or honduran? or hungarian? or indian? or indonesian? or iranian? or iraqian? or iraqi? or manx or jamaican? or jordanian? or kazakhstani? or kenyan? or kirabati or kirabatian? or north korean? or korean? or kosovar? or kosovan? or kyrgyz* or lao or laotian? or latvian? or lebanese or lesothan? or lesothonian? or mosotho or basotho or liberian? or libyan? or lithuanian? or macanese or macedonian? or malagasy or madagascan? or malawian? or malaysian? or maldivian? or malian? or maltese or marshallese? or mauritanian? or mauritian? or mexican? or micronesian? or moldovan? or mongolian? or mongol or montenegrin? or moroccan? or mozambican? or burmese or myanma or namibian? or nauruan? or nepali or nepalese or netherlands antillean? or nicaraguan? or nigerien? or nigerian? or northern mariana islander? or mariana? or omani? or pakistani? or palauan? or panamanian? or papua new guinean? or paraguayan? or peruvian? or philippine? or philipine? or phillipine? or phillippine? or filipino? or filipina? or polish or pole or poles or portuguese or puerto rican? or romanian? or russian? or soviet people or soviet population or rwandan? or rwandese or ruandan? or ruandese or samoan? or sao tomean? or santomean? or saudi arabian? or saudi? or senegalese or serbian? or montenegrin? or seychellois or seychelloise? or sierra leonean? or slovak? or slovene? or solomon islander? or somali? or south african? or south sudanese or sri lankan? or ceylonese or kittitian? or nevisian? or saint lucian? or vincentian? or sudanese or surinamese? or syrian? or tajik? or tajikistani? or tanzanian? or tanganyikan? or thai or timorese? or togolese or tongan? or trinidadian? or tobagonian? or tunisian? or turk? or turkish or turkmen? or tuvaluan? or ugandan? or ukrainian? or uruguayan? or uzbek? or vanuatu* or venezuelan? or vietnamese or yemeni? or yemenite? or yemenese or yugoslav? or yugoslavian? or zambian? or zimbabwean? or african? or asian? or pacific islander? or latin american? or central american? or south american? or caribbean? or west indian? or iberoamerican? or middle eastern?).ti,ab,hw. (1437850)

3 1 or 2 (3958566)

4 ((match* adj2 (propensity or coarsened or covariate or neighbo?r)) or "propensity score" or ("difference* in difference*" or "difference-in-difference*" or "differences-in-difference*" or "double difference*") or (quasi-experiment$2 or "quasi experiment$2") or (estimator and evaluat*) or ("instrumental variable*" or (IV adj2 (estimation or approach))) or (Heckman adj3 (model* or approach*)) or ((two-stage or "two stage" or three-stage or "three stage" or four-stage or "four stage") adj3 (control* or function* or regression* or "least squares")) or 2SLS or "regression discontinuity" or "synthetic control?" or "time series" or counterfactual or "segment* regression" or (non adj2 participant*) or ((control or comparison) adj2 (group* or condition* or area* or village* or household* or intervention)) or (panel$1 adj2 (data or household* or model*)) or ((exploit* or "tak* advantage") adj3 (variation* or variety or exogen* or heterogen*)) or (econometric adj2 (model* or adjust*)) or (select* adj2 (bias* or self))).ti,ab,hw. (281868)

5 ((experiment$4 adj2 (design or study or research or evaluat* or evidence or vary or varies or variation)) or ((random or randomi#ed or randomly) adj2 (trial or assign* or treatment or control* or allocat* or experiment$2 or evaluat* or vary or varies or variation or choose or chose*))).ti,ab,hw. (216489)

6 ((impact? or effect*) adj2 (evaluat* or assess or assessing or assessment or analyze or analyse or analyzing or analysing or analysis or analytical or estimate or estimating or estimation or cause or causal)).ti,ab,hw. (295222)

7 ("program* evaluation" or "project evaluation" or "evaluation research" or "natural experiment*" or "program* effectiveness" or "outcome assessment" or "evaluation study" or "field experiment").ti,ab,hw. (77660)

8 4 or 5 or 6 or 7 (779554)

9 ((Systematic* or synthes*) adj3 (research or evaluation* or overview or finding* or thematic* or report or descriptive or explanatory or narrative or meta* or review* or data or literature or studies or evidence or map or mapping or quantitative or study or studies or paper or impact or impacts or effect* or compar*)).ti,ab,hw. (99191)

10 ("Meta regression" or "meta synth*" or "meta-synth*" or "meta analy*" or "metaanaly*" or "meta-analy*" or "metanaly*" or "Metaregression" or "Meta-regression" or "Methodologic* overview" or "pool* analys*" or "pool* data" or "Quantitative* overview" or "research integration").ti,ab,hw. (42184)

11 ((effectiveness or effects or systemat* or synth* or integrat* or gap or methodologic* or quantitative or evidence or literature or rapid or scoping) adj3 (review or map)).ti,ab,hw. (92711)

12 9 or 10 or 11 (170119)

13 3 and 8 (294697)

14 3 and 12 (52379)

15 13 or 14 (339527)

16 (Bioenergy or biofuel* or Biomass* or coal or electricity or electrificat* or energy or fuel or gas or geothermal* or hydro or hydroelectric* or hydropower or nonrenewable* or oil or power or renewable* or solar or wave or water or wind).tw. (2700994)

17 exp renewable energy/ or exp biofuels/ or exp water power/ or exp wave power/ or exp wind power/ (99027)

18 16 or 17 (2702338)

19 (audit* or access* or adequa* or affordab* or alternative* or availab* or capacity or clean or connect* or conservation or consumer* or consump* or cook* or cool* or coverage or credit* or delivery or demand* or design* or development* or dissemination or distribut* or efficien* or end-user* or Enforcement* or expansion or Financ* or framework* or generat* or grid or grids or heat* or improv* or incentiv* or information* or infrastructur* or insurance or Invest* or light* or loan* or maintenance or market* or micro or monitor or network* or outage* or performance or planning or policies or policy or power or privat* or production or pre-paid or program* or project* or provision* or quality or reform* or regulat* or reliab* or renewable or report* or resource* or sector* or service* or source* or standard* or storage or subsid* or supply* or supplies or supplier* or sustainab* or Target* or tariff* or technolog* or training or transmission or system* or usage or utility or workshop*).tw. (10185979)

20 "use".tw. (1461408)

21 (system* adj1 maintenanc*).tw. (615)

22 (system* adj1 management).tw. (31102)

23 19 or 20 or 21 or 22 (10254515)

24 15 and 17 (2116)

25 15 and 16 and 23 (93660)

26 24 or 25 (93695)

## Grey Literature Example:

Deutsche Gesellschaft für Internationale Zusammenarbeit (GIZ)

Search link: <https://mia.giz.de/esearcha/browse.tt.html>

Publication year: from 2000 to 2023

AND

Subject keywords: energy

OR

Classification: Energie

Online resources only: Yes

The Academy's Media and Information Centre (MIA) archives: Yes

GIZ publications: Yes

Type: Article/Part; Book; Dissertation; Hochschulschrift (university thesis); Journal; Report; Research report; Zeitschriftenartikel (magazine/journal article)

Results: 88

# Appendix D: Data extraction forms

## D.1: Study characteristics form

| **Code** | **Subcode** |
| --- | --- |
| Study Information | Study ID |
|  | Coder name |
|  | Title |
|  | Foreign Title |
|  | Short title |
|  | Language |
| Author Information | Author Name |
|  | Author Affiliation Institution |
|  | Author Affiliation Country |
| Publication Information | Publication Type |
|  | DOI |
|  | Study status |
|  | Abstract |
|  | Keywords |
|  | Journal name |
|  | Other journal name |
|  | Journal volume |
|  | Journal issue |
|  | Pages |
|  | Year of Publication |
|  | URL |
|  | Publisher location |
|  | Open access |
| Sector Information | Sector name |
|  | Sub-sector name |
|  | DAC rank |
|  | Primary DAC Code |
|  | Secondary DAC Code |
|  | CRS-Voluntary (tertiary) Code |
|  | SDGs |
|  | World Bank (WB) first theme |
|  | WB first sub-theme |
|  | WB second theme |
|  | WB second sub-theme |
|  | WB third theme |
|  | WB third sub-theme |
|  | Other topics |
|  | Equity focus |
|  | Equity dimension |
|  | Equity description |
| Geographic Information | First year of intervention |
|  | Continent name |
|  | Country name |
|  | Additional country |
|  | Country income level |
| Target population | Equity population |
| Methodological information | Evaluation Design |
|  | Evaluation Method |
|  | Mixed Method |
|  | Additional quantitative Methods |
|  | Unit of Observation |
| Program, Funding and Implementation Information | Project Name |
|  | Implementation Agency Category |
|  | Implementation Agency Name |
|  | Program Funding Agency Category |
|  | Program Funding Agency Name |
|  | Research Funding Agency Category |
|  | Research Funding Agency Name |
| Intervention Information | Treatment group/Arm 1 |
|  | Treatment group/Arm 1 Description |
|  | Intervention group/Arm 2 |
|  | Treatment group/Arm 2 Description |
|  | (Create additional options as necessary) |
| Outcome Information | Outcome |
|  | Outcome description |
|  | (Create additional options as necessary) |
| Energy-relevant/ EGM categories | SDG 7 Target: access; renewables; efficiency |
|  | Energy Source (aggregated): non-renewable; modern renewable; traditional renewable; renewable (general), not specified; not applicable^[[3]](#footnote-4)^ |
|  | Energy Source (disaggregated): coal; solar; wind; geothermal; hydroelectric; biomass; other; not specified; not applicable; liquified petroleum gas (LPG); wood; charcoal; liquid biofuel; biogas; gasoline or diesel; natural gas |
|  | Energy Use: cooking; lighting; heating; cooling; productive – agriculture; productive – other; not specified; not applicable; travel; education; health |
|  | Technology: improved cookstoves; engines; solar lighting; heat pumps; insulation; energy efficient windows; hydropower plants; wind plants; biodigesters; vehicles; other; not specified; not applicable; lightbulbs; air conditioners |
|  | Supply/demand-side: supply; demand; both; not specified |
|  | Target level: individuals; individual traders; households; communities; firms/ enterprises; utility providers; national governments; not specified; other; agricultural producers; schools; health workers |
|  | Population targeted: youth; caste; conflict-affected; disability; education; ethnicity; head of household; HIV/AIDS; internally displaced people; land size; land ownership; rural; religion; refugees; socioeconomic status; women; sexual orientation; sexual identity; other; urban; men; older person |

## D.2: Quantitative extraction form

| **Variable group** | **Variable** | **Description** |
| --- | --- | --- |
| Publication Information | Study ID | The unique ID code that is assigned to each included study |
|  | Estimate ID | The unique ID code that assigned to each individual estimate |
|  | Study status | Select one of the following:  i) Completed; ii) Protocol; iii) Ongoing |
|  | Author Name | Authors last names [Open Answer] |
|  | Title | Title of the study |
|  | Year of Publication | Year published (publication date, not preprint or first online publication dates) |
| Intervention Information | Country | Country of intervention |
|  | Project/ policy name | If the intervention has an explicit name, code this here. If there is no explicit name, leave blank. |
|  | Intervention mechanism | Select the intervention mechanism from the following list. Separate treatment arms should be inputted in separate rows. |
|  | Intervention technology | Select the intervention technology from the following list. Separate treatment arms should be inputted in separate rows. |
|  | Non-staggered intervention | Have the treated observations been exposed to the intervention for the same amount of time? 1=Yes; 0=No |
|  | Year of the intervention | The earliest date (year) observations are exposed to the intervention. |
|  | Length of follow up | How many months have elapsed between the start of the intervention (earliest date observations are exposed to the intervention) and the date of the final outcome measurement. If less than one month, use decimals (e.g. one week would be .25, etc.).   If no month is mentioned for the time of the intervention start and/or the last date of the outcome measurement, consider as a full year. So if project started in 1986 and last data collection was in 2010 (with no information on the month for both), answer 25*12=300  If no information provided in the study and have an intervention name, do a quick search on the internet to find out when the intervention started and input here. |
|  | Evaluation period (in months) | The total number of months elapsed between the end of an intervention and the point at which an outcome measure is taken post intervention, or as a follow-up measurement.  If less than one month, use decimals (e.g., measurement immediately after the intervention end would be coded as 0, one week would be .25, etc.) |
| Method information | Evaluation Design | Select one of the options below:  1. Experimental (defined as prospective randomised assignment, where randomisation is implemented by researchers (or by decision makers in the context of an evaluation study)  2. Quasi-experimental (including natural experiments and non-randomised studies). |
|  | Evaluation Method | If Experimental, then select:  Randomised controlled trial  If Quasi-experiment or natural experiment, then select:  Natural experiment in which exposure to treatment is random  Regression Discontinuity Design (RDD)  Difference-in-Differences (DID) / Fixed effects estimation  Instrumental variable (IV) estimation  Endogenous treatment-effects models (including endogenous switching regression, and other methods synonymous to the Heckman two step model)  Statistical matching (includes PSM or statistical weighting)  Interrupted time series (ITS)  Synthetic controls |
|  | Study population | Provide any details in the paper that describe how the study population was selected, covering:  a) How is the population selected? what is the sampling strategy to recruit participants from that population into the study?  b) What are the characteristics of study participants?  Targeted population appears first in the cell. Explanations can follow afterwards Need to add all specified information in the study on the targeted population: subsistence farmers, commercial farmers, ... (look in data section). |
|  | Additional Methods | Select additional method if any. If none, select not applicable. [Open Answer] |
| Estimate  Information | Analysis type for this effect size | Free text, what type of analysis was used (Regression, 2SLS, ANCOVA, etc.) |
|  | Estimate Type | Type of data for this effect size: 1 = Continuous - means and SDs, 2 = Continuous - mean difference and SD, 3 = Dichotomous outcome - proportions, 4 = Regression data - dichotomous outcome, 5 = Regression data - continuous outcome |
|  | Treatment Effect | 1=Intention to Treat (ITT), 2=Average Treatment Effect on the Treated (ATET), 3=Average Treatment Effect (ATE) 4 = Local Average Treatment Effect (LATE) |
|  | Unit of analysis | What is the unit of analysis? UOA for this effect size: 1= Individual, 2= Household, 3= Group (e.g., community organisation), 4= Village, 5 = Other, 6 = Not clear |
|  | Source | Note the page number, table number, column, and row you used to extract the data  [Open Answer] |
| Outcome information | Outcome group | Select the relevant outcome group from the dropdown list. |
|  | Outcome category | Select the relevant outcome category from the list below. |
|  | Outcome | Select the relevant outcome from the list below. |
|  | Outcome indices | Select the relevant outcome indices from the list below. |
|  | Outcome measurement | How was the data collected? 1=Self-reported, 2=Administrative data, 3=Satellite data |
|  | Outcome type | Record the type of outcome variable: 1=Continuous; 2=Discrete (including proportions); 3=Nominal (binary); 4=Ordinal (binary); 5=Nominal (non-binary); 6=Ordinal (non-binary); 7=Interval. |
|  | Levels or changes | 0 = Unit is the level of outcome variable, 1 = Change in outcome variable |
|  | Reverse sign | Record no=0 if an increase is good, record yes=1 if a decrease is good and the sign needs to be reversed (i.e., decrease is good) |
|  | Outcome dataset | Record if data for this outcome comes from an identified dataset |
| Treatment variable information | Treatment | Record the treatment variable as written in the model (e.g., the variable name the author uses, such as ("Intervention x Time")  [Open Answer] |
|  | Treatment type | Describe the types of treatment variable used: i) binary; ii) continuous; iii) categorical; iv) other |
|  | Comparison | 1=No intervention (service delivery as usual), 2=Other intervention, 3=Pipeline (waitlist) control (still service delivery as usual) |
|  | Describe Comparison Group | Describe the comparison group [Open Answer] |
|  | Subgroup | Is this analysis of a subgroup or estimating heterogeneous effects?  0=no, 1=yes |
|  | Subgroup information | Describe the subgroup or variable interacted with the treatment variable if applicable (e.g., boys, girls).  If no subgroup, select not applicable [Open Answer] |
| Estimate data | Mean treatment | Outcome mean for the treatment group |
|  | SD treatment | Outcome standard deviation for treatment group |
|  | Mean Control | Outcome mean for the comparison group |
|  | SD Control | Outcome standard deviation for control group |
|  | Mean difference | Overall mean difference (treatment - control) |
|  | SE difference | Standard error of the overall mean difference |
|  | Tstat difference | t-statistic of mean difference |
|  | p-value difference | p-value of mean difference |
|  | Odds ratio | Odds ratio reported in the study |
|  | SE odds ratio | Odds ratio standard error reported in the study |
|  | Risk ratio | Risk ratio reported in study |
|  | SE risk ratio | Risk ratio standard error |
|  | Coeff reg | Report the regression coefficient of the treatment effect |
|  | SE reg | Report the associated standard error of the regression coefficient. |
|  | Tstat reg | Report the associated t statistic of the effect size (coefficient/SE) |
|  | CI_LB reg | Report the associated Lower bound of the 95% Confidence interval of the effect size. If CI is reported for a different confidence level, indicate that in the notes section. |
|  | CI_UP reg | Report the associated Upper bound of the 95% Confidence interval of the effect size. If CI is reported for a different confidence level, indicate that in the notes section. |
|  | P value exact | Exact p value if given, if not, record as written in the manuscript (e.g., p < .001, or p > .05) |
|  | Clusters treatment | Number of clusters - treatment group |
|  | Clusters control | Number of clusters -  control group |
|  | Clusters total | Number of clusters - total sample |
|  | N treatment | Sample size - treatment group |
|  | N control | Sample size - control group |
|  | N total | Sample size - total sample |
|  | periods (1 if cross sectional) | Record how many time-period there are in the evaluation (e.g., cross section is 1, panel data with 3 measurements is 3) |
|  | Does the sample size need to be corrected? | Often in panel data, models will report number of observations rather than number of participants. In this column you will indicate 1="Yes" if the sample size needs to be divided by the number of periods, and 0="No" if either it is cross-sectional data, or if the authors have already divided the number of observations by the number of panel assessments and thus no correction is necessary. |

# Appendix E: Risk of bias tools

## E.1: Quantitative risk of bias tool

| Question | Coding format | Criteria | Decision rule |
| --- | --- | --- | --- |
| General | | | |
| Study ID | EPPI ID |  |  |
| Estimate ID | EPPI ID Estimate # |  |  |
| Study first author | Open answer |  |  |
| Time taken to complete assessment | Minutes |  |  |
| Design type: What type of study design is used? | 1= Randomised controlled trial (RCT) (random assignment to households/individuals) or quasi-RCT 2= Cluster-RCT (quasi-RCT) |  |  |
| Methods used for analysis: Which methods are used to control for selection bias and confounding? | 1 = Statistical matching (PSM, CEM, covariate matching) 2 = Difference in differences (DID) estimation methods + Fixed effect 3 = IV-regression (2-stage least squares or bivariate probit) 4 = Heckman selection model 5 = Covariate adjusted estimation / cross-section 6 = Propensity weighted regression 7 = Comparison of means 8 = Other (please state) |  |  |
| Design and analysis method description | Open answer | Briefly describe the study design and analysis method undertaken by the authors. Take content from QEX. |  |
| Study registration | Open answer | Provide link to pre-analysis plan if available. |  |
| 1: Assignment mechanism - Assessment | | | |
| Assignment mechanism: Was the allocation or identification mechanism random or as good as random? | 1= Yes,  2 = Probably Yes,  3 = Probably No,  4 = No,  8 = Unclear | a) The authors describe a random component in sequence generation/ randomization method (e.g. lottery, coin toss, random number generator) and assignment is performed for all units at the start of the study centrally or using a method concealed from participants and intervention delivery. b) If public lottery is used for the sequence generation, authors provide detail on the exact settings and participants attending the lottery. c) If a special randomization procedure is used to ensure balance, it is well described and justified given the study setting (stratification, pairwise matching, unique random draw, multiple random draws etc). d) A balance table is reported suggesting that allocation was random between all groups including subgroup receiving different treatment within control or treatment groups (if the comparison is relevant for this assessment). | Score “Yes” if all criterion a), b), c) and d) are satisfied. Score "Probably Yes" if only criterion a) and b) are not satisfied OR if only criteria c) is not satisfied. Score “Unclear” if d) is not satisfied because no balance table is reported. Score "Probably No" if d) is not satisfied because there is no balance table reported and there is evidence suggesting a problem in the randomization, such as baseline coefficients in a diff-in-diff regression table are very different or sample size is too small for the procedure used (using stratification when there are less than two units for each intervention and control group in each strata can lead to imbalance). Score “No” if d) is not satisfied because there are large imbalances concerning a large number of variables, providing evidence that the assignment was not random. If this is scored as no, use the NRS tool. |
| Assignment justification | Open answer. Your answer should include all points here (a, b, c), the justification for each point (cite parts from the study) and a page number. a, Yes as... p.XX b No as...p.XX c Yes as... p.XX | Justification for coding decision (Include a brief summary of justification for rating, mentioning your response to all sub questions, cite relevant pages). |  |
| 2: Unit of analysis - Assessment | | | |
| Unit of analysis: Is unit of analysis in cluster allocation addressed in standard error calculation ? | 1=Yes 2=No 3=Not reported/unclear 4=Not applicable |  | Score "Yes" if Unit of analysis (UoA) = Unit of randomisation (UoR) OR if UoA ≠ UoR and standard errors are clustered at the UoR level OR data is collapsed to the UoR level Score "Not reported/unclear" if not enough information is provided on the way the standard errors were calculated or what the unit of analysis is. Score "Not applicable" if it is not a cluster RCT. Score "No" otherwise. |
| Method used to address differences between UoA and unit of data collection | Open answer |  |  |
| 3: Selection bias – Assessment | | | |
| Selection bias Was any differential selection into or out of the study (attrition bias) adequately resolved? | 1= Yes,  2 = Probably Yes,  3 = Probably No,  4 = No,  8 = Unclear |  | Score "Yes" if there is no attrition or attrition falls into the green zone (see figure A.1 below) and the study establishes that attrition is randomly distributed (e.g. by presenting balance by key characteristics across groups) AND if survey respondents were randomly sampled. Score "Probably yes" if attrition falls into the green zone AND if survey respondents were randomly sampled. Score "Unclear" if there is an attrition problem but no information provided on the relationship between attrition and treatment status, OR if there is not enough information on how the population surveyed was sampled. Score "Probably no" if there is attrition which is likely to be related to the intervention OR there is some indication that the survey respondents were purposely sampled in a way that might have led the sampling to be different between treatment and control groups, or attrition falls into the yellow zone. Score "No" if attrition falls into the red zone. USE ATTRITION GRAPH TAB TO DETERMINE THE ATTRITION ZONE |
| Selection bias justification | Open answer | Justification for coding decision (Include a brief summary of justification for rating, mentioning your response to all sub questions, cite relevant pages). |  |
| 4: Confounding – Assessment | | | |
| Confounding and group equivalence: Was the method of analysis executed adequately to ensure comparability of groups throughout the study and prevent confounding | 1= Yes,  2 = Probably Yes,  3 = Probably No,  4 = No,  8 = Unclear | a) Baseline characteristics are similar in magnitude; b) Unbalanced covariates at the individual and cluster level are controlled in adjusted analysis; c) Adjustments to the randomization were taken into account in the analysis (stratum fixed effects, pairwise matching variables)? (Bruhn and McKenzie 2009) | Score “Yes” if criterion a) and b) are satisfied; Score "Probably yes" if a) is not satisfied but b) is satisfied and imbalances are small in magnitude OR if only a) is satisfied.  Score “Unclear” if no balance table is provided or if imbalances are controlled for but they are very large in magnitude and assignment mechanism is not coded as "Yes" or "Probably yes" Score "Probably no" if a) and b) are not satisfied and the magnitude of imbalances are small Score “No” if a) and b) are not satisfied and the magnitude of imbalances are large and covariates are clear determinant of the outcomes. |
| Confounding justification | Open answer | Justification for coding decision (Include a brief summary of justification for rating, mentioning your response to all sub questions, cite relevant pages). |  |
| 5: Deviations from intended interventions – Assessment | | | |
| Deviations from intended interventions: Spill-overs, cross-overs and contamination: was the study adequately protected against spill-overs, cross-overs and contamination? | 1= Yes,  2 = Probably Yes,  3 = Probably No,  4 = No,  8 = Unclear | a) There was no implementation issues that might have led the control participants to receive the treatment (implementer's mistake). b) The intervention is unlikely to spill-over to comparisons (e.g. participants and non-participants are geographically and/or socially separated from one another and general equilibrium effects are not likely) or the potential effects of spill overs were measured (e.g. variation in the % of unit within a cluster receiving the treatment). c) There is no risk of contamination by external programs: the treatment and comparisons are isolated from other interventions which might explain changes in outcomes.  d) There is nothing in the surveys that might have given the control participants an idea of what the other group might receive OR they did but there is no risk that this has changed their behaviours; AND the survey process did not reveal information to the control group that they did not have before (e.g. the study aims to measure increase in take up of a service or product that participants might not know about) Authors might put something in place in the design of the study that allows to control for that survey effect (e.g. a pure control with no monitoring except baseline end line) | Score “Yes” if criterion a), b), c) and d) are satisfied; Score "Probably yes" if there is no obvious problem but there is no information reported on potential risks related to spill overs, contamination, or survey effects in the control group OR if there were issues with spill-overs but they were controlled for or measured. Score “Unclear” if spill-overs, cross-overs, survey effects and/or contamination are not addressed clearly. Score "Probably no" if any of the criterion a), b), c) or d) are not satisfied but the scale of the issue is not clear. Score “No” if any of the criterion a), b), c) or d) are not satisfied and happened at a large scale in the study. |
| Deviations justification | Open answer. Your answer should include all points here (a, b, c), the justification for each point (cite parts from the study) and a page number. a, Yes as... p.XX b No as...p.XX c Yes as... p.XX | Justification for coding decision (Include a brief summary of justification for rating, mentioning your response to all sub questions, cite relevant pages).  For example, intervention groups are geographically separated, authors use intention to treat estimation or instrumental variables to account for non-adherence, and survey questions are not likely to expose individuals in the control group to information about desirable behaviours (‘survey effects’). |  |
| 6. Performance bias - Assessment | | | |
| Performance bias: Was the process of monitoring individuals unlikely to introduce motivation bias among participants? | 1= Yes,  2 = Probably Yes,  3 = Probably No,  4 = No,  8 = Unclear | a) The authors state explicitly that the process of monitoring the intervention and outcome measurement is blinded and conducted in the same frequency for treatment and control groups, or argue convincingly why it is not likely that being monitored could affect the performance of participants in treatment and comparison groups in different ways (such as resulting in Hawthorne or John Henry effects).  b) The outcome is based on data collected in the context of a survey, and not associated with a particular intervention trial, or data are collected from administrative records or in the context of a retrospective (ex post) evaluation. | Score “Yes” if either criterion a) or b) are satisfied; Score "Probably yes" if the study is based on data collected during a trial and there is no obvious issue with the monitoring processes but authors do not mention potential risks. Score “Unclear” if it is not clear whether the authors use an appropriate method to prevent Hawthorne and John Henry Effects (e.g. blinding of outcomes and, or enumerators, other methods to ensure consistent monitoring across groups). Hawthorne effects may result where participants know that they are being observed and John Henry Effects may result from participant knowledge of being compared. Score "Probably no" if there was imbalance in the frequency of monitoring in intervention groups, which might have influenced participants' behaviours. Score "No" if neither criterion a) or b) are satisfied. |
| Performance bias justification | Open answer. Your answer should include all points here (a, b, c), the justification for each point (cite parts from the study) and a page number. a, Yes as... p.XX b No as...p.XX c Yes as... p.XX | Justification for coding decision (Include a brief summary of justification for rating, mentioning your response to all sub questions, cite relevant pages). |  |
| 7. Outcome measurement bias - Assessment | | | |
| Outcome measurement bias: Was the study free from biases in outcome measurement? | 1= Yes,  2 = Probably Yes,  3 = Probably No,  4 = No,  8 = Unclear | a) Outcome assessors are blinded or the outcome measures are not likely to be biased by their judgement.  b) For self-reported outcomes: respondents in the intervention group are not more likely to have accurate answers due to recall bias; c) For self-reported outcomes: respondents do not have incentives to over/under report something related to their performance or actions, OR researchers put in place mechanisms to reduce the risk of reporting bias (researchers not strongly involved in the implementation of the program and it is clear that their answers to the survey will not affect what they receive in the future) OR authors have measured the risks of bias through falsification tests or measuring the effect on placebo outcomes in cases where there was a risk of reporting bias. d) Timing issue: the data collection period did not differ between intervention and comparison group, the baseline data is not likely to be affected by the beginning of the intervention or affects a small percentage of the study participants. | Score “Yes” if criterion a), b), c) and d) are satisfied: Score "Probably yes" if there is a small risk related to any of a), b), c) or d) and there is no more information provided to to justify the absence of bias OR if there was a high risk of bias but authors have either controlled it in their design or measured it with a placebo outcomes. Score “Unclear” if it there is a high risk related to any of a), b), c) or d) and there is no more information provided to to justify the absence of bias.  Score "Probably no" if there are high risk related to a), b), c) or d) and it is clear that authors were not able to control for this bias. Score “No” if there is evidence of bias. |
| Outcome measurement justification | Open answer. Your answer should include all points here (a, b, c), the justification for each point (cite parts from the study) and a page number. a, Yes as... p.XX b No as...p.XX c Yes as... p.XX | Justification for coding decision (Include a brief summary of justification for rating, mentioning your response to all sub questions, cite relevant pages). |  |
| 8. Reporting bias - Assessment | | | |
| Analysis reporting: Was the study free from selective analysis reporting? | 1= Yes,  2 = Probably Yes,  3 = Probably No,  4 = No,  8 = Unclear | a) A pre-analysis plan or trial protocol is published and referred to or the trial was pre-registered or the outcomes were pre-registered; b) Authors report results corresponding to the outcomes announced in the pre-analysis plan. If PAP is not available, the authors report results corresponding to the methods section (there is no evidence of outcome reporting bias); c) Authors report results of unadjusted analysis and intention to treat (ITT) estimation, alongside any adjusted and treatment-on-the-treated/complier-average-causal-effects analysis.) d) Authors use the appropriate analysis method (use baseline data when available) and different treatment arms are differentiated in the analysis e) Authors have reported all the analysis which could help understand the results and no other bias is assessed as unclear due to the lack of an important analysis (e.g. a balance table or a subgroup analysis) | Score "Yes" if all the criterion a), b), c), d), and e) are satisfied; Score "Probably yes" if all the conditions are met except a), or if all the conditions are met but there is some element missing that could have helped understand the results better (e); Score "Unclear" if there is not enough information to determine that there is an analysis missing; Score "Probably no" if any of the criterion b), c) or d) are not satisfied; Score "No" if any of the criterion b), c) or d) are not satisfied and there is evidence that the analysis results would be different because large imbalances were not controlled for, compliance was very low and ITT estimation was not reported or different treatment arms were pooled. |
| Analysis reporting justification | Open answer. Your answer should include all points here (a, b, c), the justification for each point (cite parts from the study) and a page number. a, Yes as... p.XX b No as...p.XX c Yes as... p.XX | Justification for coding decision (Include a brief summary of justification for rating, mentioning your response to all sub questions, cite relevant pages). |  |

## E.2: Qualitative risk of bias tool

| Study type | **Methodological appraisal criteria** | | | | | | | | | | | | | | | **Response** | | | | |
| --- | --- | --- | --- | --- | --- | --- | --- | --- | --- | --- | --- | --- | --- | --- | --- | --- | --- | --- | --- | --- |
|  |  |  |  |  |  |  |  |  |  |  |  |  |  |  |  | Yes | | No | Comment | |
| *Screening questions: assessing* *‘fatal flaws’* | Configurative assessment:   - Study reports primary data and applied methods - Study states clear research questions and objectives - Study states clear research design, which is appropriate to address the stated research question and objectives (*Purposivity*) - The findings of the study are based on collected data, which justify the knowledge claims (*Accuracy*) | | | | | | | | | | | | | | |  | |  |  | |
|  | ***Screening question based on abstract and/or superficial reading of*** ***full text: Further appraisal is not feasible or appropriate when the answer is ‘No’ to any of the above screening questions!*** | | | | | | | | | | | | | | | | | | | |
| Study type | **Methodological appraisal criteria** | | | | | | | | | | | | | | | **Response** | | | | |
|  |  |  |  |  |  |  |  |  |  |  |  |  |  |  |  | Yes | | No | Comment / Confidence judgment | |
| *1. Qualitative and descriptive quantitative, and process evaluations* | 1. **RESEARCH IS DEFENSIBLE IN DESIGN (**providing a research strategy that addresses the question)   Appraisal indicators:   - *Is the research design clearly specified and appropriate for aims and objectives of the research?*   Consider whether | | | | | | | | | | | | | | |  | |  |  | |
|  | 1. *there is a discussion of the rationale for the study design* | | | | | | | | | | | | | | |  | |  |  | |
|  | 1. *the research question is clear, and suited to the inquiry* | | | | | | | | | | | | | | |  | |  |  | |
|  | 1. *there are convincing arguments for different features of the study design* | | | | | | | | | | | | | | |  | |  |  | |
|  | 1. *limitations of the research design and implications for the research evidence are discussed* | | | | | | | | | | | | | | |  | |  |  | |
|  | **Defensible** | **Arguable** | | | **Critical** | | | | | | | | | | **Not defensible** | *Worth to continue:* | | | | |
|  |  | | | | | | | | | | | | | | | | | | | |
|  | 1. **RESEARCH FEATURES AN APPROPRIATE SAMPLE (**following an adequate strategy for selection of participants)   Appraisal indicators:  Consider whether | | | | | | | | | | | | | | |  | |  |  | |
|  | 1. *there is a description of study location and how/why it was chosen* | | | | | | | | | | | | | | |  | |  |  | |
|  | 1. *the researcher has explained how the participants were selected* | | | | | | | | | | | | | | |  | |  |  | |
|  | 1. *the selected participants were appropriate to collect rich and relevant data* | | | | | | | | | | | | | | |  | |  |  | |
|  | 1. *reasons are given why potential participants chose not take part in study* | | | | | | | | | | | | | | |  | |  |  | |
|  | **Appropriate sample** | | **Functional sample** | | | | | **Critical sample** | | | | | | **Flawed sample** | | *Worth to continue:* | | | | |
|  |  | | | | | | | | | | | | | | | | | | | |
|  | 1. **RESEARCH IS RIGOROUS IN CONDUCT**   (Providing a systematic and transparent account of the research process)  Appraisal indicators:  Consider whether | | | | | | | | | | | | | | |  | |  |  | |
|  | 1. *researchers provide a clear account/description of the process by which data was collected (**e.g. for interview method, is there an indication of how interviews were* *conducted?/procedures for collection or recording of data?)* | | | | | | | | | | | | | | |  | |  |  | |
|  | 1. *researchers demonstrate that data collection targeted depth, detail and richness of information (**e.g. interview/observation schedule)* | | | | | | | | | | | | | | |  | |  |  | |
|  | 1. *there is evidence of how descriptive analytical categories, classes, labels, etc. have been generated and used* | | | | | | | | | | | | | | |  | |  |  | |
|  | 1. *presentation of data distinguishes clearly between the data, the analytical frame used, and the interpretation* | | | | | | | | | | | | | | |  | |  |  | |
|  | 1. *methods were modified during the study; and if so, has the researcher explained how and why?* | | | | | | | | | | | | | | |  | |  |  | |
|  | **Rigorous conduct** | **Considerate conduct** | | | | | | | **Critical conduct** | | **Flawed conduct** | | | | | *Worth to continue:* | | | | |
|  |  | | | | | | | | | | | | | | | | | | | |
|  | 1. **RESEARCH FINDINGS ARE CREDIBLE IN CLAIM/BASED ON DATA**   (Providing well-founded and plausible arguments based on the evidence generated)  Appraisal indicators:  Consider whether | | | | | | | | | | | | | | |  | |  |  | |
|  | 1. *there is a clear description of the form of the original data* | | | | | | | | | | | | | | |  | |  |  | |
|  | 1. *sufficient amount of data* *is presented to support interpretations and findings/conclusions* | | | | | | | | | | | | | | |  | |  |  | |
|  | 1. *the researchers explain how the data presented were selected from the original sample to feed into the analysis process (**i.e. commentary and cited data relate; there is an analytical context to cited data, not simply repeated description; is there an account of frequency of presented data?)* | | | | | | | | | | | | | | |  | |  |  | |
|  | 1. *there is a clear and transparent link between data, interpretation, and findings/conclusion* | | | | | | | | | | | | | | |  | |  |  | |
|  | 1. *there is evidence (of attempts) to give attention to negative cases/outliers etc.* | | | | | | | | | | | | | | |  | |  |  | |
|  | **Credible claims** | **Arguable claims** | | | | | **Doubtful claims** | | | | **Not credible** | | | | | *If findings not credible, can data still be used?* | | | | |
|  |  | | | | | | | | | | | | | | | | | | | |
|  | 1. **REASEARCH ATTENDS TO CONTEXTS**   (Describing the contexts and particulars of the study)  Appraisal indicators:  Consider whether | | | | | | | | | | | | | | |  | |  |  | |
|  | 1. *there is an adequate description of the contexts of data sources and how they are retained and portrayed?* | | | | | | | | | | | | | | |  | |  |  | |
|  | 1. *participants’ perspectives/observations are placed in personal contexts* | | | | | | | | | | | | | | |  | |  |  | |
|  | 1. *appropriate consideration is given to how findings relate to the contexts (how findings are influenced by or influence the context)* | | | | | | | | | | | | | | |  | |  |  | |
|  | 1. *the study makes any claims (implicit or explicit) that infer generalisation (if yes, comment on appropriateness)* | | | | | | | | | | | | | | |  | |  |  | |
|  | **Context central** | **Context considered** | | | | | | **Context mentioned** | | | | | **No context attention** | | |  | | | | |
|  |  | | | | | | | | | | | | | | | | | | | |
|  | 1. **RESEARCH IS REFLECTIVE**   (Assessing what factors might have shaped the form and output of research)  Appraisal indicators:  Consider whether | | | | | | | | | | | | | | |  | |  |  | |
|  | 1. *appropriate consideration is given to how findings relate to researchers’ influence/own role during analysis and selection of data for presentation* | | | | | | | | | | | | | | |  | |  |  | |
|  | 1. *researchers have attempted to validate the credibility of findings (**e.g. triangulation, respondent validation, more than one analyst)* | | | | | | | | | | | | | | |  | |  |  | |
|  | 1. *researchers explain their reaction to critical events that occurred during the study* | | | | | | | | | | | | | | |  | |  |  | |
|  | 1. *researchers discuss ideological perspectives/values/philosophies and their impact on the methodological or other substantive content of the research (implicit/explicit)* | | | | | | | | | | | | | | |  | |  |  | |
|  | **Reflection** | **Consideration** | | | | **Acknowledgement** | | | | | | **Unreflective research** | | | | | *NB: Can override previous exclusion!* | | | |
| OVERALL CRITICAL APPRAISAL DECISION  Decision rule:  - a single critical appraisal judgement^^[[4]](#footnote-5)^^ in any of the 6 appraisal domains leads to a critical overall judgement.  - 2 or more high critical appraisal judgements in any of the 6 appraisal domains lead to an overall high risk of bias / low quality rating.  - 2 or more moderate critical appraisal judgements in any of the 6 appraisal domains lead to an overall moderate risk of bias / moderate quality rating.  - which means that for a study to be rated of low risk of bias / high quality at least 5 appraisal domains need be rated as of low critical appraisal. | | | | | | | | | | | | | | | | | | | | |
| HIGH QUALITY  EMPIRICAL RESEARCH  (Study generates new evidence relevant to the review question and complies with all methodological criteria to ensure reliability and empirical grounding of the evidence). | **MODERATE QUALITY**  **EMPIRICAL RESEARCH**  (Study generates new evidence relevant to the review question and complies with reasonable methodological criteria to ensure reliability and empirical grounding of the evidence). | | | | | | | | | **LOW QUALITY**  **EMPIRICAL RESEARCH**  (Study generates new evidence relevant to the review question and complies with minimum methodological criteria to ensure reliability and empirical grounding of the evidence). | | | | | | | | **CRITICAL QUALITY**  **EMPIRICAL RESEARCH**  (The evidence generated by the study does not comply with minimum methodological criteria to ensure reliability and empirical grounding of the evidence). | | |
|  | | | | | | | | | | | | | | | | | | | | |
|  | | | | | | | | | | | | | | | | | | | | |
|  | | | | | | | | | | | | | | | | | | | | |
| Study type | | | | **Methodological appraisal criteria** | | | | | | | | | | | | | | **Response** | | |
|  |  |  |  |  |  |  |  |  |  |  |  |  |  |  |  |  |  | Yes | No | Comment /confidence judgment |
| *2. Mixed-methods^2^*  *Sequential explanatory design*  *The quantitative component is followed by the qualitative. The purpose is to explain quantitative results using qualitative findings. E.g., the quantitative results guide the selection of qualitative data sources and data collection, and the qualitative findings contribute to the interpretation of quantitative results.*  *Sequential exploratory design the qualitative component is followed by the quantitative. The purpose is to explore, develop and test an instrument (or taxonomy), or a conceptual framework (or theoretical model). E.g., the qualitative findings inform the quantitative data collection, and the quantitative results allow a generalization of the qualitative findings.*  *Triangulation designs the qualitative and quantitative components are concomitant. The purpose is to examine the same phenomenon by interpreting qualitative and quantitative results (bringing data analysis together at the interpretation stage), or by integrating qualitative and quantitative datasets (e.g., data on same cases), or by transforming data (e.g., quantization of qualitative data).*  *Embedded/convergent design The qualitative and quantitative components are concomitant. The purpose is to support a qualitative study with a quantitative sub-study (measures), or to better understand a specific issue of a quantitative study using a qualitative sub-study, e.g., the efficacy or the implementation of an intervention based on the views of participants.* | | | | 1. **RESEARCH INTEGRATION/SYNTHESIS OF METHODS**   (Assessing the value-added of the mixed-methods approach)  Applied mixed-methods design:   - Sequential explanatory design - Sequential explorative design - Triangulation design - Embedded design   Appraisal indicators:  Consider whether | | | | | | | | | | | | | |  |  |  |
|  |  |  |  | 1. *the rationale for integrating qualitative and quantitative methods to answer the research question is explained*   *[DEFENSIBLE]* | | | | | | | | | | | | | |  |  |  |
|  |  |  |  | 1. *the mixed-methods research design is relevant to address the qualitative and quantitative research questions, or the qualitative and quantitative aspects of the mixed methods research question*   *[DEFENSIBLE]* | | | | | | | | | | | | | |  |  |  |
|  |  |  |  | 1. *there is evidence that data gathered by both research methods was brought together to inform new findings to answer the mixed-methods research question (**e.g. form a complete picture, synthesise findings, configuration)*   *[CREDIBLE]* | | | | | | | | | | | | | |  |  |  |
|  |  |  |  | 1. *the approach to data integration is transparent and rigorous in considering all findings from both the qualitative and quantitative module (danger of cherry-picking)*   *[RIGOROUS]* | | | | | | | | | | | | | |  |  |  |
|  |  |  |  | 1. *appropriate consideration is given to the limitations associated with this integration, e.g., the divergence of qualitative and quantitative data (or results)?*   *[REFLEXIVE]* | | | | | | | | | | | | | |  |  |  |
| For mixed-methods research studies, each component undergoes its individual critical appraisal first. Since qualitative studies are either included or excluded, no combined risk of bias assessment is facilitated, and the assigned risk of bias from the quantitative component similarly holds for the mixed-methods research.  The above appraisal indicators only refer to the applied mixed-methods design. If this design is not found to comply with each of the four mixed-methods appraisal criteria below, then the quantitative/qualitative components will individually be included in the review: | | | | | | | | | | | | | | | | | | | | |
| Mixed-methods critical appraisal:  Research is defensible in design  Research is rigorous in conduct  Research is credible in claim  Research is reflective | | | | Qualitative critical appraisal:  Include / Exclude | | | | | | | | | | | | Quantitative critical appraisal:   1. Low risk of bias 2. Risk of bias 3. High risk of bias 4. Critical risk of bias | | | | |
| Combined appraisal:  Include / Exclude mixed-methods findings judged with ____________________________ risk of bias | | | | | | | | | | | | | | | | | | | | |
|  | | | | | | | | | | | | | | | | | | | | |
|  | | | | | | | | | | | | | | | | | | | | |

# Appendix F: Characteristics of included studies

## F.1: Overview of included studies

| **Study** | **Programme** | **Country** | **Description** | **Method** |
| --- | --- | --- | --- | --- |
| Aevarsdottir et al. (2017) | Tanzania GiveWatts Solar Lamps | Tanzania | GiveWatts offered households of school children an opportunity to acquire solar lamps with mobile phone chargers at subsidised prices. In total, 60 schools benefited from subsidies while 9 acted as the control group. | RCT |
| Aklin et al. (2017) | India Mera Gao Power (MGP) Solar Microgrid | India | Solar microgrids were provided to non-grid communities in Utter Pradesh, India, conditional on 10 or more households agreeing to pay a monthly subscription of 100 rupees. | RCT |
| Araujo-Bonjean et al. (2015) | Programme de Plateform Multifonctionnelles (PTFM) | Mali | The treatment group were provided with an engine that was capable of running on both diesel and jatropha oil, a renewable alternative. The engine was used to power either parts or the entirety of villages. | RCT |
| Arráiz and Calero (2014) | ACCIONA Microenergía Peru (AMP) | Peru | AMP was launched in 2009 and provided access to subsidised solar home systems (SHSs) to clients who entered into and signed contracts with the programme for 20 years. Beneficiary clients were required to pay a monthly service fee, which covers the equipment's rent and maintenance for 20 years. | Statistical matching |
| Bahaj et al. (2019) | Kenya Electric Mini-grids | Kenya | Implemented by the Government of Kenya under the Ministry of Energy, the programme involved the installation of a solar PV mini-grid in a largely non-electrified local trading centre in rural Kenya. | DiD |
| Ballon et al. (2019) | Electrificación Rural con Energía Renovable (PERER) | Bolivia | In this study, the Government of Bolivia directly provided solar panels to beneficiary households. The households were also trained on how to use and maintain the received solar system. The package included a solar panel with a rechargeable battery. | RCT |
| Banerjee et al. (2011) | Rural Energy Development Program (REDP) | Nepal | The REDP programme was implemented by the Nepalese Government in a bid to electrify households. Micro-hydro systems were installed in several locations to provide electricity access to those not connected to the national grid. | Statistical matching |
| Belatramo and Levine (2013) | Solar Household Energy (SHE), Solar Ovens | Senegal | SHE distributed solar ovens to beneficiary households which heavily relied on firewood for cooking. SHE trained staff and demonstrated how the oven was used. Staff then passed on the training to village women leaders who would then support intervention dissemination in the 20 treatment villages. | RCT |
| Bensch et al. (2012) | Electrification Rurale Pour le Sénégal (ERSEN) | Senegal | The ERSEN programme provided solar home systems to households, covering over 100 villages. For villages to be considered for the programme, they had to: 1) be far from the national grid; 2) have social infrastructure facilities (schools and health stations); 3) have a <20% electrification rate. | Statistical matching |
| Bensch et al. (2015) | Yeelen Ba | Burkino Faso | Funded by the Netherlands Ministry of Foreign Affairs, the Yeelen Ba programme began in 2009. A variety of solar home systems were offered using a fee-for-service system which allows for solar panel maintenance by local businessmen hence paving the way for self-sustainability. | Statistical matching |
| Bensch et al. (2021) | EnDev-K | Kenya | The EnDev-K programme worked by mobilising entrepreneurs who had an interest in cookstoves or solar products and offered them enhanced training including installation of products, maintenance as well as marketing/business techniques. Following training, the individuals were then linked to companies dealing in local distribution of the said products. | Statistical matching |
| Bharadwaj et al. (2022) | Renewable Energy Subsidy Policy (RESP) | Nepal | Implemented by the Government of Nepal, RESP was aimed at providing solar home systems to households at a subsidised cost to increase affordability and uptake of the technology thereby improving rates of energy access in poor communities such as the Hindu Kush Himalaya. | DiD |
| Bonan et al. (2023) | Pakistan Solar Home Systems (SHS) Payment Experiment | Pakistan | This experiment involved providing solar home system beneficiaries with flexible payment plans. The experiment was conducted to stimulate demand for solar home systems as well as to encourage beneficiary families to adopt better payment behaviour for the products. | RCT |
| Burgess et al. (2023) | India Husk Power Systems (HPS) Solar Microgrid | India | Implemented by Husk Power Systems (HPS), the solar microgrid is the main power source in off-grid villages in Bihar, India. The experiments in the study involved varying prices of the solar microgrids, thereby determining demand for the technology. | RCT |
| Burney et al. (2017) | Solar Electric Light Fund (SELF) Solar Market Garden Programme | Benin | The solar market garden (SMG) was targeted towards farming groups made up of local women to empower them through diversifying and increasing agricultural productivity. SMGs were installed on land that belonged to women agricultural groups and that was formally registered with a land title. Each installed SMG was intended to serve 25 to 40 members. | DiD |
| Chen et al. (2017) | d.Light’s D20g Solar Home System (SHS) | Uganda | d.Light offered SHS to households in rural eastern Uganda using a flexible payment mechanism whereby clients would make payments over a period of 12 months, before gaining full ownership. In addition to the flexible payment mechanism, implementers allowed clients to make payments via mobile money. | Statistical matching |
| Clarke et al. (2020) | Solar Light Discount Scheme | Rwanda | In this programme, solar lighting was directly provided to households free of charge, however, beneficiaries were required to pay recharging costs for a subsidised fee. | RCT |
| Dherani et al. (2022) | Malawi Streptococcus Pneumoniae Carriage and Air Pollution Exposure (MSCAPE) | Malawi | MSCAPE involved the direct distribution of solar-improved cookstoves to replace three-stone open fires, the most common cooking mechanism in rural Malawi. | RCT |
| Grimm et al. (2016) | Rwanda Subsidised Solar Kits | Rwanda | In this experiment, researchers elicited willingness to pay for solar products including the d.light S2, Sun King Pro 2 and ASE 20W Solar DC Lighting Kit. Participants were ultimately able to purchase these technologies at a subsidised price. | RCT |
| Grimm et al. (2017) | Rwanda ToughStuff Pico-Photovoltaic (Pico-PV) Kits | Rwanda | ToughStuff distributed Pico-PV kits to rural Rwandan households. The distributed kits supported lighting, charging mobile phones and had a radio device. | RCT |
| IDinsight (n.d.) | D20g Go-to-Market Pilot | Uganda | d.Light D20g solar home systems were brought to market in rural Uganda. The D20g system's solar panel supported at least four LED light bulbs, had one portable lantern, supported mobile phone charging and contained an in-built radio. Clients paid for the system using a flexible mobile money payment plan. | DiD |
| Karumba et al. (2017) | Government of Kenya Micro-hydro Scheme | Kenya | The Government of Kenya supported the setup of micro-hydro systems which serve communities’ energy needs. Interested communities were required to partake in the setup and installation of the systems. | Statistical matching |
| Kelly (2018) | Cooking and Pneumonia Study (CAPS) | Malawi | CAPS involved the direct provision of solar-improved cookstoves to rural households along with training on usage and maintenance. | RCT |
| Khandker et al. (2014) | Rural Electrification and Renewable Energy Development Project (RERED II) | Bangladesh | Implemented by the Infrastructure Development Company Limited (IDCOL), RERED II involved installing solar home systems on credit as well as offering training support to its clients, partnering with organisations and their field workers. Topics covered during training included how to install the system, system maintenance and community awareness/marketing. | Statistical matching |
| Koima et al. (2024) | Kenyan Government Solar Photovoltaics | Kenya | This programme was an initiative by the Government of Kenya to provide electricity access to government primary schools not connected to the national grid. In these schools, solar PV systems were installed to support energy needs. | DiD |
| Kudo et al. (2019) | Bangladesh Gana Unnayan Kendra (GUK) Free d.light Solar Products | Bangladesh | In this programme, the NGO Gana Unnayan Kendra (GUK) directly provided d-light solar products to beneficiaries. Beneficiaries were fourth to eight-grade students in northern Bangladesh where kerosene lamps were commonly used. | RCT |
| Lang (2020) | Pay As You Go (PAYGo) Solar Credit | Rwanda | In this experiment, PAYGo solar clients were randomly offered a line of credit which covered the access time cost of their solar technologies. | RCT |
| Langbeen et al. (2022) | Rural Renewable Energy Project (RREP) | Sierra Leone | Implemented by the Government of Sierra Leone in partnership with the United Nations Office for Project Services (UNOPS), the Rural Renewable Energy Project (RREP) aimed to improve off-grid electricity access by installing mini-grids across communities and health centres. | DiD |
| Li et al. (2023) | Photovoltaic Poverty Alleviation Projects (PVPA) | China | Implemented by the Government of China, the PVAP programme involved the installation of community-level PV stations to serve a communities’ energy needs. The programme also allowed for communities to gain financially from the implementation of stations, expenditure which was primarily spent on community improvements. | DiD |
| Lu (2020) | India Mera Gao Power (MGP) Microgrid | India | Mera Gao Power (MGP) installed microgrids in rural communities in the Barabanki district of India. Systems were installed in areas where at least ten households came together to pay 100 rupees to the implementation agency per month. | RCT |
| Lucchino (2017) | Kenya Student Lamps | Kenya | In this experiment, pupils of primary schools in two Kenyan districts, Loitokitok and Nzaui were provided with solar lamps free of charge. Distribution of the solar lamps took place at the start of the school term. | RCT |
| Mahajan et al. (2020) | India Solar Village Project (SVP) Free Lanterns | India | Implemented by the Solar Village Project (SVP), solar lanterns were directly provided free of charge to households without grid electricity access in Bahraich district, India. The distributed lanterns could also support mobile charging. | RCT |
| Mekonnen et al. (2021) | Ethiopia Sun-king Pico Lantern | Ethiopia | This experiment elicited willingness to pay for solar lanterns and involved an information mechanism, a financial mechanism and a combination of the two. Ultimately, participants were able to purchase the lanterns. | RCT |
| Meriggi et al. (2021) | Cameroon d.light S20 Subsidies | Cameroon | Subsidies of varying prices were provided to programme participants to elicit willingness to pay for the d.light S20 product. Ultimately, participants were able to purchase the products. | RCT |
| Michler et al. (2021) | Zambia Solar Cook Stoves | Zambia | In this programme, solar stoves were distributed to participants, with the intervention targeting those who were part of nutritional cooking clubs and farm demonstration plots. | RCT |
| Nightingale et al. (2019) | Cooking and Pneumonia Study (CAPS) | Malawi | CAPS involved the direct provision of solar-improved cookstoves to rural households along with training on usage and maintenance | RCT |
| Numminen et al. (2018) | Solar Microgrid Dynamic Pricing Experiment | India | This programme involved the installation of solar microgrids in Unnao district, India. Further, in the treatment group (dynamic pricing) the battery status determined the price of their solar products determined the price. If the battery voltage was low or above a specified threshold, the control station issued a signal to the household meter to vary the electricity price. | RCT |
| Nyakato et al. (2018) | Uganda Smoke-Free Solar Lights | Uganda | Over a 3 months study period, the respiratory health impact of smoke-free solar lights was assessed using a sample of 230 people in rural Uganda. Following the distribution of the solar lanterns to participants, they were encouraged to replace their kerosene lamps with the solar lanterns. | RCT |
| Rom et al. (2023) | Western Kenya Primary Schools SolarAid Portable Solar Lights | Kenya | Implemented by SolarAid, this RCT was implemented in primary schools and assigned students to multiple treatments arms were the price and type of solar light varied. Treatments were either a basic light (given free of charge), a large light (also given free of charge), or subsidies for a light at three different levels (low, medium or high). | RCT |
| Stojanovski et al. (2021) | Zambia Government Primary School Solar Lantern Gifts | Zambia | In this study, grade 7 - 9 students in government primary schools in Zimba district, Zambia were gifted a solar lantern in 2016 at the beginning of their second school term. The students chosen to benefit were not connected to the national grid. | RCT |
| Thompson (2017) | Nepalese Government Micro-hydro Plant | Nepal | The government of Nepal installed micro-hydro plants as part of their plan to increase electricity access across the country. This study utilises this large scale programme to look at the socio-economic impacts of increased energy access. | IVs |
| UNICEF (2022) | Mwangaza Mashinani Pilot Project | Kenya | The Mwangaza Mashinani pilot programme aimed at improving health, education and livelihoods of vulnerable households in Garissa and Kilifi with emphasis on women and children through enhanced clean energy access achieved through high subsidy support to purchase solar products. | Statistical matching |
| Urpelainen et al. (2017) | India Boond Solar Home System Demonstrations | India | This programme solely focused on information and marketing dissemination. The implementer, Boond, demonstrated within villages how the solar technology worked, what financial support was available to households to help them acquire the product, which payment mechanisms were accepted and responded to questions during demonstrations. | RCT |
| Wagner et al. (2021) | Kenya Solar Home Systems | Kenya | Funded by the Dutch Entrepreneurial Development Bank (FMO), the programme offered credit for SHS, stimulating demand for the technologies. The credit system was made possible by partnering with local manufacturers, solar energy systems distributors as well as microfinance institutions which ran credit groups. | RDD |
| Wallach et al. (2022) | Uganda Allmar Solar Systems Solar Lamp | Uganda | Solar lamps were directly provided, free of charge, to 80 women who mostly relied on traditional fuels such as kerosene and candles for lighting with few using off-grid or grid lighting. Researchers worked under a hypothesis that the introduction of solar lamps would eventually diminish the use of traditional fuels hence impacting the quality of air in the target households. | RCT |
| Wang et al. (2011) | Bangladesh Infrastructure Development Company Limited (IDCOL) SHS Programmes | Bangladesh | Solar home systems in Bangladesh were provided to beneficiary households using two purchase models. In the first model, the system is installed in the household, who are then required to pay a fixed amount each month. In the second, the implementer, Infrastructure Development Company Limited (IDCOL), partnered with microfinance organisations to loan the systems to households. | Statistical matching |
| Xiao et al. (2023) | Photovoltaic Poverty Alleviation Projects (PVPA) | China | Implemented by the Government of China, the PVAP programme involved the installation of community-level PV stations to serve a communities’ energy needs. The programme also allowed for communities to gain financially from the implementation of stations, expenditure which was primarily spent on community improvements. | RDD |

## F.2: Moderator characteristics of included studies

| **Study** | **Technology** | **Mechanism** | **Scale** | **Supply/demand** | **Target actor** | **Target population** | **Implementation agency** |
| --- | --- | --- | --- | --- | --- | --- | --- |
| Aevarsdottir et al. (2017) | Solar lamp/ lantern with mobile phone charger | Financial (credit or subsidy) | Local Policy | Demand | Households | None | Non-profit organization |
| Aklin et al. (2017) | Solar mini-grid or microgrid | Opportunity to access/market expansion | Local Policy | Demand | Communities | None | For-profit firm |
| Araujo-Bonjean et al. (2015) | Engine | Direct provision | National Policy | Demand | Communities | None | Government agency |
| Arráiz and Calero (2014) | Solar home system (SHS) | Financial (credit or subsidy) | Regional Policy | Demand | Communities | None | Government agency |
| Bahaj et al. (2019) | Solar mini-grid or microgrid | Direct provision | Local Policy | Demand | Communities | None | Government agency |
| Ballon et al. (2019) | Solar panels (not part of a wider system) | Multi-mechanism | Regional Policy | Demand | Households | None | Government agency |
| Banerjee et al. (2021) | Micro-hydro | Direct provision | National Policy | Demand | Households | None | Government agency |
| Belatramo and Levine (2013) | Solar oven | Direct provision | Regional Policy | Demand | Individuals | Women | Non-profit organization |
| Bensch et al. (2012) | Solar home system (SHS) | Direct provision | National Policy | Demand | Schools | None | Government agency |
| Bensch et al. (2015) | Solar home system (SHS) | Financial (credit or subsidy) | Regional Policy | Both | Households & Communities | None | Non-profit organization |
| Bensch et al. (2021) | General solar products | Information | National Policy | Supply | Individual traders | None | Government agency |
| Bharadwaj et al. (2022) | Solar home system (SHS) | Financial (credit or subsidy) | National Policy | Supply | Communities | None | Government agency |
| Bonan et al. (2023) | Solar home system (SHS) | Multi-mechanism | Regional Policy | Both | Households | None | For-profit firm |
| Burgess et al. (2023) | Solar mini-grid or microgrid | Financial (credit or subsidy) & Opportunity to access/market expansion | Regional Policy | Demand | Households | None | For-profit firm |
| Burney et al. (2017) | Solar market garden (SMG) | Direct provision | Local Policy | Demand | Households | Women | Non-profit organization |
| Chen et al. (2017) | Solar home system (SHS) | Financial (credit or subsidy) | Regional Policy | Demand | Households | None | For-profit firm |
| Clarke et al. (2020) | Solar lamp/ lantern | Financial (credit or subsidy) & Multi-mechanism | Regional Policy | Demand | Households | None | Non-profit organization |
| Dherani et al. (2022) | Solar-improved cookstove (ICS) | Direct provision | Local Policy | Demand | Households | None | Not Specific |
| Grimm et al. (2016) | Solar lamp/ lantern; Solar home system (SHS) | Financial (credit or subsidy) & Direct provision | National Policy | Demand | Households & Communities | None | For-profit firm |
| Grimm et al. (2017) | Solar lamp/ lantern with mobile phone charger | Direct provision | National Policy | Demand | Households | None | For-profit firm |
| IDinsight (n.d.) | Solar home system (SHS) | Opportunity to access/market expansion | Regional Policy | Demand | Households | None | For-profit firm |
| Karumba et al. (2017) | Micro-hydro | Opportunity to access/market expansion | National Policy | Demand | Households | None | Government agency |
| Kelly (2018) | Solar-improved cookstove (ICS) | Direct provision | Local Policy | Demand | Households | None | Not Specific |
| Khandker et al. (2014) | Solar home system (SHS) | Multi-mechanism | National Policy | Both | Households | None | Government agency |
| Koima et al. (2024) | Solar mini-grid or microgrid | Direct provision | National Policy | Demand | Schools | None | Government agency |
| Kudo et al. (2019) | Solar lamp/ lantern; Solar lamp/ lantern with mobile phone charger | Direct provision | Regional Policy | Demand | Individuals | Students | Non-profit organization |
| Lang (2020) | Solar home system (SHS) | Financial (credit or subsidy) | National Policy | Demand | Households | None | For-profit firm |
| Langbeen et al. (2022) | Solar mini-grid or microgrid | Direct provision | National Policy | Demand | Households | None | International aid agency |
| Li et al. (2023) | Solar mini-grid or microgrid | Financial (credit or subsidy) | National Policy | Demand | Communities | None | Government agency |
| Lu (2020) | Solar mini-grid or microgrid | Opportunity to access/market expansion | Local Policy | Demand | Households | None | For-profit firm |
| Lucchino (2017) | Solar lamp/ lantern | Direct provision | Regional Policy | Demand | Individuals | Students | Non-profit organization |
| Mahajan et al. (2020) | Solar lamp/ lantern with mobile phone charger | Direct provision | Regional Policy | Demand | Households | None | Not Specific |
| Mekonnen et al. (2021) | Solar lamp/ lantern | Financial (credit or subsidy), Information & Multi-mechanism | National Policy | Demand | Households | None | Not Specific |
| Meriggi et al. (2021) | Solar lamp/ lantern | Financial (credit or subsidy) | Regional Policy | Demand | Households | None | For-profit firm |
| Michlera et al. (2021) | Solar-improved cookstove (ICS) | Direct provision | Regional Policy | Demand | Households | None | Not Specific |
| Nightingale et al. (2019) | Solar-improved cookstove (ICS) | Direct provision | Local Policy | Demand | Households | None | Not Specific |
| Numminen et al. (2018) | Solar mini-grid or microgrid | Financial (credit or subsidy) | Local Policy | Demand | Households | None | For-profit firm |
| Nyakato et al. (2018) | Solar lamp/ lantern | Direct provision | Local Policy | Demand | Households | None | Not Specific |
| Oxford Policy Management (2022) | Solar lamp/ lantern | Financial (credit or subsidy) | Regional Policy | Demand | Households | None | Government agency |
| Rom et al. (2023) | Solar lamp/ lantern; Solar lamp/ lantern with mobile phone charger | Direct provision, Financial (credit or subsidy), Information & Multi-mechanism | Regional Policy | Demand | Households | Students | Charitable or private foundation |
| Stojanovski et al. (2021) | Solar lamp/ lantern | Direct provision | Local Policy | Demand | Individuals | Students | Charitable or private foundation |
| Thompson (2017) | Micro-hydro | Direct provision | National Policy | Demand | Households | None | Government agency |
| Urpelainen et al. (2017) | Solar home system (SHS) | Information | Regional Policy | Demand | Households | None | For-profit firm |
| Wagner et al. (2021) | Solar home system (SHS) | Financial (credit or subsidy) | National Policy | Demand | Households | None | For-profit firm |
| Wallach et al. (2022) | Solar lamp/ lantern | Direct provision | Local Policy | Demand | Individuals | Women | Not Specific |
| Wang et al. (2011) | Solar home system (SHS) | Multi-mechanism | National Policy | Demand | Households | None | Government agency, For-profit firm |
| Xiao et al. (2023) | Solar mini-grid or microgrid | Financial (credit or subsidy) | National Policy | Demand | Communities | None | Government agency |

# Appendix G: Examples of excluded studies

| **Reason for exclusion** | **Reference** | **Overview of the study** | **Explanation for exclusion** |
| --- | --- | --- | --- |
| Intervention | Luan & Lin (2022) | This study evaluates the impact of government subsidies provided to solar photovoltaic businesses in China. They assess the impact on the operating performance of the businesses. | The subsidy provided in this instance is not aimed at increasing the uptake or the use of off-grid technologies, as per the objective of our review. Instead, the aim is to increase the performance of businesses generating solar photovoltaic technologies and therefore is outside of our review’s scope. |
| Outcome | Kishore et al. (2017) | The study evaluates a programme where solar panels were used to revive non-functioning tubewells in Bihar, India. The evaluation focuses on the productive impact of access to solar irrigation. | While the intervention is of relevance to our review, this being solar-powered irrigation, the study does not focus on an included outcome. The study only reports productivity outcomes related to agricultural yields such as rice and wheat and therefore is outside of our review’s scope. |
| Outcome | Xu et al. (2022) | This study evaluates the impact of the Photovoltaic Poverty Alleviation (PVPA) programme in China and its effect on the economic development of Chinese counties. | While the programme is of relevance to our review, and we have included two studies evaluating this programme, the only outcome measured is the GDP of Chinese counties. While we include *income* as an economic outcome, county-level GDP does not fit within the scope of our review. |
| Outcome | Gillani et al. (2022) | This study evaluates the impact of installing solar panels in schools in Pakistan. The authors evaluate whether the installation of solar panels affects school enrollment. | The intervention within this study is of relevance to our review and an education outcome is measured. However, the measure used within this paper is school enrollment, whether a child is enrolled in school. As this is not a measure of *school attendance*, whether the child ever attends school, it falls outside the scope of our included education outcomes. |
| Technology | Nagwekar et al. (2020) | This study evaluates the impact of the provision of solar dryers in India. Specifically, how the dryers can improve nutrition and food security outcomes as food can be stored for longer. | The technology included in this study was excluded from our review. While the solar dryer may have impacts on nutrition and food security, the technology is limited in how it can be used by households, as such it falls outside the scope of our review. |

# Appendix H: Risk of bias and critical appraisal results

## H.1: Risk of bias assessment of experimental impact evaluations

| **Study** | **Assignment mechanism** | **Unit of analysis** | **Selection bias** | **Confounding** | **Deviations from intended interventions** | **Performance bias** | **Outcome measurement bias** | **Reporting bias** | **Overall bias** |
| --- | --- | --- | --- | --- | --- | --- | --- | --- | --- |
| Aevarsdottir (2017) | Low | Not applicable | Low | Low | High | Low | Low | Low | **HIGH** |
| Aklin (2017) | Low | Low | Low | Low | Low | Low | Low | Low | **LOW** |
| Ballon (2019) | Low | Low | Low | Low | Low | High | Low | Low | **HIGH** |
| Belatramo (2013) | Low | Not applicable | High | Low | Low | Low | High | Low | **HIGH** |
| Bonan (2023) | Low | Not applicable | High | Low | Low | Low | Low | Low | **HIGH** |
| Bonjean (2015) | Low | Low | Low | Low | Low | Not clear | Not clear | Not clear | **MED** |
| Burgees (2023) | Low | Low | Not clear | Low | Low | Low | Low | Low | **MED** |
| Clarke (2020) | Not clear | Not applicable | Low | High | High | Low | Low | Low | **HIGH** |
| Dherani (2022) | Not clear | Not clear | Not clear | Not clear | High | Low | Not clear | Not clear | **HIGH** |
| Grimm (2013) | Low | Not applicable | Low | Low | Low | Low | Low | High | **HIGH** |
| Grimm (2016) | Low | Low | Low | Low | High | Low | Low | High | **HIGH** |
| Grimm (2017) | Low | Not applicable | Low | Low | Low | High | Low | Low | **HIGH** |
| Hassan (2014) | Low | Not applicable | Not clear | Low | Low | High | Low | High | **HIGH** |
| Kelly (2018) | Low | Low | Not clear | Low | Low | Low | Low | Low | **MED** |
| Kudo (2019a) | Low | Not applicable | Low | Low | Low | Low | Low | Low | **LOW** |
| Kudo (2015) | Low | Not applicable | Low | Low | High | High | Low | Low | **HIGH** |
| Kudo (2017) | Low | Not applicable | Low | Low | Low | High | Low | Low | **HIGH** |
| Kudo (2019b) | Low | Not applicable | Low | Low | High | High | Not clear | High | **HIGH** |
| Lang (2020) | Low | Not applicable | High | Low | Low | Low | Low | High | **HIGH** |
| Lu (2020) | Low | Not clear | Low | Low | Low | Low | Low | High | **HIGH** |
| Lucchino (2017a) | Low | Not applicable | Low | Low | Low | High | Low | Low | **HIGH** |
| Lucchino (2017b) | Not clear | Not applicable | Not clear | Low | Not clear | High | Low | Low | **HIGH** |
| Mahajan (2020) | Low | Not applicable | Low | Low | Low | Low | Low | Low | **LOW** |
| Mekonnen (2021) | Low | Not applicable | Low | Low | Low | Low | Low | Low | **LOW** |
| Meriggi (2021) | Low | Low | Low | Low | Low | Low | Low | Low | **LOW** |
| Michler (2021) | Not clear | Not applicable | Not clear | High | High | Low | Low | Low | **HIGH** |
| Nightingale (2019) | Not clear | Not applicable | Low | Not clear | High | Low | Low | Not clear | **HIGH** |
| Numminen (2018) | Low | Low | Low | Low | High | Low | Low | Low | **HIGH** |
| Nyakato (2018) | Not clear | Not clear | Low | Not clear | High | Low | Low | High | **HIGH** |
| Rom (2023) | Low | Not applicable | Low | Low | Not clear | High | Low | Low | **HIGH** |
| Stojanovski (2021) | Low | Not applicable | Low | Low | Low | Low | Low | Not clear | **MED** |
| Urpelainen (2017) | Low | Low | Low | Low | Low | Low | Low | Low | **LOW** |
| Wallach (2022) | Low | Not applicable | Low | Low | Low | Low | Low | High | **HIGH** |

Note: Luchino (2017a) refers to chapter 3 from this publication while Luchino (2017b) refers to chapter 4.

## H.2: Risk of bias assessment of quasi-experimental impact evaluations

| **Study** | **Selection bias** | **Confounding** | **Performance bias** | **Spill-overs, cross-overs and contamination** | **Outcome measurement bias** | **Reporting bias** | **Overall** |
| --- | --- | --- | --- | --- | --- | --- | --- |
| Arraiz (2015) | Low | Low | Low | Low | Not clear | Low | **MED** |
| Bahaj (2019) | High | High | Low | Low | Not clear | Low | **HIGH** |
| Banerjee (2011) | Low | Low | High | Low | Low | Not clear | **HIGH** |
| Bensch (2012) | Low | High | Low | High | Not clear | Not clear | **HIGH** |
| Bensch (2015) | Low | Low | Low | Low | Low | Not clear | **MED** |
| Bensch (2021) | Low | High | Low | Low | Low | Low | **HIGH** |
| Bharadwaj (2022) | Not clear | Not clear | Low | High | Low | Low | **HIGH** |
| Binci (2022) | Low | High | Low | High | Low | Not clear | **HIGH** |
| Bonjean (2015) | Low | High | Not clear | Low | Not clear | Not clear | **HIGH** |
| Burney (2017) | High | High | Low | Not clear | Not clear | Low | **HIGH** |
| Chen (2017) | Low | High | High | High | Low | Not clear | **HIGH** |
| Karumba (2017) | High | Low | Low | Low | High | Low | **HIGH** |
| Khandker (2014) | Not clear | Not clear | Low | Not clear | Not clear | High | **HIGH** |
| Koima (2024) | Low | Low | Low | Low | High | Low | **HIGH** |
| Langbeen (2022) | High | High | Low | Not clear | Not clear | Low | **HIGH** |
| Li (2023) | Low | Low | Low | Low | Low | Low | **LOW** |
| IDInsight (2018) | Low | Not clear | Low | High | Low | High | **HIGH** |
| Thompson (2017) | Low | Low | Low | Not clear | Low | Not clear | **MED** |
| Wagner (2021) | Low | High | Low | Not clear | Not clear | Low | **HIGH** |
| Wang (2011) | Low | High | Low | High | Low | Not clear | **HIGH** |
| Xiao (2023) | Low | Not clear | Low | Not clear | Low | Low | **MED** |

## H.3: Critical appraisal of qualitative studies

| Study | **Defensible design** | **Appropriate sample** | **Rigorous research** | **Credible findings** | **Contextual research** | **Reflective research** | **Overall** |
| --- | --- | --- | --- | --- | --- | --- | --- |
| Khan (2017) | Critical | Flawed |  |  |  |  | **CRITICAL** |
| Eras-Almeida (2019) | Critical | Critical | Considerate | Doubtful | Mentioned | Acknowledged | **LOW** |
| Etienne (2024) | Defensible | Functional | Rigorous | Credible | Centred | Considerate | **MED** |
| Ponticiello (2023) | Defensible | Appropriate | Rigorous | Credible | Centred | Acknowledged | **HIGH** |
| Cundale (2017) | Defensible | Functional | Rigorous | Arguable | Centred | Acknowledged | **MED** |
| Chen (2021) | Defensible | Functional | Rigorous | Credible | Centred | Acknowledged | **MED** |
| Sundararajan (2022) | Defensible | Functional | Rigorous | Credible | Considerate | Considerate | **MED** |
| IOB (2014) | Arguable | Critical | Flawed |  |  |  | **CRITICAL** |
| Levine (2022) | Arguable | Functional | Critical | Arguable | Centred | Acknowledged | **MED** |
| Grimm (2013) | Arguable | Functional | Critical | Doubtful | Centred | Acknowledged | **MED** |
| Urmee (2011) | Arguable | Critical | Critical | Doubtful | Considerate | Acknowledged | **MED** |
| Bensch (2015) | Arguable | Critical | Critical | Doubtful | Centred | Acknowledged | **MED** |
| Wang (2023) | Defensible | Appropriate | Rigorous | Credible | Centred | Reflective | **HIGH** |
| Oxford Policy Management(2022) | Defensible | Appropriate | Rigorous | Credible | Centred | Considerate | **HIGH** |
| Bahaj (2019) | Critical | Critical | Critical | Doubtful | Mentioned | Acknowledged | **LOW** |
| Liao (2021) | Arguable | Critical | Flawed |  |  |  | **CRITICAL** |
| Lo (2021) | Critical | Critical | Critical | Arguable | Considerate | Acknowledged | **LOW** |
| Bhattarai (2018) | Critical | Critical | Critical | Doubtful | Mentioned | Unreflective | **CRITICAL** |
| Bensch (2016) | Arguable | Functional | Critical | Arguable | Considerate | Acknowledged | **MED** |

## H.4: Critical appraisal of mixed-methods studies

| **Study** | **Appropriate rationale and design** | **Complete picture** | **Transparent & rigorous integration** | **Limitations acknowledged** |
| --- | --- | --- | --- | --- |
| Khan (2017) | Yes | Yes | No | No |
| Sundararajan (2022) | Yes | Yes | No | No |
| Levine (2022) | Yes | Yes | No | No |
| Grimm (2013) | Yes | Yes | No | No |
| Urmee (2011) | No | No | No | No |
| Bensch (2015) | Yes | Yes | Yes | No |
| Wang (2023) | Yes | Yes | Yes | Yes |
| Oxford Policy Management(2022) | Yes | Yes | No | No |
| Bahaj (2019) | Yes | Yes | No | No |
| Lo (2021) | No | No | No | No |
| Bensch (2016) | Yes | Yes | No | No |

# Appendix I: Funnel plots

Appendix Figure 1. Funnel plot for hours of lighting use


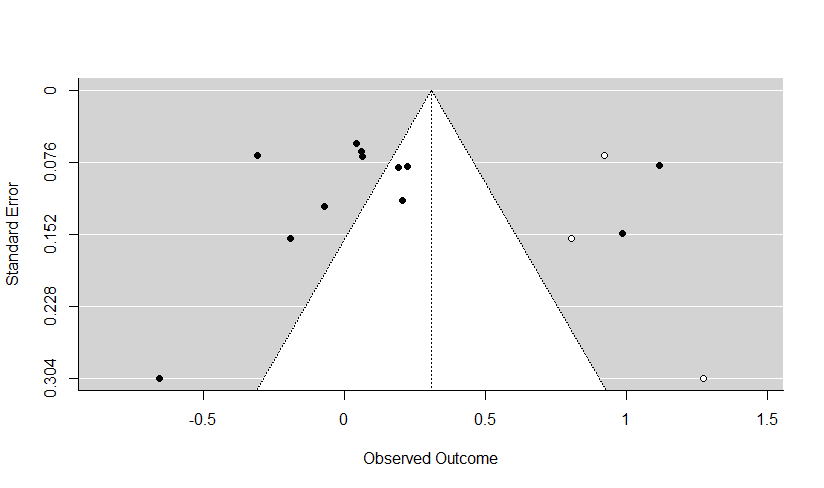


Appendix Figure 2. Funnel plot for energy expenditure (all)


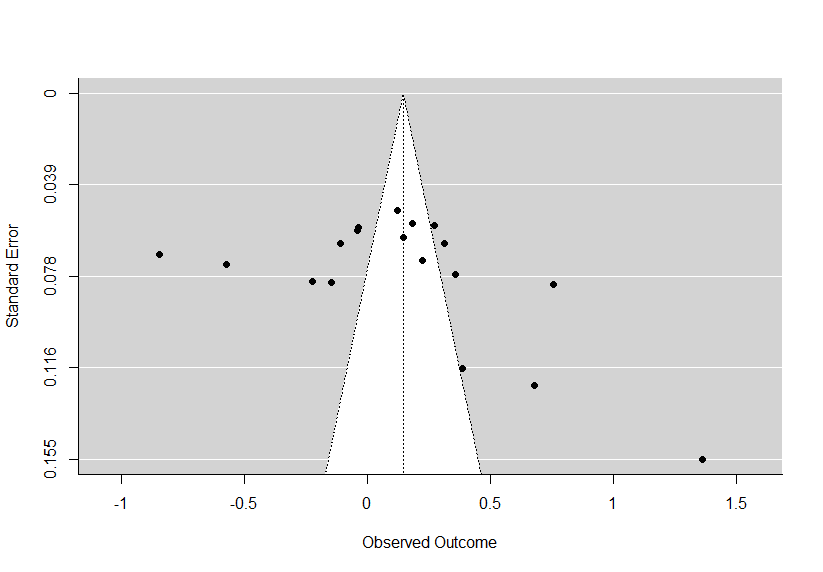


Appendix Figure 3. Funnel plot for household (or personal) income


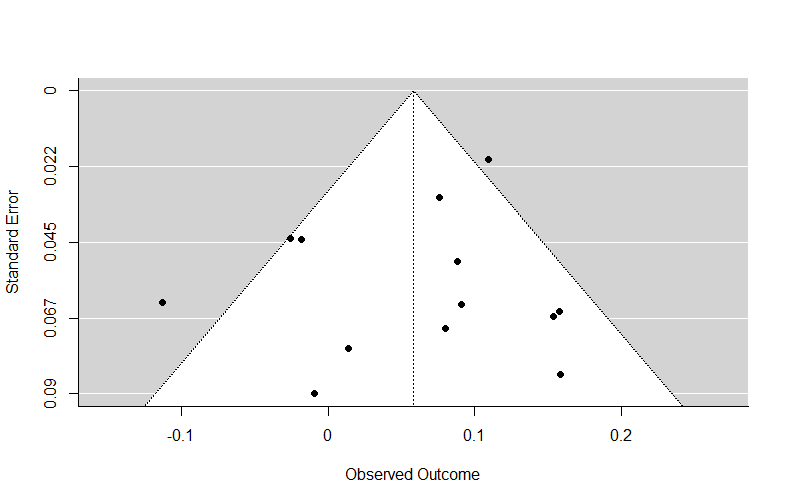


Appendix Figure 4. Funnel plot for time spent working (renumerated)


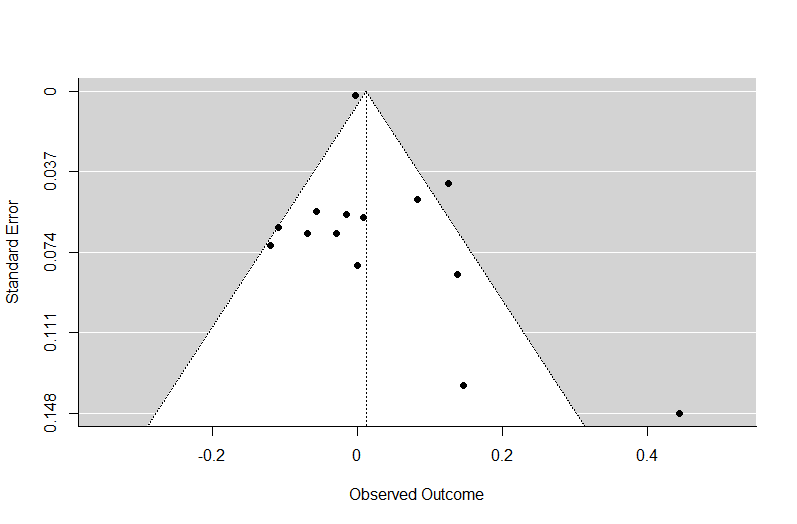


Appendix Figure 5. Funnel plot for time spent on domestic work


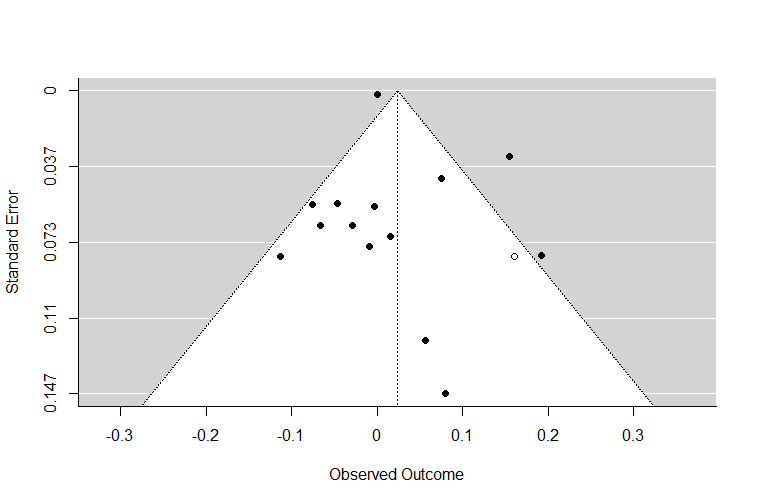


Appendix Figure 6. Funnel plot for time spent on leisure (hours)


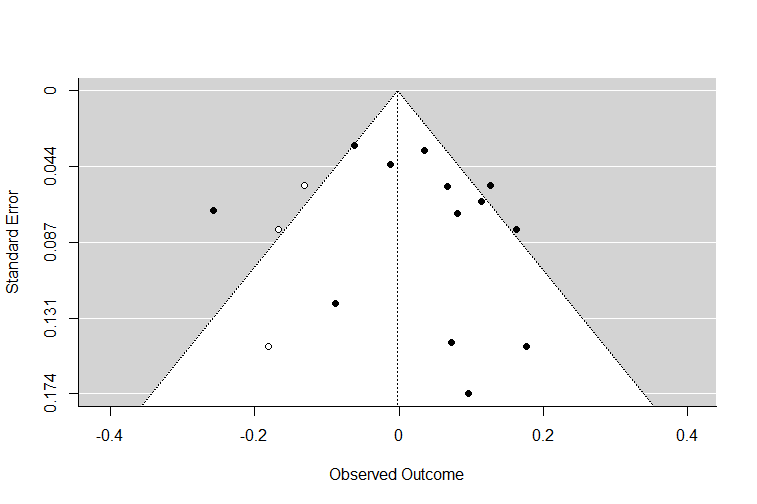


Appendix Figure 7. Funnel plot for time spent study (all)


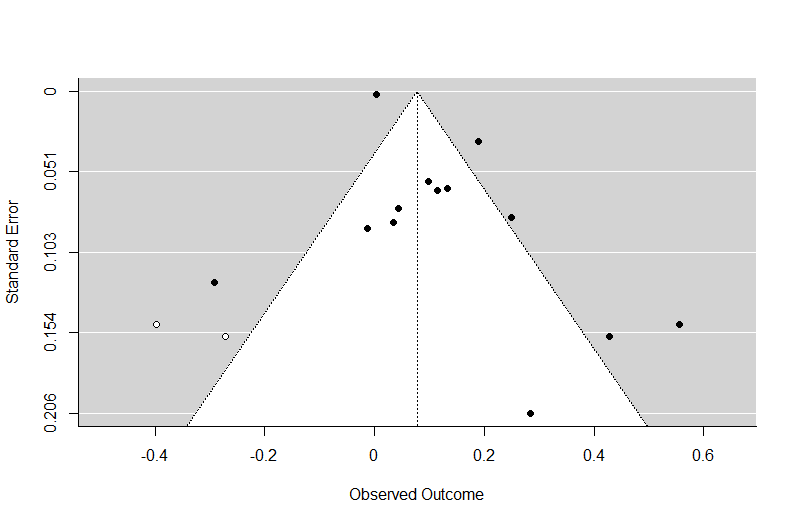


Appendix Figure 8. Funnel plot for time spent studying (children only)


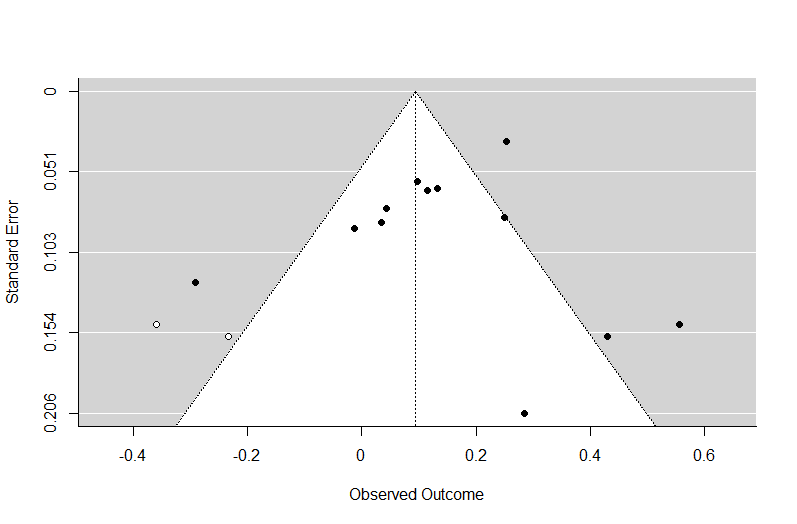


Appendix Figure 9. Funnel plot for respiratory illness


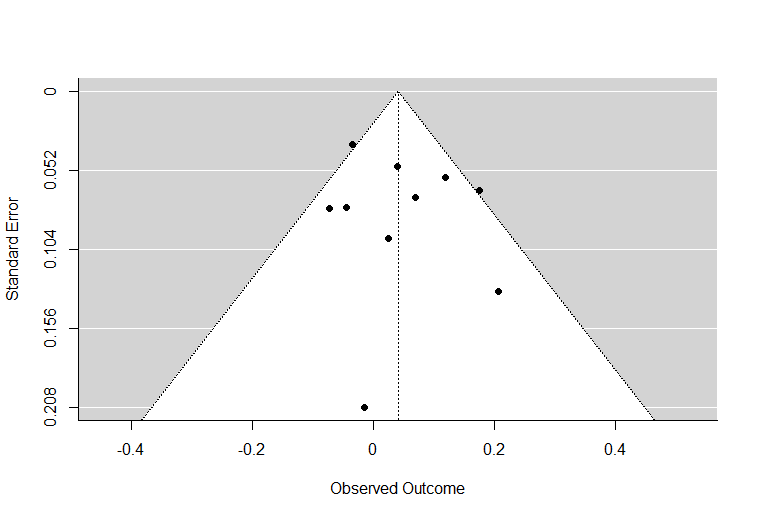


# Appendix J: Moderator analyses (full results)

Appendix Table 3. Full moderator results

| **Group** | **Category** | **Indicator** | **N** | **Pooled SMD** | **Region** | **Country** | **Technology** | **Technology Level** | **Mechanism** |
| --- | --- | --- | --- | --- | --- | --- | --- | --- | --- |
| Energy Access | Energy Security | **Access** | **3** | **0.2**** | NA | NA | NA | NA | NA |
|  |  | Reliability | 2 | 0.29 | NA | NA | NA | NA | NA |
|  |  | Affordability | 2 | -0.01 | NA | NA | NA | NA | NA |
|  | Energy Consumption | Hours of lighting use | 12 | 0.16 | No significant differences between Sub-Saharan Africa (k = 8) and the rest: b = -0.27, p = .34, nor between South Asia (k = 3) and the rest: b = -0.13, p = .68 | No significant differences between Kenya (k = 3) and the rest of countries: b = -0.19, p = .55; India (k = 2) and the rest: b = 0.13, p= .75; or Uganda (k = 2) and the rest: b = -0.03, p = .93. All other countries had only one study each | No significant differences between Solar home systems (SHS) (k = 6) and the rest of the technologies: b = 0.19, p = .49; nor between Solar lamp with mobile phone charger (k = 3) and the rest: b = 0.17, p = .61 | No significant differences between household/individual (k = 10) and community (k = 2): b = 0.55, p = .14 | No significant differences between each mechanism and the rest: Direct provision (k = 4): b = 0.01, p = .96; Financial (credit or subsidy) (k = 4): b = -0.28, p = .35 ; Multi-mechanism (k = 2): b = 0.51, p = .13; Opportunity to access/ market expansion (k = 2): b = 0.16, p = .26 |
|  |  | **Kerosene consumption (litres of kerosene purchased/consumed)** | **6** | **-0.57 ***** | Sub-Saharan Africa (k = 4): b = 0.76, p < .01. Reference group: South Asia (k = 2) | No significant difference between Kenya (k =3) and the other two countries (two from Bangladesh and one from Burkina Faso): b = 0.39, p = .28. The difference between Bangladesh and the rest is captured by the region. | No significant differences between Solar Home Systems (k = 4) and the rest (one micro-hydroelectric and one solar lamp): b = -0.11, p = .80 | Not enough variation | Financial plus information (k = 2): b = -0.92, p < 0.01. Reference group: financial alone (k = 2) |
|  | Energy Expenditure | Energy expenditure (all) | 18 | 0.15 | South Asia (k = 4) compared to Sub-Saharan Africa (k = 12): b = 0.59, p = .02. No significant difference compared to LAC (k = 2) | Uganda (k = 2): b = -0.96, p < .01 compared to all other countries. No significant differences were observed for Kenya (k = 6), India (k = 3), or Rwanda (k = 2). There was only one study each for all other countries. | Solar Home Systems (k = 6) compared to all other technologies: b = -0.59, p < .01. Solar lamp with phone charger (k = 5) compared to all other technologies: b = 0.55, p = .02. No significant differences for solar lamps without charger (k = 2) or solar mini- and microgrids (k = 3). | No significant differences between household/individual (k = 14) and community (k = 4): b = -0.04, p = .87 | Financial (credit or subsidy, k = 7): b = -0.54, p = .04. Reference group: direct provisions (k = 6). No other significant difference. |
|  | Technology Uptake | **Purchase/adoption of technology (compared to no intervention)** | 2 | **0.18***** | NA | NA | NA | NA | NA |
|  |  | **Purchase/adoption of technology (compared to lower price paid/higher subsidy)** | 3 | **-0.52 ***** | NA | NA | NA | NA | NA |
|  | Technology Usage | **Technology use: any (LED/solar light, solar stove, electricity)** | **5** | **0.61***** | NA | NA | NA | NA | Direct provision (k = 2) compared to all other mechanisms: b = 1.49, p = .02. |
|  |  | Technology use: light (LED/solar) | 3 | 0.24 | NA | NA | NA | NA | NA |
| Climate | Air quality | PM 2.5 | 5 | 0.16 | NA | NA | Solar lamp (k = 2) compared to solar cookstove (k = 2): b = 0.34, p = .04. | NA | No significant differences. Direct provision (n = 3): -0.10, p = 0.61. Reference group: financial (n = 1) and multi-mechanism (n = 1) |
| Socio-economic | Economic | **Household (or personal) income** | **13** | **0.06***** | No significant differences between Sub-Saharan Africa (k = 8) and the rest: b = 0.04, p = .43, nor between South Asia (k = 2) and the rest: b = -0.04, p = .52, or LAC (k = 2) and the rest: b = -0.08, p = .19 | No significant difference between Kenya (k =4) and the rest of countries: b = -0.02, p = .73; nor between Uganda (k = 2) and the rest of countries: b = 0.11, p = .08 | No significant differences between Solar home systems (k = 6) and the rest of the technologies: b = 0.03, p = .52; nor between Solar lamp (k = 2) and the rest: b = -0.09, p = .14, or minigrid (k = 2) and the rest: b = 0.03, p = .66 | No significant differences between household/individual (k = 10) and community (k = 3): b = 0.02, p = .67 | No significant differences between each mechanism and the rest: Direct provision (k = 3): b = -0.06, p = .25.; Financial (credit or subsidy) (k = 6): b = -0.01, p = .90 ; Multi-mechanism (k = 2): b = -0.003, p = .95. Only one study for each of the other mechanisms. |
|  | Allocation of Time | Time spent on domestic work | 14 | 0.02 | No significant differences between Sub-Saharan Africa (k = 8) and the rest (five from South Asia and one from LAC): b = -0.003, p = .95 | No significant differences between Kenya (k = 2) and the rest of countries: b = -0.06, p = .38; India (k = 2): b = 0.001, p= .99; Uganda (k = 2): b = -0.07, p = .29; or Bangladesh (k = 2) and the rest: b = 0.07, p = .27. All other countries had only one study each | No significant differences between Solar home systems (k = 5) and the rest of the technologies: b = 0.05, p = .34; nor between Solar lamp with charger (k = 4) and the rest: b = 0.01, p = .90; or solar lamp alone (k = 2) and the rest: b = -0.06, p = .38. Other technologies were assessed by single studies. | No significant differences between household/individual (k = 12) and community (k = 2): b = 0.02, p = .74 | No significant differences between each mechanism and the rest: Direct provision (k = 6): b = -0.03, p = .50; Financial (credit or subsidy) (k = 4): b = 0.02, p = .68; Multi-mechanism (k = 2): b = 0.05, p = .39; or opportunity to access/ market expansion (k = 2): b = -0.04, p = .61 |
|  |  | Time spent on leisure | 13 | 0.03 | No significant differences between Sub-Saharan Africa (k = 10) and the rest (two from South Asia and one form LAC): b = -0.03, p = .75 | No significant differences between Kenya (k = 5) and the rest of countries: b = -0.06, p = .40; or Rwanda (k = 2) and the rest: b = 0.06, p = .67. All other countries had only one study each | No significant differences between Solar home systems (k = 4) and the rest of the technologies: b = -0.06, p = .36; nor between Solar lamp with charger (k = 4) and the rest: b = 0.03, p = .72; or solar lamp alone (k = 3) and the rest: b = 0.08, p = .29. Other technologies were assessed by single studies. | No significant differences between household/individual (k = 11) and community (k = 2): b = 0.04, p = .68 | No significant differences between each mechanism and the rest: Direct provision (k = 5): b = 0.07, p = .37; Financial (credit or subsidy) (k = 5): b = -0.01, p = .92; or Multi-mechanism (k = 2): b = -0.04, p = .64. Only one study assessed the effect of an opportunity to access/ market expansion. |
|  |  | Time spent on leisure: resting/sleeping | 6 | -0.03 | Sub-Saharan Africa (k = 4) compared to the other two studies (one from South Asia and one from LAC): b = 0.17, p = .04 | No significant differences between Kenya (k = 3) and the rest of the countries: b = 0.13, p = .16. All other countries had only one study each | No significant differences compared to Solar home system (k =2), for solar lamp with mobile charger (k = 2): b = 0.19, p = .09, nor for solar lamp alone (k = 2): b = 0.16, p = .15 | NA | No significant differences between direct provision (k = 3) and other mechanisms (one multi-mechanism and two financial incentives): b = 0.12, p = .27 |
|  |  | Time spent working (remunerated) | 14 | 0.01 | No significant differences between Sub-Saharan Africa (k = 10) and the rest (three from South Asia and one from LAC): b = 0.01, p = .82 | No significant differences between Kenya (k = 3) and the rest of countries: b = -0.05, p = .44; India (k = 2): b = 0.09, p= .28; and Uganda (k = 2): b = -0.07, p = .31. All other countries had only one study each | Solar lamp with mobile phone charger (k = 3) compared to al other technologies: b = 0.15, p < .01. No significant difference compared to other technologies for SHS (k = 4): b = -0.09, p = .08; solar mini-grid (k = 2): b = -0.02, p = .78; and solar lamp alone (k = 2): b = -0.11, p = .08. All other technologies were assessed by single studies. | No significant differences between household/individual (k = 11) and community (k = 3): b = 0.02, p = .69 | No significant differences between each mechanism and the rest: Direct provision (k = 6): b = 0.04, p = .47; Financial (credit or subsidy) (k = 4): b = -0.01, p = .84; or the opportunity to access/ market expansion (k = 2): b = -0.06, p = .37. Only one study each assessed the effect of Multi-mechanisms and information. |
|  |  | **Time spent studying** | **13** | **0.11***** | No significant differences between Sub-Saharan Africa (k = 8) and the rest (four from South Asia and one from LAC): b = -0.12, p = .19 | No significant difference between Kenya (k = 4) and the rest of the countries: b = -0.17, p = .08. Bangladesh (k = 2): b = 0.12, p = .26; or Uganda (k = 2): b = -0.09, p = .46 | Solar lamp/ lantern with mobile phone charger (k = 3) compared to all other technologies: b = 0.27, p < .01.  SHS (k = 5) compared to micro-hydro (k = 2): b = 0.20, p = .02 | Household/individual level (k = 11): 0.24, p = .01. Reference group: community interventions (k = 2) | Opportunity to access/ market expansion (k = 2): -0.31, p = .04. Reference group: Direct provision (k=6) |
|  |  | **Time spent studying (children)** | **12** | **0.14***** | Sub-Saharan Africa (k = 8) compared to other regions: b = -0.19, p = .02 | Kenya (k = 4) compared to the rest of countries: b = -0.21, p = .03. No significant differences between Bangladesh (k = 2) and the rest of countries: b = 0.14, p = .24, or Uganda (k = 2) and the rest of countries: b = -0.12, p = .37. All other countries had only one study each | Solar lamp/ lantern with mobile phone charger (k = 3) compared to other technologies: b = 0.26, p = .03. No significant difference compared to other technologies for SHS (k = 5): b = 0.04, p = .72; solar lamp alone (k = 3): b = -0.10, p = .41. All other technologies were assessed by single studies. | NA | Opportunity to access/ market expansion (k = 2) compared to Direct provision (k = 5): b = -0.37, p < .01. No significant difference for financial mechanisms (k = 3): b = -0.17, p = .17, nor for multi-mechanisms (k = 2): b = -0.08, p = .52 |
|  | Education | School attendance | 6 | 0.03 | No significant differences between Sub-Saharan Africa (k = 3) and the rest (one from South Asia and two from LAC): b = -0.002, p = .98 | No significant differences between Kenya (k = 2) and the rest of countries: b = 0.10, p = .06. All other countries had only one study each | No significant differences between Solar home systems (k = 4) and the rest of the technologies: b = -0.06, p = .36; nor between Solar lamp with charger (k = 4) and the rest: b = 0.03, p = .72; or solar lamp alone (k = 3) and the rest: b = 0.08, p = .29. Other technologies were assessed by single studies. | NA | No significant differences between direct provision (k = 3) and other mechanisms (one multi-mechanism and two financial incentives): b = -0.05, p = .38 |
|  |  | (Aggregated) test scores | 5 | 0.00 | NA | No significant differences between Kenya (k = 3) and the rest of countries: b =-0.04, p = .50. All other countries had only one study each | No significant differences between Solar home systems (k = 3) and the rest of the technologies: b = -0.001, p = .98; Other technologies were assessed by single studies. | NA | No significant differences between each mechanism and the rest: Direct provision (k = 6): b = 0.04, p = .47; Financial (credit or subsidy) (k = 4): b = -0.01, p = .84; or the opportunity to access/ market expansion (k = 2): b = -0.06, p = .37. Only one study each assessed the effect of Multi-mechanisms and information. |
|  | Health | Respiratory illness | 10 | 0.04 | No significant differences between Sub-Saharan Africa (k = 8) and the rest (one from South Asia and two from LAC): b = 0.05, p = .40 | Uganda (k = 2) compared to the rest of countries: b = 0.17, p = .02. No significant differences between Malawi (k = 2) and the rest of countries: b = -0.09, p = .28. All other countries had only one study each | No significant differences compared to the rest of technologies for Solar home systems (k = 2): b = 0.01, p = .98; Solar improved cookstove (k = 2): b = -0.09, p = .28; solar lamp alone (k = 2) and the rest: b = 0.12, p = .11; or solar lamp with charger (k = 3): b = 0.01, p = .92. Other technologies were assessed by single studies. | NA | No significant differences between each mechanism and the rest: Direct provision (k = 6): b = -0.05, p = .47; Financial (credit or subsidy) (k = 3): b = 0.01, p = .93. Only one study assessed the effect of Multi-mechanisms. |
|  | Women’s Empowerment | **Women's empowerment** | **3** | **0.11***** | NA | NA | NA | NA | NA |

| **Group** | **Category** | **Indicator** | **N** | **Pooled SMD** | **Scale** | **Supply/ Demand focus** | **Target Actor** | **Target Population** | **Implementation agency** | **Design** | **Year of publication** | **Risk of bias rating** |
| --- | --- | --- | --- | --- | --- | --- | --- | --- | --- | --- | --- | --- |
| Energy Access | Energy Security | **Access** | **3** | **0.2**** | NA | NA | NA | NA | NA | NA | NA | NA |
|  |  | Reliability | 2 | 0.29 | NA | NA | NA | NA | NA | NA | NA | NA |
|  |  | Affordability | 2 | -0.01 | NA | NA | NA | NA | NA | NA | NA | NA |
|  | Energy Consumption | Hours of lighting use | 12 | 0.16 | No significant differences between regional (k = 7) and other scales: b = 0.42, p = .12; nor between national (k = 4) and the rest: b = -0.23, p = .44 | NA | No significant differences between households (k = 9) and other targeted actors: b = 0.33, p = .29 | No significant differences between targeting students (k = 2) and the rest of the studies not targeting a specific population: b = -0.35, p = .33 | No significant differences between for-profit firm (k = 5) and other implementation agencies: b = -0.25, p = .38; government agency (k = 3) and the rest: b = 0.20, p= .55, and non-profit organization (k = 2) and the rest: b = -0.24, p = .52 | No significant differences between experimental (k = 6) and quasi-experimental designs (k = 6): b = -0.21, p = .44 | No significant differences: b = 0.04, p = .50 | No significant differences between high-risk-of-bias studies (k = 10) and the rest (one low and one medium): b = -0.52, p = .14 |
|  |  | **Kerosene consumption (litres of kerosene purchased/consumed)** | **6** | **-0.57 ***** | No significant difference for regional (k = 2) when National (k = 4) is the reference group: b = 0.49, p = .18. | No significant differences between demand (k = 4) and supply (k = 2): b = 0.23, p = .60 | NA | NA | Government agency, with and without for-profit firm (k = 3): b = -0.74, p < .01, vs other type of agencies | NA | More recent studies present marginally larger estimates: b = 0.07, p = .03 | NA |
|  | Energy Expenditure | Energy expenditure (all) | 18 | 0.15 | No sig differences for regional (k = 10): b = 0.11, p = .72, or national (k = 4): b = 0.17, p = .64, compared to local scale (k = 4). | NA | No significant differences between households (k = 12) and other targeted actors: b = 0.005, p = .99 | No significant differences between targeting students (k = 3) and the rest of the studies not targeting a specific population: b = 0.29, p = .35 | For-profit implementation agencies (k = 7): b = -0.47, p = .02, compared to other types of agencies. No other significant differences were observed for government agencies (k = 5): b = 0.16, p = .53; and non-profit organizations (k = 4): b = 0.08, p = .75 | Quasi-experimental studies (k = 8): b =-0.45, p = .03. Reference group: experimental studies (k = 10) | No significant differences: b = -0.02, p = .70 | No significant differences between high-risk-of-bias studies (k = 14) and the rest (two low and two medium): b = -0.23, p = .40 |
|  | Technology Uptake | **Purchase/adoption of technology (compared to no intervention)** | 2 | **0.18 ***** | NA | NA | NA | NA | NA | NA | NA | NA |
|  |  | **Purchase/adoption of technology (compared to lower price paid/higher subsidy)** | 3 | **-0.52 ***** | NA | NA | NA | NA | NA | NA | NA | NA |
|  | Technology Usage | **Technology use: any (LED/solar light, solar stove, electricity)** | **5** | **0.61 ***** | NA | NA | NA | NA | No significant differences between non-profit organizations (k =2) and all other agency types (only one study each): b = -0.99, p = .27 | NA | No significant differences: b = 0.25, p = .41 | NA |
|  |  | Technology use: light (LED/solar) | 3 | 0.24 | NA | NA | NA | NA | NA | NA | NA | NA |
| Climate | Air quality | PM 2.5 | 5 | 0.16 | NA | NA | NA | NA | NA | NA | No significant differences (b = 0.021, p = 0.627) | NA |
| Socio-economic | Economic | **Household (or personal) income** | **13** | **0.06 ***** | No significant differences (national as the reference group, k = 6): Regional (k = 6): b = -0.03, p = .58, and local (k = 1): b = 0.02, p = .82 | NA | No significant differences between households (k = 9) and other targeted actors: b = -0.03, p = .60 | NA | No significant differences between for-profit firm (k = 3) and other implementation agencies: b = 0.09, p = .08; government agency (k = 7) and the rest: b = -0.07, p = .13, and non-profit organization (k = 2) and the rest: b = 0.03, p = .63 There was only one study looking at an intervention implemented by an international aid agency. | No significant differences between quasi-experimental (k = 10) and experimental designs (k = 4): b = - 0.01, p = .88 | No significant differences between for-profit firm (k = 3) and other implementation agencies: b = 0.09, p = .08; government agency (k = 7) and the rest: b = -0.07, p = .13, and non-profit organization (k = 2) and the rest: b = 0.03, p = .63 There was only one study looking at an intervention implemented by an international aid agency. | No significant differences: b = -0.001, p = .92 |
|  | Allocation of Time | Time spent on domestic work | 14 | 0.02 | No significant differences (regional as the reference group, k = 9): National (k = 3): b = 0.06, p = .12, and local (k = 2): b = 0.06, p = .37 | Demand (k = 12) significant difference compared to both (k = 2): b = -0.16, p < .01 | No significant differences between households (k = 8) and other targeted actors: b = -0.05, p = .47 | No significant differences between targeting students (k = 3) and not targeting a specific population (k = 10): b = -0.09, p = .12. Only one study assessing an intervention targeting women. | No significant differences between for-profit firm (k = 4) and other implementation agencies: b = -0.05, p = .39; government agency (k = 3) and the rest: b = 0.04, p = .49, and non-profit organization (k = 5) and the rest: b = 0.03, p = .56.There was only one study looking at an intervention implemented by a private foundation and one not specified. | No significant differences between quasi-experimental (k = 6) and experimental designs (k = 8): b = 0.04, p = .47 | No significant differences: b = -0.02, p = .12 | No significant differences when high-risk-of-bias studies (k = 9) are the reference group for medium (k = 3): b = 0.02, p = .76, or low (k = 2): b = 0.01, p = .93 |
|  |  | Time spent on leisure | 13 | 0.03 | Compared to national programs, regional programs had larger effects by b = 0.17, p < .01 | No significant differences between demand (k = 11) and both demand and supply (k = 2): b = 0.001, p = .99 | No significant differences between households (k = 10) and other targeted actors: b = -0.13, p = .07 | No significant differences between targeting students (k = 2) and the rest of the studies not targeting a specific population: b = 0.05, p = .56 | For-profit (k = 3) compared to other types of agencies: b = -0.17, p = .03. No significant differences between government agency (k = 4) and the rest: b = 0.003, p = .97, nor for non-profit organizations (k = 3) and the rest: b = 0.05, p = .53.There were only single studies looking at an intervention implemented by a private foundation, an international aid agency, or no agency specified. | No significant differences between quasi-experimental (k = 7) and experimental designs (k = 6): b = -0.05, p = .45 | No significant differences: b = 0.004, p = .73 | High-risk-of-bias studies (k = 10) compared to the rest of studies: b = -0.15, p = .04 |
|  |  | Time spent on leisure: resting/sleeping | 6 | -0.03 | No significant differences between national (k = 2) and regional (k = 4): b = -0.02, p = .85 | NA | No significant differences between households (k = 3) and other targeted actors: b = 0.08, p = .50 | No significant differences between targeting students (k = 3) and the rest of the studies not targeting a specific population: b = 0.13, p = .16 | No significant differences between national (k = 2) and regional (k = 4): b = -0.02, p = .85 | Quasi-experimental studies (k = 2) compared to experimental studies (k = 4): b = -0.17, p = .04. | No significant differences: b = 0.01, p = .11 | NA |
|  |  | Time spent working (remunerated) | 14 | 0.01 | Regional (k = 7) compared to National (k = 5) and local (k = 2): b = -0.09 , p = .03 | NA | No significant differences between households (k = 8) and other targeted actors: b = -0.03, p = .63 | No significant differences between targeting students (k = 2) and the rest of the studies not targeting a specific population: b = -0.11, p = .08 | Regional (k = 7) compared to National (k = 5) and local (k = 2): b = -0.09 , p = .03 | No significant differences between quasi-experimental (k = 7) and experimental designs (k = 7): b = -0.06, p = .25 | No significant differences: b = -0.01, p = .78 | No significant differences when high-risk-of-bias studies (k = 8) are the reference group for medium (k = 4): b = -0.01, p = .83, or low (k = 2): b = 0.10, p = .31 |
|  |  | **Time spent studying** | **13** | **0.11 ***** | No significant differences between National (k = 5) and regional (k = 8): b = 0.04, p = .69 | NA | No significant differences between households (k = 9) and other targeted actors: b = -0.08, p = .43 | No significant differences between targeting students (k = 3) and the rest of the studies not targeting a specific population: b = -0.01, p = .94 | No significant differences between government agencies (k = 6) and other implementation agencies: b = -0.06, p = .55; for-profit firms (k = 3) and the rest: b = -0.04, p = .71, and non-profit organization (k = 2) and the rest: b = 0.003, p = .98.There was only one study each looking at an intervention implemented by a private foundation or not specified. | No significant differences between quasi-experimental (k = 8) and experimental designs (k = 5): b = -0.12, p = .22 | No significant differences: b = -0.01, p = .40 | No significant differences between high-risk-of-bias studies (k = 10) and the rest (two medium-risk-of-bias and one low-risk of bias): b = -0.07, p = .56 |
|  |  | **Time spent studying (children)** | **12** | **0.14 ***** | No significant differences between National (k = 4) and regional (k = 8): b = -0.02, p = .87 | NA | No significant differences between sutides targetting households (k = 8) and the rest of the studies: b = -0.06, p = .63. Nor between studies targeting individuals (k = 2) and the rest: b = -0.03, p = .87 | No significant differences between targeting students (k = 3) and the rest of the studies not targeting a specific population: b = -0.04, p = .75 | No significant differences between government agencies (k = 5) and other implementation agencies: b = -0.02, p = .84; for-profit firms (k = 3) and the rest: b =,-0.03 p = ..87, and non-profit organization (k = 2) and the rest: b = -0.07, p = .59.There was only one study each looking at an intervention implemented by a private foundation or not specified. | No significant differences between quasi-experimental (k = 7) and experimental designs (k = 5): b = -0.10, p = .37 | No significant differences: b = -0.01, p = .32 | No significant differences between high-risk-of-bias studies (k = 10) and the rest (one medium-risk-of-bias and one low-risk of bias): b = -0.18, p = .21 |
|  | Education | School attendance | 6 | 0.03 | NA | NA | No significant differences between sutides targetting households (k = 3) and the rest of the studies: b = 0.01, p = .87. Nor between studies targeting individuals (k = 2) and the rest: b = 0.05, p = .54 | No significant differences between targeting students (k = 2) and the rest of the studies not targeting a specific population: b =0.05, p = .54 | No significant differences between government agencies (k = 3) and other implementation agencies: b = 0.05, p = .38; non-profit organization (k = 2) and the rest: b = 0.05, p = .54.There was only one study with implementation agency not specified. | No significant differences between quasi-experimental (k = 2) and experimental designs (k = 4): b = 0.01, p = .89 | No significant differences: b = 0.01, p = .67 | Some concerns (k = 2) compared to High RoB (k = 4): b = -0.12, p = .01 |
|  |  | (Aggregated) test scores | 5 | 0.00 | No significant differences between region (k = 3) and the rest of studies (one local and one national): b = -0.02, p = .67 | NA | No significant differences between individuals (k = 3) and other targeted actors: b = 0.02, p = .63 | Not enough variation (all but one are students) | No significant differences between charitable or private foundation (k = 2) and other implementation agencies: b = 0.01, p = .90; non-profit organization (k = 2) and the rest: b = -0.01, p = .93.There was only one study with a government agency as the implementation agency. | NA | No significant differences: b = 0.01, p = .55 | No significant differences between high-risk-of-bias studies (k = 3) and the rest (one medium-risk-of-bias and one low-risk of bias): b = -0.04, p = .50 |
|  | Health | Respiratory illness | 10 | 0.04 | No significant differences between region (k = 5) and the rest of studies (four local and one national): b = 0.03, p = .68 | NA | No significant differences between individuals (k = 7) and other targeted actors: b = 0.08, p = .11 | No significant differences between not targeting either students or women (k = 3) and the rest of the studies not targeting a specific population: b =0.02, p = .75 | No significant differences between non-profit organization (k = 3) and other implementation agencies: b = -0.03, p = .70; nor between for-profit firm (k = 2) and the rest: b = 0.13, p = .09.There was only one study with a government agency as the implementation agency, one with a charitable or private foundation and the rest did not specify the implementation agency. | No significant differences between quasi-experimental (k = 2) and experimental designs (k = 8): b = 0.01, p = .89 | No significant differences: b = 0.003, p = .76 | NA |
|  | Women’s Empowerment | **Women's empowerment** | **3** | **0.11 ***** | NA | NA | NA | NA | NA | NA | NA | NA |

Notes:

N is the number of effect sizes included in the analysis.

"NA" stands for not available for cases in which there was not enough variation, either because all included studies had the same outcome category, or any of the categories did not have at least two observations.

"NSD" stands for "No significant difference" between categories of a moderator.

# Appendix reference list

Aloe, A. M., Becker, B. J., Duvendack, M., Valentine, J. C., Shemilt, I., & Waddington, H. (2017). Quasi-experimental study designs series—paper 9: Collecting data from quasi-experimental studies. *Journal of Clinical Epidemiology*, *89*, 77–83. <https://doi.org/10.1016/j.jclinepi.2017.02.013>

Gillani, A. A., Khan, S., Nasir, S., & Niaz, S. (2022). The effectiveness of installing solar panels at schools in Pakistan to increase enrolment. *Journal of Environmental Studies and Sciences*, *12*(3), 505–514. <https://doi.org/10.1007/s13412-022-00747-z>

Homan, T., Hiscox, A., Mweresa, C. K., Masiga, D., Mukabana, W. R., Oria, P., Maire, N., Pasquale, A. D., Silkey, M., Alaii, J., Bousema, T., Leeuwis, C., Smith, T. A., & Takken, W. (2016). The effect of mass mosquito trapping on malaria transmission and disease burden (SolarMal): A stepped-wedge cluster-randomised trial. *The Lancet*, *388*(10050), 1193–1201. <https://doi.org/10.1016/S0140-6736(16)30445-7>

Kishore, A., Joshi, P., & Pandey, D. (2017). Harnessing the sun for an evergreen revolution: A study of solar-powered irrigation in Bihar, India. *Water International*, *42*(3), 291–307. <https://doi.org/10.1080/02508060.2017.1312085>

Matavel, C., Kächele, H., Steinke, J., Rybak, C., Hoffmann, H., Salavessa, J., Sieber, S., & Müller, K. (2022). Effect of passive solar drying on food security in rural Mozambique. *Scientific Reports*, *12*(1), 17154. <https://doi.org/10.1038/s41598-022-22129-9>

Nagwekar, N. N., Tidke, V. B., & Thorat, B. N. (2020a). Seasonal Nutritional Food Security to Indian Women through Community-level Implementation of Domestic Solar Conduction Dryer. *Ecology of Food and Nutrition*, *59*(5), 525–551. <https://doi.org/10.1080/03670244.2020.1752686>

Nagwekar, N. N., Tidke, V. B., & Thorat, B. N. (2020b). Seasonal Nutritional Food Security to Indian Women through Community-level Implementation of Domestic Solar Conduction Dryer. *Ecology of Food and Nutrition*, *59*(5), 525–551. <https://doi.org/10.1080/03670244.2020.1752686>

Reeves, B. C., Wells, G. A., & Waddington, H. (2017). Quasi-experimental study designs series—paper 5: A checklist for classifying studies evaluating the effects on health interventions—a taxonomy without labels. *Journal of Clinical Epidemiology*, *89*, 30–42. <https://doi.org/10.1016/j.jclinepi.2017.02.016>

Rokicki, S., Mwesigwa, B., Schmucker, L., & Cohen, J. L. (2019). Shedding light on quality of care: A study protocol for a randomized trial evaluating the impact of the Solar Suitcase in rural health facilities on maternal and newborn care quality in Uganda. *BMC Pregnancy and Childbirth*, *19*(1), 306. <https://doi.org/10.1186/s12884-019-2453-x>

Sovacool, B. K., Axsen, J., & Sorrell, S. (2018). Promoting novelty, rigor, and style in energy social science: Towards codes of practice for appropriate methods and research design. *Energy Research & Social Science*, *45*, 12–42. <https://doi.org/10.1016/j.erss.2018.07.007>

Xu, H., Dong, R., Cui, Y., & Zang, W. (2022). Does the Photovoltaic poverty alleviation project promote county economic development? : Evidence from 852 counties in China. *Solar Energy*, *248*, 51–63. <https://doi.org/10.1016/j.solener.2022.10.050>

1. Data available at <https://datahelpdesk.worldbank.org/knowledgebase/articles/906519-world-bank-country-and-lending-groups> [↑](#footnote-ref-2)
2. We also included studies from countries that held high-income status for one year before reverting to L&MIC status. At the time of writing this, this exception only applied to Argentina (2014, 2017), Venezuela (2014), Mauritius (2019) and Romania (2019).  [↑](#footnote-ref-3)
3. For coding this information we used the following source: https://ec.europa.eu/eurostat/statistics-explained/index.php?title=Glossary:Renewable_energy_sources [↑](#footnote-ref-4)
4. For the qualitative studies, we use a slightly different language to scale the critical appraisal assessments as compared to the quantitative studies. The far right rating column always reflects a ‘critical’ appraisal judgement (i.e. ‘unreflective research’ above) with judgements moving further to the left on a scale from high to low critical appraisal. [↑](#footnote-ref-5)
