# Supplementary material for: Improving Energy Access, Climate and Socio‐Economic Outcomes Through Off‐Grid Electrification Technologies: A Systematic Review
Source: Campbell Syst Rev. 2025 Aug 15;21(3):e70060. doi: 10.1002/cl2.70060 (PMC12355195; doi:10.1002/cl2.70060)
Supplement: Supplementary file 2 — supmat.docx. [file CL2-21-e70060-s001.docx]

**Abbreviations**

| CAPS | Cooking and Pneumonia Study |
| --- | --- |
| CH4 | Methane |
| CO2 | Carbon dioxide |
| DEP | Development Evidence Portal |
| DID | Difference-in-Differences |
| EGM | Evidence Gap Map |
| ERSEN | Rural Electrification Senegal |
| GHG | Greenhouse gas emissions |
| GRADE | Grading of Recommendations, Assessment, Development, and Evaluation |
| ICS | Improved cookstoves |
| IE | Impact evaluation |
| ITS | Interrupted time series |
| L&MIC | Low- and middle-income country |
| LAC | Latin American and the Caribbean |
| N2O | Nitrous oxide |
| NGO | Non-governmental organisation |
| NOX | Nitrogen oxides |
| PM2.5 | Particulate matter |
| PRISMA | Preferred Reporting Items for Systematic reviews and Meta-Analyses |
| PV | Solar photovoltaics |
| PVPA | Photovoltaic Poverty Alleviation |
| QED | Quasi-experimental design |
| RCT | Randomised controlled trial |
| RDD | Regression discontinuity design |
| IV | Instrumental variables |
| SEforALL | Sustainable Energy for All |
| SDG | Sustainable Development Goal |
| SHS | Solar-home systems |
| SMD | Standardised mean differences |
| SMG | Solar market garden |
| SOX | Sulphur oxides |
| SSA | Sub-Saharan Africa |
| TWFE | Two-way fixed-effects |
| TWM | Two-way Mundlak |
| USD | United States dollar |
